# Total Synthesis of Ussuriedine via a Late-Stage Stevens Rearrangement: Implications for Biosynthesis

**DOI:** 10.1021/jacs.6c00926

**Published:** 2026-04-03

**Authors:** Daler Baidilov, Kyle J. Cassaidy, Yun-Jeong Shin, Viresh H. Rawal

**Affiliations:** Department of Chemistry, 189299 University of Chicago, Chicago, Illinois 60637, United States

## Abstract

Ussuriedine, an intricate heptacyclic steroidal alkaloid from the *Veratrum* family, features an unprecedented octahydro-3,6-methanoquinolizine framework. Its biosynthetic origins have not been established, and until now, no chemical synthesis has been reported. Here, we detail a de novo, convergent total synthesis of ussuriedine accomplished in 23 linear steps from ethyl vinyl ketone. The decalin (AB rings) and piperidine (F ring) fragments are coupled via reductive amination, establishing the connectivity required for an intramolecular [2 + 2 + 2] cyclotrimerization, which builds the aromatic D ring, thereby simultaneously constructing the C and E rings of the hexacyclic cevanine core. In the crucial final sequence, nitrogen quaternization followed by a [1,2]-Stevens rearrangement assembled the G ring, and selective oxidation of benzylic methine completed the natural product. The underlying chemical logic of the endgame, which connects the peripheral methyl and benzylic carbons, provides a mechanistic basis for a hypothesis on how nature might create ussuriedine’s complex framework and underscores the power of chemical synthesis to inform natural product biosynthesis.

## Graphical abstract

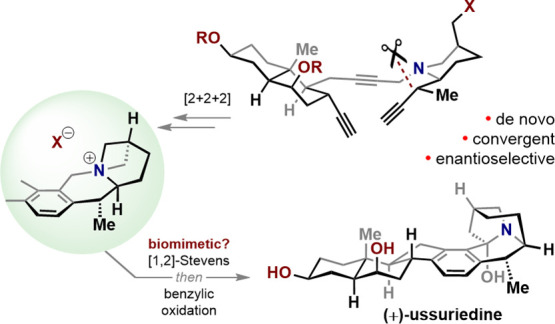

Every so often, natural product chemists encounter “artifacts”—compounds arising from nonenzymatic transformations that take place during isolation, analysis, storage, or related processes.^1^ These transformations span a broad spectrum of complexity. At one end lie simple reactions such as hydrolysis of carboxylic esters; at the other, intricate rearrangements that give rise to artifacts illuminating fundamental reactivity principles and challenging our understanding of biosynthetic logic. On occasion, such discoveries inspire biomimetic syntheses—routes that mirror nature’s elegantly designed enzymatic pathways that rapidly advance the complexity of simple building blocks.^2^ This study was motivated by one such isolation artifact and illustrates an example of total synthesis moving beyond its traditional roles of structural confirmation and biosynthetic validation to serve as a framework for generating biosynthetic hypotheses.^3^

## Introduction

In 1988, Kaneko and co-workers reported the isolation of ussurienine (**2**), a heptacyclic cevanine alkaloid from *Fritillaria ussuriensis*, cultivated in Heilongjiang Province, China (Scheme 1).^4^ Its structure, confirmed by X-ray crystallography, revealed an architectural novelty: unlike other cevanines, ussurienine features a methylene bridge connecting C25 to the benzylic C18 position, generating an additional G ring. The EF rings necessarily adopt a cis-quinolizidine arrangement, wherein the nitrogen lone pair cannot undergo pyramidal inversion and is locked cis to the α-oriented hydrogen at C22. The distinctive EFG ring system (tricyclo-[4.4.0.14,7]-6-aza-undecane), not previously observed in a natural product, is remarkably unstrained despite its structural rigidity.^5^ A year later, however, Kaneko published a full account clarifying that ussurienine was in fact an isolation artifact derived from the corresponding natural alkaloid ussuriedine (**1**).^6^

**Scheme 1:**
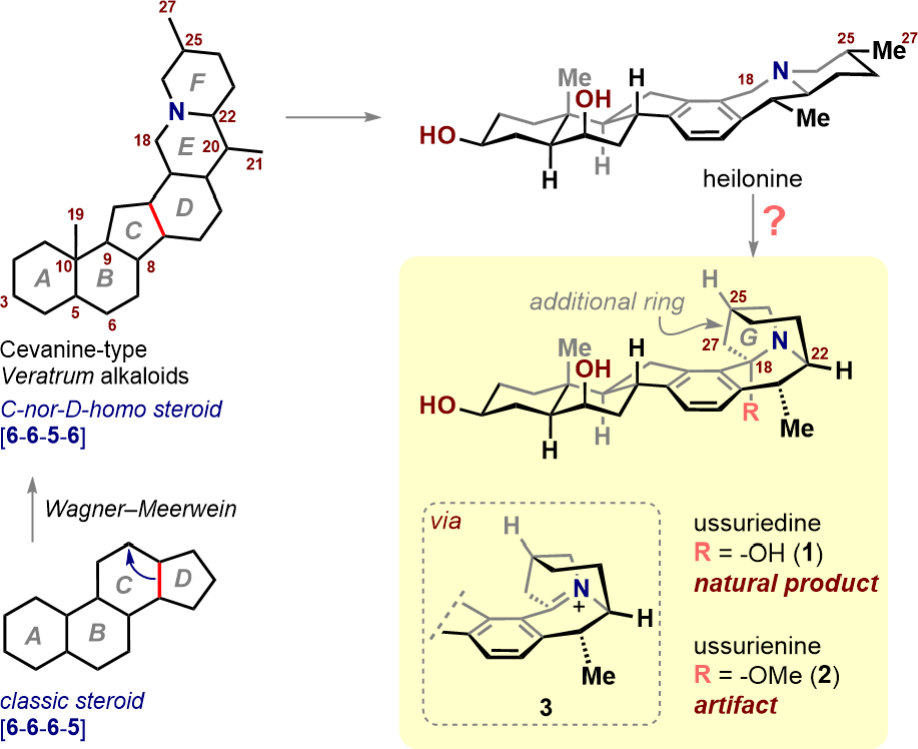
Cevanine-Type *Veratrum* Alkaloids: Heilonine, Ussuriedine, and Ussurienine

On the surface, the transformation appears straightforward—a simple solvolysis of the hemiaminal. Yet this mechanistic narrative conceals a fundamental difficulty, since the conversion of **1** into **2** during isolation would require iminium intermediate **3**, having a double bond embedded within a bridged tricyclic skeleton. Although this exact framework has not been investigated, analogous structures are unstable and are considered to be anti-Bredt alkenes due to the geometric distortion of the π-bond to a bridgehead atom.^7^ Thus, any synthetic strategy toward ussuriedine that proceeds through iminium **3** must confront the intrinsic complications associated with such strained intermediates.^8^ This potential issue notwithstanding, deliberately targeting iminium **3** has some allure, as it provides an opportunity to explore the limits of unconventional synthetic strategies.

This decade has witnessed several elegant syntheses of *Veratrum* natural products by the groups of Luo,^9^ Baran,^10^ Zhu/Gao,^11^ Dai,^12^ Liu/Qin,^13^ and Trauner,^14^ yet ussuriedine has eluded synthetic efforts until now. Moreover, while the biosynthesis of cevanine alkaloids is largely understood,^15^ no biosynthetic rationale has been proposed for ussuriedine. The co-occurrence of ussuriedine and heilonine in the same plant may suggest a shared biogenetic origin (Scheme 1);^16^ however, conventional biosynthetic pathways do not offer a mechanistic link between these compounds. Given our group’s interest in *Veratrum* alkaloids^17^ and inspired by ussuriedine’s unprecedented azatricyclic skeleton, we investigated the employment of a [1,2]-Stevens rearrangement late in the synthesis to forge the defining C–C bond of ussuriedine. The high efficiency of this skeletal reorganization likely offers insight into how nature may construct the polycyclic cage portion of the natural product.

## Results and Discussion

### Retrosynthetic Analysis

Several approaches were considered for the synthesis of the unique azatricyclic portion of ussuriedine. Since the amine is a part of a hemiaminal, a straightforward strategy could involve formation of the azatricycle by a late-stage transannular addition of the piperidine nitrogen of **4** to the benzylic ketone (Scheme 2, path A). Although this approach offers a direct route to **1**, we aimed for a more ambitious solution, one that transformed a heilonine-type precursor into the ussuriedine framework. We envisioned introduction of the hydroxyl in **1** from a putative iminium species **3** via a site-selective oxidation of **5** at the benzylic C18 carbon, in preference to two other possible sites (Scheme 2, path B). Given the stated concerns regarding Bredt’s rule, this oxidation had the potential to prove problematic.^7^ The uncommon azatricyclic scaffold of **5** could be assembled through the idealized scenario involving the union of the primary C27 radical with the benzylic C18 radical (**6**). Further consideration made clear that the required diradical intermediate represented the homolytic cleavage product of ylide **7**, itself accessible by chemoselective deprotonation of azetidinium salt **8**. The overall process constitutes an ammonium [1,2]-Stevens rearrangement,^18^ a transformation that, unlike its [2,3] counterpart, has seen less use in natural product synthesis.^19^ Our primary concerns with this strategy included (a) unselective homolytic cleavage of the two pseudosymmetric C–N bonds (C27–N vs C26–N) in **7**; (b) conformational change following C27–N bond cleavage to the trans quinolizidine, which would prevent the primary radical from combining with the benzylic radical; and (c) possible dissociation of the C18–N bond in ylide **7** to give a benzylic carbene, which could give untoward reactions. To allay concerns about fragmentation selectivity, we examined the energy-minimized equilibrium geometry structure of the azetidinium fragment **8′** (Spartan’24, Gaussian 6–31G* basis set, ωB97X-D), which showed the C27–N bond to be stereoelectronically aligned for scission. Despite other potential complications, we pushed forward given the conceptual simplicity of the [1,2]-Stevens disconnection and the opportunity to explore the capability of this underutilized transformation.

**Scheme 2:**
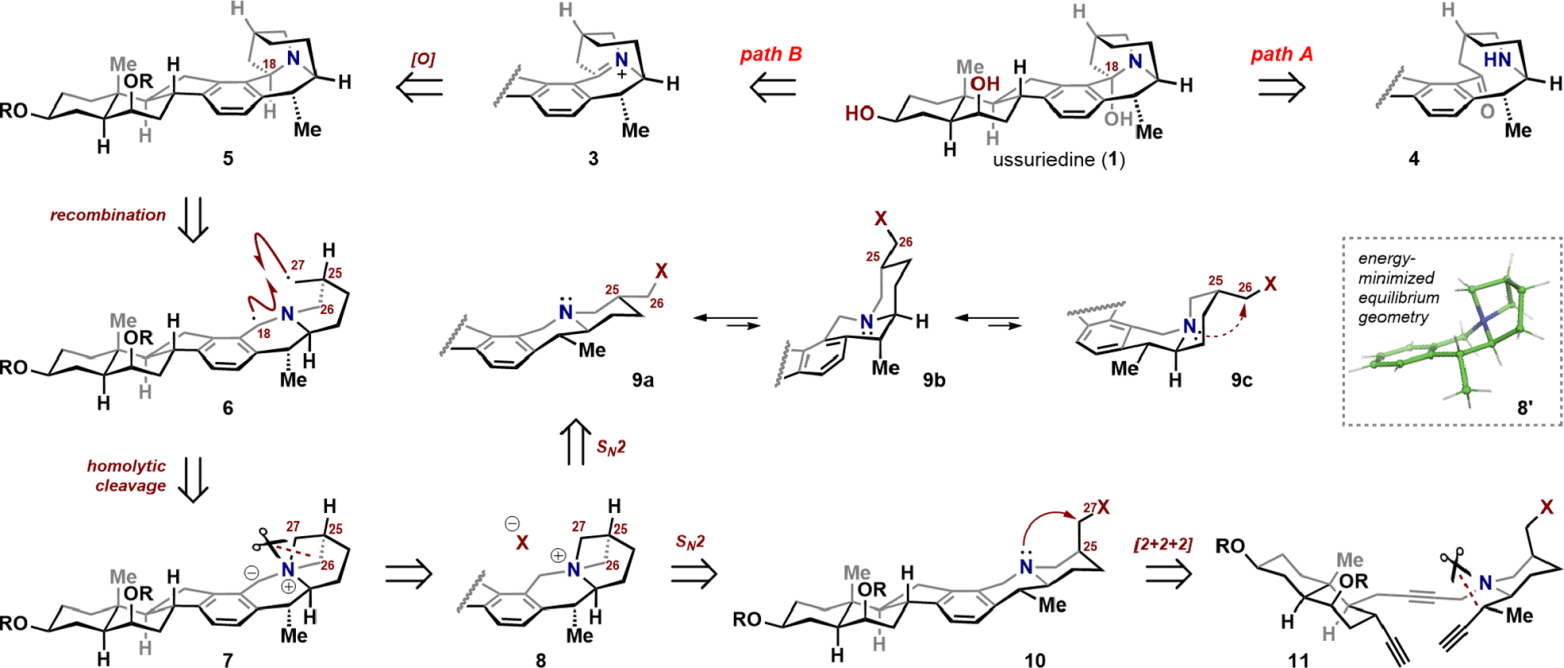
Retrosynthetic Analysis

At this point, the retrosynthetic problem can be reduced to the synthesis of azetidinium salt **8**, which could be accessed from cevanine-type intermediates **9** or **10**. The problem with **9**, however, is that the quinolizidine would exist primarily as the lower-energy trans conformer **9a**, with the two alkyl substituents equatorial and the nitrogen lone pair misaligned for the required S_N_2 reaction (cf. Scheme 2, **9c**). We elected to circumvent the conformational issue by pursuing intermediate **10**, having the alkylating unit in the more demanding axial orientation. Finally, as in our heilonine synthesis,^17^ we envisioned using the intramolecular [2 + 2 + 2] alkyne trimerization of **11**, which would form three C–C bonds and three rings in a single step.^20^ This powerful transformation offered a point of convergence, as triyne **11** could be traced back to two fragments of comparable complexity: the decalin (AB rings) and the piperidine (F ring) units.

### Synthesis of the Decalin Coupling Partner

The decalin coupling partner was prepared through a streamlined refinement of our previous route to a similar unit,^17^ affording cyclic ketal **12** in 9 steps and 17% overall yield from ethyl vinyl ketone, and providing the requisite intermediate containing all carbon units for the [2 + 2 + 2] cyclotrimerization with excellent enantio- and diastereocontrol (Scheme 3).^21^ After deprotection, the C6 hydroxy group was installed on enone **13** via initial formation of the dienol acetate, which simultaneously masked the propargyl alcohol as an acetate, followed by epoxidation in buffered dioxane. Treatment of the resulting γ-hydroxy enone **14** with in situ-generated HI induced deconjugative isomerization of the enone double bond revealing the 1,4-diketone, the direct reduction of which with borohydride gave diol **15** as a single diastereomer. The hydroxy groups were protected as TBS ethers, and the propargyl alcohol was unmasked in the same vessel using methanolic K_2_CO_3_. Finally, Dess–Martin periodinane oxidation delivered the fully elaborated coupling partner, propargyl aldehyde **17**.

**Scheme 3:**
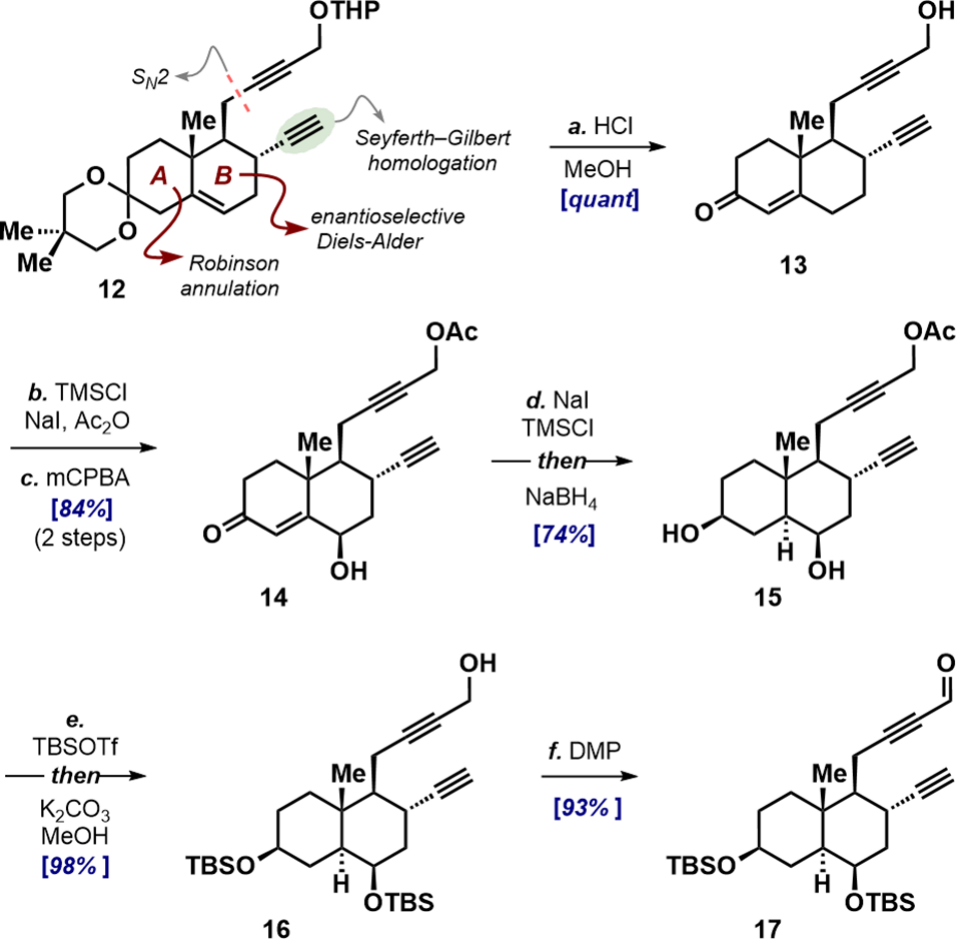
Synthesis of Propargyl Aldehyde **17** ^a^

a Reagents and conditions: *a.* 6.0 M aq. HCl (20 equiv), MeOH, 0 °C to rt, 3 h (quant); *b.* NaI (20 mol %), TMSCl (24 mol %), Ac_2_O, rt, 10 h; *c.* mCPBA (1.1 equiv), 0.1 M aq. phosphate buffer, 1,4-dioxane, 0 °C to rt, 18 h (84% yield, 2 steps); *d.* NaI (10 mol %), TMSCl (20 mol %), MeCN, 0 °C, 1 h; *then* Et_3_N (1 equiv), 0 °C, 10 min; *then* NaBH_4_ (4 equiv), MeOH, 0 °C, 50 min (74% yield); *e.* TBSOTf (2.6 equiv), 2,6-lutidine (3 equiv), CH_2_Cl_2_, 0 °C to rt, 12 h; *then* K_2_CO_3_ (4 equiv), MeOH, rt, 6 h (98% yield); *f.* DMP (1.1 equiv), CH_2_Cl_2_, rt, 10 min (93% yield). TMSCl = trimethylsilyl chloride; Ac_2_O = acetic anhydride; mCPBA = *m*-chloroperoxybenzoic acid; TBSOTf = *t*-butyldimethylsilyl trifluoromethanesulfonate; DMP = Dess–Martin periodinane.

### Synthesis of the Piperidine Coupling Partner

Having established an efficient route to the fully functionalized decalin core, we directed our efforts to the piperidine fragment (Scheme 4). The major challenge was stereoselective installation of the axially disposed hydroxymethyl unit on the readily available piperidone **18**.^17^ To this end, the secondary lactam in **18** was converted to ketone surrogate **19** via *N*-benzoylation, which forced the 3-butynyl unit into an axial orientation to avoid A^1,3^ strain. The titanium enolate **20** (**19**, TiCl_4_, DIPEA, CH_2_Cl_2_, 0 °C, 1 h) was then treated with gaseous formaldehyde at −78 °C, giving **21** as a single diastereomer.^22^ The high selectivity observed for the reaction reflects steric and electronic preference for axial addition to formaldehyde, away from the axially oriented 2-butynyl side chain. Epimerization of the hydroxymethyl fragment presented a significant challenge owing to the potential for elimination. Remarkably, treatment of a 0.1 M solution of trans-carbinol **21** in cold dichloromethane with excess DBU for 6 h cleanly furnished a 1:1 mixture of **21** and **22** in good yield. On the other hand, prolonged reaction times resulted in a complex mixture that included the vinylidene product arising via β-elimination of water. The two epimers were readily separated chromatographically, and recovered **21** was resubjected to the same conditions, furnishing cis-carbinol **22** in 81% yield after three cycles. The primary alcohol in **22** was then protected as its THP ether followed by hydrazine-mediated cleavage of the benzamide to yield secondary lactam **24**. Initial attempts to cleave the benzamide in **23** with methanolic hydrazine resulted in partial reduction of the alkyne unit. This side reaction was attributed to trace diimide formation from hydrazine. Addition of excess propargyl alcohol as a diimide scavenger circumvented this issue. Finally, reduction of lactam **24** delivered piperidine **25**, setting the stage for fragment union.

**Scheme 4:**
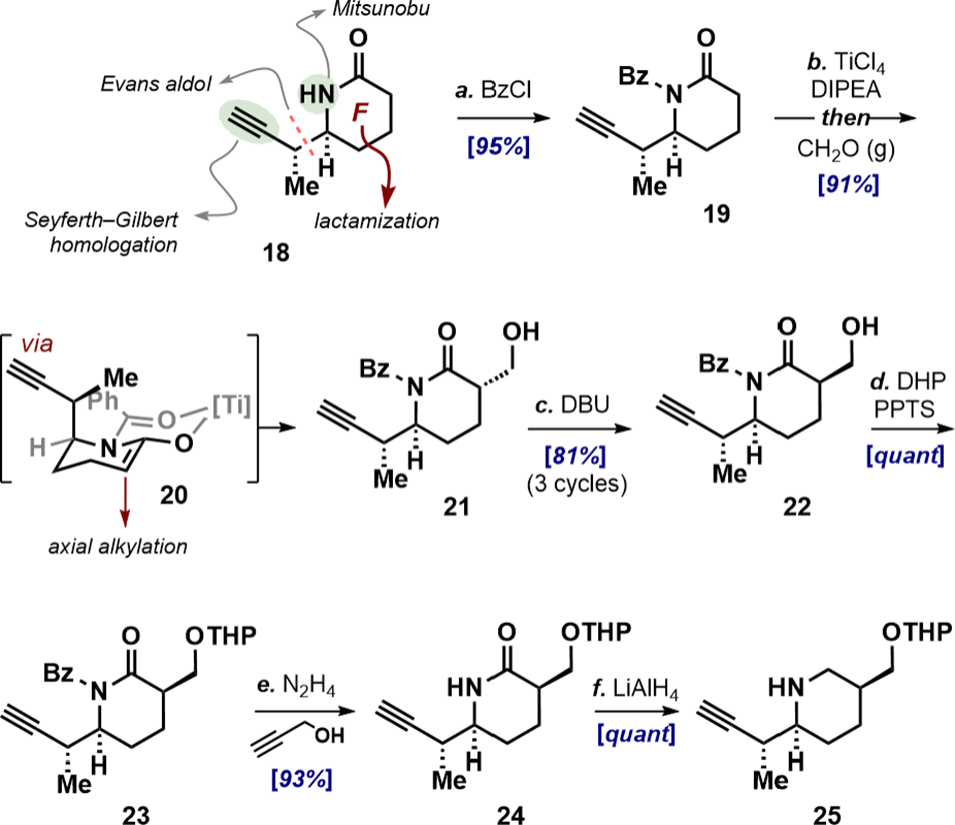
Synthesis of Piperidine **25** ^a^

a Reagents and conditions: *a.* BzCl (2.5 equiv), Et_3_N (3 equiv), DMAP (10 mol %), MeCN, 90 °C, 24 h (95% yield); *b.* TiCl_4_ (1.1 equiv), CH_2_Cl_2_, 0 °C, 10 min; *then* DIPEA (1.2 equiv), 0 °C, 1 h; *then* CH_2_O (g), −78 °C, 10 min (91% yield); *c.* DBU (3 equiv), CH_2_Cl_2_, 0 °C, 6 h (81% yield, 3 cycles); *d.* DHP (1.5 equiv), PPTS (20 mol %), CH_2_Cl_2_, rt, 48 h (quant); *e.* N_2_H_4_·H_2_O (3 equiv), propargyl alcohol (10% v/v), MeOH, 0 °C, 3 h (93% yield); *f.* LiAlH_4_ (5 equiv), THF, 70 °C, 20 h (quant). BzCl = benzoyl chloride; DMAP = 4-dimethylaminopyridine; DIPEA = *N*,*N*-diisopropylethylamine; DBU = 1,8-diazabicyclo[5.4.0]­undec-7-ene; DHP = 3,4-dihydro-2*H*-pyran; PPTS = pyridinium *p*-toluenesulfonate; THP = 2-tetrahydropyranyl; THF = tetrahydrofuran.

### Fragment Union

The two building blocks were brought together via a reductive amination: treatment of a 1:1 solution of **17** and **25** in dichloroethane with sodium triacetoxyborohydride gave triyne **26** within 10 min in 63% yield (85% brsm), setting the stage for the cycloisomerization reaction (Scheme 5).^23^ Despite the presence of a free amine, which we feared might deactivate the catalyst, amine-triyne **26** underwent the pivotal [2 + 2 + 2] cyclization, forging the CDE rings of the natural product and delivering hexacycle **27** in just two steps from its constituent fragments. With cevanine **27** in hand, we advanced to the final stage of the synthesis, designed to probe the feasibility of the [1,2]-Stevens shift that initially inspired this work.

**Scheme 5:**
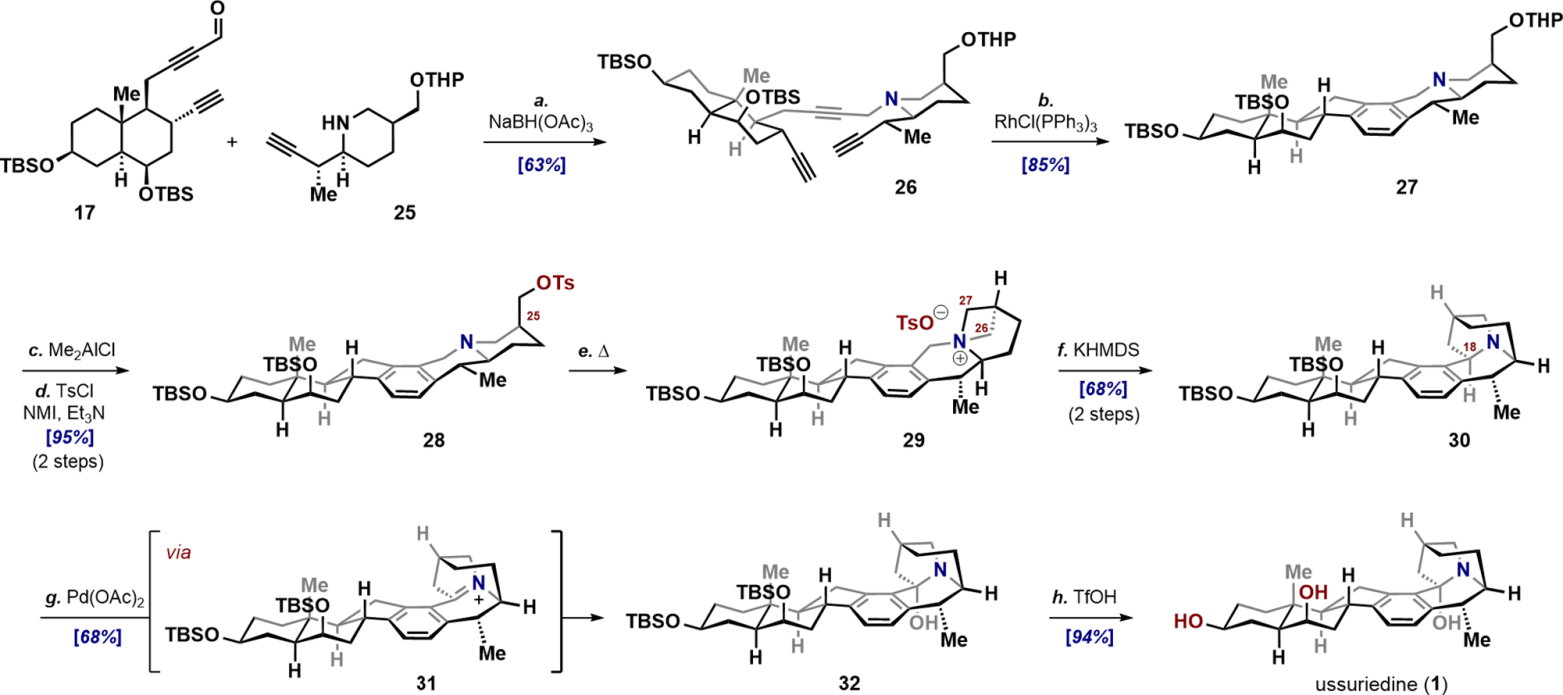
Endgame: Total Synthesis of Ussuriedine^a^

a Reagents and conditions: ***a.*** aldehyde **17** (1 equiv), piperidine **25** (1 equiv), NaBH­(OAc)_3_ (1.5 equiv), DCE, rt, 10 min (63% yield); ***b.*** RhCl­(PPh_3_)_3_ (10 mol %), EtOH, 60 °C, 1 h (85% yield); ***c.*** Me_2_AlCl (4 equiv), CH_2_Cl_2_, 0 °C to rt, 6 h; ***d.*** TsCl (2 equiv), NMI (3 equiv), Et_3_N (6 equiv), MeCN/CH_2_Cl_2_ = 4:1, rt (95% yield, 2 steps); ***e.*** MeCN (0.0017 M), 90 °C, 13 h; ***f.*** KHMDS (2 equiv), THF, −78 to 0 °C, 20 min (68%, 2 steps); ***g.*** Pd­(OAc)_2_ (20 mol %), KOAc (4 equiv), NBS (2 equiv), MeCN, 50 °C, 2 h (68% yield); ***h.*** TfOH (40 equiv), CH_2_Cl_2_, −78 to −40 °C, 10 min (94% yield). DCE = 1,2-dichloroethane; TsCl = *p*-toluenesulfonyl chloride; NMI = *N*-methylimidazole; KHMDS = potassium bis­(trimethylsilyl)­amide; THF = tetrahydrofuran; NBS = *N*-bromosuccinimide; TfOH = trifluoromethanesulfonic acid.

### Endgame

In preparation for the Stevens, the protected primary alcohol of **27** was converted into tosylate **28** by a two-step sequence: Me_2_AlCl-mediated chemoselective acetal cleavage followed by sulfonylation (cf. Scheme 2, **10**). A solvent screen and reaction conditions optimization showed that heating a dilute solution of **28** in MeCN for 13 h smoothly effected intramolecular *N*-alkylation, giving the azetidinium salt **29** in ca. 60% conversion. Intriguingly, prolonged heating resulted in apparent epimerization at C25, likely arising from opening of the azetidinium unit by tosylate attack at C26, affording the diastereomeric tosylate. Two key observations proved decisive in improving the reaction: (a) dilution of the reaction mixture (to 0.0017M) increased conversion to ca. 70% while suppressing “epimerization” and minimizing intermolecular reactions; and (b) the α-epimer did not cyclize under the reaction conditions thereby corroborating the initial mechanistic hypothesis for azetidinium formation. The optimal strategy, then, was to stop the reaction at partial conversion and attempt the Stevens rearrangement on the mixture of **29** and residual **28**. To our delight, dissolving this mixture in cold THF and treating it with KHMDS (2 equiv) smoothly furnished the aza-undecane **30**, with recovery of unreacted tosylate **28**. The successful outcome of the Stevens confirms the stereoelectronic preference for selective scission of the C27–N bond over the C26–N bond—a point of concern noted at the outset of this work—and the facile coupling of the resulting primary radical intermediate with the benzylic radical.

The penultimate step, chemoselective oxidation of C18 in **30** to hemiaminal **32**, proved anything but straightforward. The many oxidants examined (e.g., DDQ, Pb­(OAc)_4_, DIAD; as well as several Polonovski reaction protocols on the *N*-oxide of **30**) gave either recovered starting material or a complex mixture of products, which called into question the feasibility of generating the anti-Bredt intermediate **31**. Only upon employing the protocol of Cvengros^24^ did the situation improve, revealing a new, slightly more polar spot by TLC. His method describes the α-acetoxylation of α-C–H bonds in Tröger’s base analogues. In our case, however, no acetate was detected, presumably because it is too labile and undergoes hydrolysis during isolation, likely through the intermediacy of **31**. Finally, desilylation of **32** with TfOH yielded the desired target, ussuriedine (**1**). The spectral data, melting point, and optical rotation of the synthetic sample matched the reported values, confirming that the synthetic journey had reached its target. With the total synthesis complete, the unexpectedly efficient conversion of **28** to **30**—highlighted by the selective [1,2]-Stevens shift—not only secured the target but also raised the intriguing possibility that Nature may employ a Stevens rearrangement in the biosynthesis of ussuriedine. Indeed, the successful use of the [1,2]-Stevens rearrangement in our synthesis may be considered a post facto biomimetic transformation.

### Biosynthesis Hypothesis

Structurally, heilonine and ussuriedine share a striking kinship. Inversion of stereochemistry at C25 and formation of a C–C bond between C18 and C27 would transform heilonine into 18-deoxyussuriedine. This structural similarity, together with their co-occurrence in the same plant,^16^ implies a biogenetic interconnection and offers a basis for a biosynthetic hypothesis (Scheme 6). It is conceivable that heilonine undergoes oxidation at the distal C27 methyl group by a cytochrome P450 enzyme to yield 27-hydroxyheilonine (**33**). The oxidation of aliphatic C–H bonds, including those involving remote methyl groups, by cytochrome P450s is well documented in *Veratrum* alkaloid biosynthesis.^25^ Subsequent activation of the C27 primary alcohol by enzymatic pyrophosphorylation, analogous to the activation that initiates the Wagner–Meerwein rearrangement that transforms the classical steroid framework into the C-*nor*-D-*homo* skeleton (see Scheme 1), would enable quaternization of the piperidine nitrogen to form heiloninium pyrophosphate (**36**).^26^ As previously discussed, given the equatorial orientation of C27 in **35**, cyclization would require a conformational shift to the higher energy aza-cis-decalin conformation (cf. Scheme 2, **9c**), which is plausible through enzyme-induced substrate distortion.^27^ Alternatively, the axial hydroxymethyl isomer may be generated by epimerization of the corresponding aldehyde **34**, followed by reduction.^28^ A subsequent [1,2]-Stevens rearrangement, as validated through our synthetic studies, would then forge the crucial C18–C27 bond to give 18-deoxyussuriedine (**37**). Although Stevens rearrangements have not previously been implicated in biosynthetic pathways, recent reports of enzymatic ylide-mediated rearrangements point to Nature’s capacity for such transformations.^29^ Finally, NAD^+^-mediated oxidation at C18 to form iminium **3**, followed by hydrolysis, would deliver ussuriedine.^30^

**Scheme 6:**
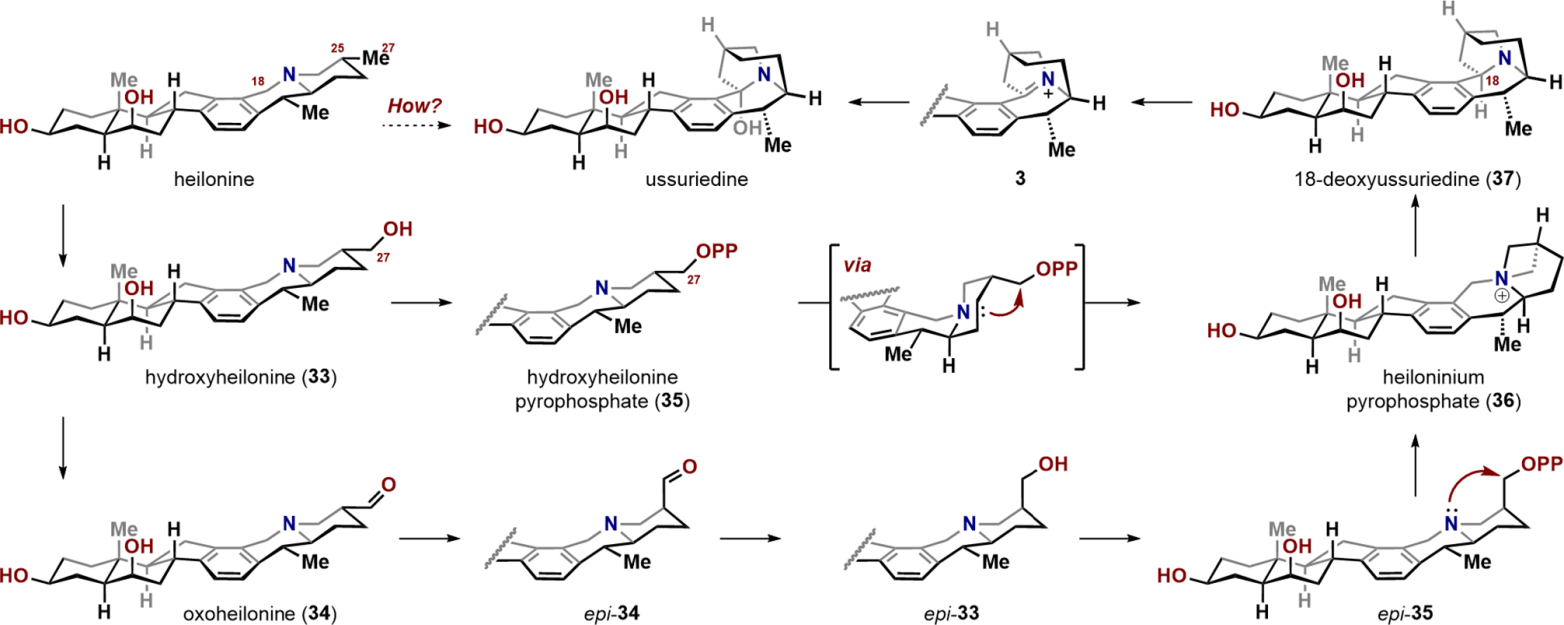
Biosynthetic Hypothesis

Several alternate biosynthetic pathways to the Stevens precursor, heiloninium **36**, are also conceivable. For example, *epi*-**33** could arise via oxidation of 25-*epi*-heilonine. Although this metabolite has not yet been isolated, related cevanine-type alkaloids bearing a β-methyl group at C25 have been reported from the bulbs of *Fritillaria ussuriensis*.^31^ Likewise, it is also possible that oxidation at C27 occurs at an earlier stage in the biosynthetic pathway.^25^ The isolation of additional metabolites from the same plant should help to determine the most likely biosynthetic route to ussuriedine.

## Conclusion

The present study illustrates how total synthesis can transcend its familiar roles of confirming structure or mimicking biosynthesis to serve as a platform for interrogating unconventional strategies and their implications to biosynthetic hypotheses. Inspired by ussuriedine’s structural similarity to its congener heilonine and intrigued by its isolation artifact, we conceived a route that offered a means to probe the possible biogenesis of its unique aza-tricyclic framework. The hexacyclic cevanine skeleton was assembled expeditiously by conjoining two comparably complex fragments and constructing the central benzene ring by a [2 + 2 + 2] cyclotrimerization. The signature cage framework of the natural product was fashioned by a late-stage amine quaternization, Stevens rearrangement, and oxidation sequence, culminating in the first total synthesis of ussuriedine in 23 linear steps from simple materials. The synthesis not only provides access to an intricate natural product but also implicates the involvement of a Stevens rearrangement in natural product biosynthesis.

## Supplementary Material



## Notes

Experimental procedures and characterization data for all new compounds (PDF)
