# Supplementary material for: Total Synthesis of Ussuriedine via a Late-Stage Stevens Rearrangement: Implications for Biosynthesis
Source: J Am Chem Soc. 2026 Apr 3;148(14):15159–65. doi: 10.1021/jacs.6c00926 (PMC13088185; doi:10.1021/jacs.6c00926)
Supplement: Supplementary file 1 [file ja6c00926_si_001.pdf]

Supporting Information

Total Synthesis of Ussuriedine via a Late-Stage Stevens Rearrangement: Implications for Biosynthesis

Daler Baidilov, Kyle J. Cassaidy, Yun-Jeong Shin, and Viresh H. Rawal*

Corresponding author: vrawal@uchicago.edu

Table of Contents

Materials and Methods 2
List of Experimental Procedures 3
Data Matching 30
NMR Spectra 32

Materials and Methods

All reactions were performed in oven-dried (>12 h at 120 °C) and/or flame-dried glassware equipped with a Teflon-coated magnetic stir bar under a nitrogen atmosphere that had been pre-dried by passage through a Drierite® column ($CaSO_4$ ≥ 98% + $CaCl_2$ < 2%), unless otherwise specified. Reaction solvents dichloromethane (CH_2Cl_2; unstabilized HPLC grade), toluene (PhMe; ACS grade), and diethyl ether (Et_2O; ACS grade, stabilized with BHT) were dried by passage through an activated alumina column purification system (Innovative Technology Inc. Pure-Solv™), unless stated otherwise. Tetrahydrofuran (THF; stabilized ACS grade) was freshly distilled from sodium/benzophenone. Anhydrous methanol (MeOH), ethanol (EtOH), and acetonitrile (MeCN) were purchased from Sigma-Aldrich and used as received, unless stated otherwise. Commercially obtained reagents were used as received, unless stated otherwise.

Room temperature refers to 22 °C. Higher temperatures were maintained using preheated oil baths; oil bath temperatures are reported. Lower temperatures were maintained using a cooling bath of acetone/dry ice (−78 °C), water/ice (0 °C), or a NESLAB CB-80 cryobath for all other temperatures in between. Reported temperature values correspond to the cooling bath temperature.

Thin-layer chromatography (TLC) was performed using EMD Millipore silica gel 60 Å plates, and visualization was achieved with either UV fluorescence quenching (254 nm), permanganate stain ($KMnO_4$) with heat, or Seebach's stain (PMA; $Ce(SO_4)_2$ in phosphomolybdic acid) with heat. Flash column chromatography was performed on SiliCycle SiliaFlash P60 (40–63 µm particle size) using ACS grade solvents purchased from Fisher Scientific. Hexanes were freshly distilled prior to use to minimize H-grease content.

Nuclear magnetic resonance (NMR) data were acquired on a 500 MHz Bruker Avance III HD spectrometer equipped with a BBFO SmartProbe, using TopSpin 3.6.2. ^1H NMR spectra were calibrated from residual solvent peaks (e.g., residual $CHCl_3$ in chloroform-d: 7.26 ppm), and $^{13}C\{^1H\}$ NMR spectra from solvent peaks (e.g., chloroform-d: 77.16 ppm). Chemical shifts (δ) are reported in parts per million (ppm) relative to the residual solvent resonance, and coupling constants (J) are reported in hertz (Hz). NMR peak pattern abbreviations are as follows: s = singlet, d = doublet, t = triplet, q = quartet, pent = pentet, sept = septet, dd = doublet of doublets, dt = doublet of triplets, td = triplet of doublets, tt = triplet of triplets, qd = quartet of doublets, ddd = doublet of doublet of doublets, ddt = doublet of doublet of triplets, tdd = triplet of doublet of doublets, m = multiplet, br = broad (i.e., signal is broadened), app = apparent (i.e., signal appears as). All non-trivial ^1H and $^{13}C\{^1H\}$ NMR spectra are corroborated by 2D experiments (e.g., COSY, HSQC, HMBC, NOESY).

High-resolution mass spectrometry (HRMS) analyses were performed on an Agilent Technologies 6224 TOF LC/MS using electrospray ionization (ESI) at the University of Chicago Mass Spectrometry Core Facility. Optical rotations were measured on a Jasco DIP-1000 polarimeter using a 100 mm path-length cell; concentrations are reported as c = g/100 mL. Infrared (IR) spectra were recorded on a Thermo Scientific Nicolet iS50 FT-IR spectrometer and are reported as frequencies of absorption (cm^{-1}). Chiral high-performance liquid chromatography (HPLC) analysis was performed using an Agilent analytical chromatography system with commercial Chiralcel® columns equipped with a guard column. Melting points were recorded on a Thomas Hoover Uni-Melt capillary melting point apparatus and are uncorrected.

List of Experimental Procedures

Preparation of **S2**: Siloxydiene synthesis 4
Preparation of **S5**: Enantioselective Diels-Alder between siloxydiene **S2** and enal **S3** 5
Preparation of **S6**: Isomerization of TBS enol ether **S5** 6
Preparation of **S8**: Seyferth-Gilbert homologation of aldehyde **S6** 7
Preparation of **S9**: 1,4-addition of ammonium enolate of **S8** to MVK 8
Preparation of **S10**: One-pot Robinson annulation/ketalization of diketone **S9** 9
Preparation of **S11**: DIBAL reduction of ester **S10** 10
Preparation of **S12**: Sulfonylation of primary alcohol **S11** 11
Preparation of **12**: Alkynylation of tosylate **S12** with lithium acetylide 12
Preparation of **13**: Methanolysis of acetal **12** 13
Preparation of **14**: C6-hydroxylation of octalone **13** via enolization/epoxidation sequence 14
Preparation of **15**: Isomerization/reduction of γ-hydroxyenone **14** 15
Preparation of **16**: TBS protection of diol **15** 16
Preparation of **17**: DMP oxidation of propargyl alcohol **16** 17
Preparation of **19**: *N*-benzoylation of piperidone **18** 18
Preparation of **21**: Hydroxymethylation of **19** 19
Preparation of **22**: DBU-mediated epimerization of carbinol **21** 20
Preparation of **23**: THP protection of carbinol **22** 21
Preparation of **24**: Hydrazine-mediated *N*-deprotection of THP ether **23** 22
Preparation of **25**: $LiAlH_4$ reduction of secondary lactam **24** 23
Preparation of **26**: Reductive amination of propiolaldehyde **17** with piperidine **25** 24
Preparation of **27**: Rh-catalyzed cyclotrimerization of amine-triyne **26** 25
Preparation of **28**: Deprotection/sulfonylation of hexacyclic acetal **27** 26
Preparation of **30**: Intramolecular *N*-alkylation/Stevens sequence on tosylate **28** 27
Preparation of **32**: Oxidation of the Stevens product **30** 28
Preparation of **1**: TfOH-mediated global deprotection of hemiaminal **32** 29

Preparation of **S2**: Siloxydiene synthesis

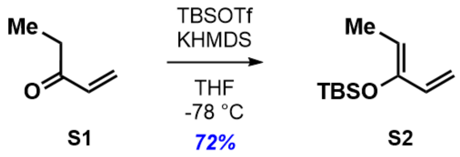

To a pre-cooled solution of ethyl vinyl ketone (**S1**, 11.0 g, 131 mmol, 1.0 eq) in THF (750 mL) was added freshly distilled TBSOTf (30 mL, 131 mmol, 1.0 eq) slowly at −78 °C. The resultant solution was treated with a 1.0 M solution of KHMDS in THF (160 mL, 160 mmol, 1.2 eq), which was added via cannula dropwise over 30 min at –78 °C. Following complete addition, the pale-yellow mixture was stirred at −78 °C for 45 min, then the cold bath was removed, and reaction mixture was stirred for an additional 1 h upon warming to ambient temperature. The mixture was cooled to 0 °C and quenched with a saturated aqueous $NaHCO_3$ solution (300 mL), then concentrated in vacuo until the total volume was approximately 500 mL. The residual mixture was extracted with ether/hexanes = 1:10 (3 x 300 mL), then the combined organic extracts were washed with brine (1 x 400 mL) and dried over Na_2SO_4. Concentration of the dried extracts (high vacuum, 27 °C) provided a crude yellow oil, which was purified via flash column chromatography (pentane) to afford siloxydiene **S2** (18.6 g, 72% yield; >50:1 *Z*:*E*). The material was then further purified by fractional distillation (*ca.* 90% recovery; bp 87 °C at 0.1 atm). Compound **S2** was obtained as a clear, colorless, *odorless* liquid and was confirmed to be free of silanol impurities prior to use.

R_f 0.46 (hexanes), visualized with UV and $KMnO_4$ stain; ^{1}H NMR (500 MHz, $CDCl_3$): δ 6.16 (dd, *J* = 17.1, 10.8 Hz, 1H), 5.27 (d, *J* = 17.1 Hz, 1H), 4.94 (d, *J* = 10.8 Hz, 1H), 4.87 (q, *J* = 7.0 Hz, 1H), 1.64 (d, *J* = 7.0 Hz, 3H), 1.01 (s, 9H), 0.12 (s, 6H); ^{13}C NMR (100 MHz, $CDCl_3$): δ 149.6, 135.7, 111.8, 110.2, 26.1, 18.6, 11.9, -3.5; IR (film, cm^{-1}): 3102 (w), 3024 (w), 2957 (s), 2930 (s), 2859 (s), 1648 (m), 1607 (m), 1474 (m), 1465 (w), 1385 (m), 1364 (w), 1340 (s), 1290 (m), 1255 (s), 1203 (m), 1082 (m), 1050 (s), 1015 (w), 1006 (w), 983 (w), 939 (m), 897 (m), 839 (s), 803 (m), 779 (s), 740 (w), 695 (m).

Preparation of **S5**: Enantioselective Diels-Alder between siloxydiene **S2** and enal **S3**

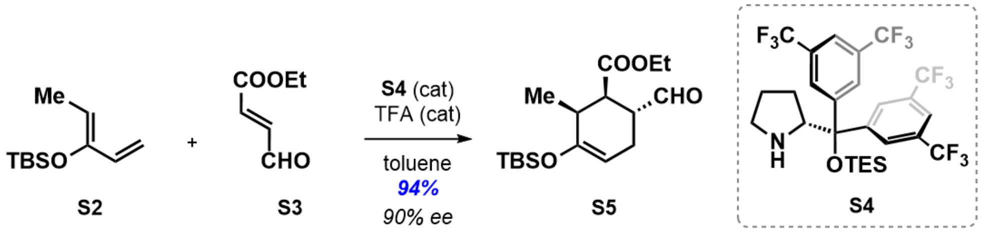

A solution of **S3**[1] (8.6 mL, 71.3 mmol, 1.0 eq) in toluene (freshly distilled from sodium; 4.0 mL) was treated dropwise with solution of diarylprolinol **S4**[2] (4.56 g, 7.13 mmol, 10 mol%) in toluene (11 mL) at ambient temperature. To the pale-yellow solution was added a solution of freshly distilled TFA (0.66 mL, 8.6 mmol, 12 mol%) in toluene (30 mL) and the mixture was cooled to −40 °C. The clear, yellow solution was next treated with siloxydiene **S2** (16.9 g, 85.2 mmol, 1.2 eq) dropwise over 15 min at −40 °C. The resultant solution was stirred at −40 ˚C for 16 h, then quenched with Et_3N (*ca.* 0.15 eq), diluted with hexanes (80 mL), and treated with sat. aq. $NaHCO_3$ (40 mL) at −10 °C. The mixture was brought to ambient temperature and extracted with ether/hexanes = 1:5 (3 x 50 mL), then the combined organic extracts were washed with brine (1 x 100 mL) and dried over Na_2SO_4. Concentration of the dried extracts (bath temp. ≤ 30 °C) afforded a crude, yellow residue that was purified via flash column chromatography (EtOAc/hexanes = 0% → 2% → 4% → 6% → 8%) to afford TBS enol ether **S5** (21.8 g, 94%) as a colorless oil.

HPLC: 90% *ee*, R_t (major) = 21.5 min, R_t (minor) = 21.9 min; CHIRALPAK® OD-H column: *i*-PrOH/hexanes = 0.2% → 1%; 0.5 mL/min, λ = 210 nm.

Peak #	RetTime [min]	Type	Width [min]	Area [mAU*s]	Height [mAU]	Area %
1	21.343	MM	0.1615	8709.62891	898.64270	49.9636
2	21.874	MM	0.2107	8722.31152	689.80365	50.0364

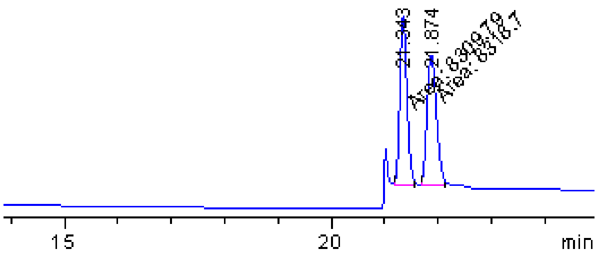

Peak #	RetTime [min]	Type	Width [min]	Area [mAU*s]	Height [mAU]	Area %
1	21.539	MM	0.1304	1.35130e4	1727.70215	94.7189
2	21.942	MM	0.1897	753.42407	66.20831	5.2811

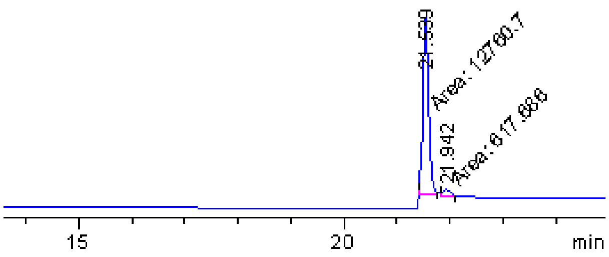

R_f 0.32 (EtOAc/hexanes = 1:10), visualized with $KMnO_4$ stain; $[\alpha]_D^{22}$ = −105° (*c* = 1.0, $CHCl_3$); ^{1}H NMR (500 MHz, $CDCl_3$): δ 9.81 (d, *J* = 1.8 Hz, 1H), 4.75 (dd, *J* = 5.3, 2.4 Hz, 1H), 4.18 (q, *J* = 7.1 Hz, 2H), 3.01 (dd, *J* = 11.8, 5.6 Hz, 1H), 2.92 (tdd, *J* = 11.6, 5.7, 1.8 Hz, 1H), 2.54 (app pent, *J* = 6.6 Hz, 1H), 2.30 (dt, *J* = 16.7, 5.5 Hz, 1H), 1.98 (app ddt, *J* = 16.7, 11.4, 2.0 Hz, 1H), 1.27 (t, *J* = 7.1 Hz, 3H), 1.00 (d, *J* = 7.0 Hz, 3H), 0.93 (s, 9H), 0.15 (6H); ^{13}C NMR (100 MHz, $CDCl_3$): δ 203.1, 172.9, 153.6, 99.3, 60.9, 45.3, 42.6, 35.2, 25.8, 24.0, 18.1, 15.0, 14.4, -4.2, -4.5; IR (film, cm^{-1}): 2956 (m), 2929 (m), 2856 (m), 1727 (s), 1669 (s), 1472 (m), 1463 (m), 1377 (m), 1302 (m), 1278 (m), 1251 (m), 1196 (s), 1097 (w), 1027 (m), 838 (s), 778 (s), 665 (w); HRMS (ESI): m/z $[M+H]^+$ calcd for 327.1991, found 327.1994.

[1] This material (commercially available, Alfa Aesar) had a tendency to decompose upon storing for several months in a refrigerator. The purity was always checked via ^{1}H NMR prior to use and, if necessary, purified via flash column chromatography, see: *Org. Synth.* **2018**, *95*, 142−156.

[2] Lin, Q.; Meloni, D.; Pan, Y.; Xia, M.; Rodgers, J.; Shepard, S.; Li, M.; Galya, L.; Metcalf, B.; Yue, T.; Liu, P.; Zhou, J. Enantioselective Synthesis of Janus Kinase Inhibitor INCB018424 via an Organocatalytic Aza-Michael Reaction. *Org. Lett.* **2009**, *11*, 1999−2002.

Preparation of **S6**: Isomerization of TBS enol ether **S5**

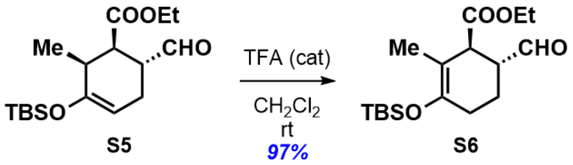

To a solution of **S5** (18.6 g, 57.1 mmol, 1 eq) in CH_2Cl_2 (distilled from CaH_2, 175 mL) was added freshly distilled TFA (0.22 mL, 2.85 mmol, 5 mol%) dropwise at ambient temperature. The resultant mixture was stirred for 24 hours and, following complete consumption of starting material as indicated by TLC, neutralized with Et_3N (0.80 mL, 5.7 mmol, 0.1 eq) dropwise. Concentration of the neutralized solution provided a crude residue, which was purified via flash column chromatography (EtOAc/hexanes =1:25) to afford isomerized TBS enol ether **S6** (18.1 g, 97%) as a colorless oil.

R_f 0.4 (EtOAc/hexanes = 1:9), visualized with UV and $KMnO_4$ stain; $[\alpha]_D^{20}$ = −37° (*c* = 0.2, $CHCl_3$); ^{1}H NMR (600 MHz, $CDCl_3$) δ 9.62 (s, 1H), 4.17 (q, *J* = 7.1 Hz, 2H), 3.40 – 3.36 (m, 1H), 2.81 (dt, *J* = 7.2, 4.3 Hz, 1H), 2.14 – 2.03 (m, 3H), 1.94 (dt, *J* = 11.1, 6.3 Hz, 1H), 1.66 (dt, *J* = 1.9, 1.0 Hz, 3H), 1.27 (t, *J* = 7.1 Hz, 3H), 0.93 (s, 9H), 0.10 (s, 3H), 0.10 (s, 3H); ^{13}C NMR (150 MHz, $CDCl_3$) δ 202.0, 173.8, 146.2, 108.1, 61.1, 48.8, 45.8, 27.9, 25.9, 20.7, 18.3, 15.5, 14.4, -3.7, -3.8; HRMS (ESI): m/z $[M]^+$ calcd for 326.1913, found 326.1922.

Preparation of **S8**: Seyferth-Gilbert homologation of aldehyde **S6**

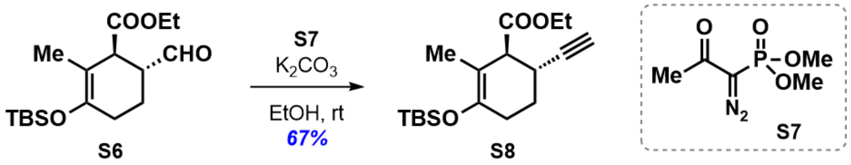

To a solution of aldehyde **S6** (19.6 g, 60.1 mmol, 1 eq) and Bestmann-Ohira reagent **S7** (12.7 g, 66.1 mmol, 1.1 eq) in freshly distilled EtOH (300 mL) was added K_2CO_3 (13.3 g, 96.2 mmol, 1.6 eq) at ambient temperature. After stirring for 96 h, the mixture was diluted with hexanes (300 mL) and quenched with sat. aq. NH_4Cl. The biphasic mixture was separated, and the aqueous layer was extracted with ether/hexanes = 1:5 (3 x 150 mL). The combined organic extract was washed with brine (1 x 300mL) and dried over Na_2SO_4. The dried extract was concentrated under reduced pressure, and the resultant crude residue was purified via careful gradient column chromatography (EtOAc/hexanes = 1:100 → 1:25) to afford alkyne **S8** (11.3 g, 67%) as a colorless oil.

R_f 0.43 (EtOAc/hexanes = 1:19), visualized with $KMnO_4$ stain; $[\alpha]_D^{20}$ = −75° (*c* = 0.3, $CHCl_3$); ^{1}H NMR (500 MHz, $CDCl_3$) δ 4.17 (qd, *J* = 7.1, 1.4 Hz, 2H), 3.11 (d, J = 6.2 Hz, 1H), 2.93 (dddd, *J* = 8.3, 5.9, 3.3, 2.4 Hz, 1H), 2.27 – 2.17 (m, 1H), 2.14 – 2.06 (m, 1H), 2.05 (d, *J* = 2.4 Hz, 1H), 2.01 (dddd, *J* = 12.9, 6.9, 5.9, 3.4 Hz, 1H), 1.73 (ddt, *J* = 13.0, 8.1, 6.3 Hz, 1H), 1.60 (td, *J* = 1.9, 0.9 Hz, 3H), 1.26 (t, *J* = 7.1 Hz, 3H), 0.94 (s, 9H), 0.13 (s, 3H), 0.13 (s, 3H); ^{13}C NMR (125 MHz, $CDCl_3$) δ 173.6, 145.9, 107.8, 85.7, 69.2, 60.9, 52.2, 28.9, 28.3, 26.6, 26.0, 18.4, 15.1, 14.4, -3.6, -3.8; HRMS (ESI): m/z $[M]^+$ calcd for 322.1964, found 322.1962.

Preparation of **S9**: 1,4-addition of ammonium enolate of **S8** to MVK

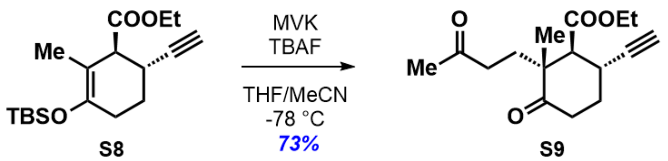

To a pre-cooled solution of TBAF (1.0 M solution in THF, 13.7 mL, 13.7 mmol, 1 eq) in MeCN/THF = 1:2 (111 mL) was slowly added a solution of **S8** (4.41 g, 13.7 mmol, 1 eq) and MVK (freshly distilled from Na_2CO_3; 1.37 mL, 1.2 eq) in MeCN/THF = 1:2 (16 mL) over 2 h at -78°C. After complete addition, the resultant solution was stirred for additional 15 min at the same temperature. The mixture was quenched with H_2O, warmed up to ambient temperature, and extracted with CH_2Cl_2 (3 x 70 mL). The combined organic extract was washed with brine (1 x 70 mL) and dried over Na_2SO_4. Concentration of the dried extract provided a crude yellow residue, which was purified via flash column chromatography (EtOAc/hexanes = 1:10 → 1:4) to afford diketone **S9** (dr > 50:1, 2.84 g, 73%) as a colorless oil.[3]

R_f 0.27 (EtOAc/hexanes = 1:4), visualized with $KMnO_4$ stain; $[\alpha]_D^{20}$ = +5° (*c* = 0.5, $CHCl_3$); ^{1}H NMR (500 MHz, $CDCl_3$) δ 4.20 (q, *J* = 7.2 Hz, 2H), 3.23 (tdd, *J* = 11.7, 3.9, 2.3 Hz, 1H), 2.77 (d, *J* = 11.2 Hz, 1H), 2.59 (tdd, *J* = 15.0, 7.7, 5.8 Hz, 2H), 2.46 (ddd, *J* = 16.7, 11.2, 4.5 Hz, 1H), 2.37 – 2.27 (m, 1H), 2.14 (s, 3H), 2.10 (dd, *J* = 2.4, 0.7 Hz, 1H), 2.05 (ddd, *J* = 14.4, 11.1, 4.4 Hz, 1H), 1.85 – 1.72 (m, 1H), 1.53 (ddd, *J* = 14.4, 11.2, 4.7 Hz, 1H), 1.26 (td, *J* = 7.2, 0.7 Hz, 3H), 1.18 (s, 3H); ^{13}C NMR (125 MHz, $CDCl_3$) δ 210.6, 208.1, 170.5, 84.4, 70.5, 61.1, 54.6, 49.3, 38.6, 36.8, 30.7, 30.1, 30.1, 27.7, 21.7, 14.4; HRMS (ESI): m/z $[M]^+$ calcd for 278.1518, found 278.1521.

[3] The corresponding desilylated product was also obtained as a 1:1 mixture of diastereomers (0.57 g, 20%), which was conveniently recycled (TMSI, HMDS, CH_2Cl_2, 0 °C).

Preparation of **S10**: One-pot Robinson annulation/ketalization of diketone **S9**

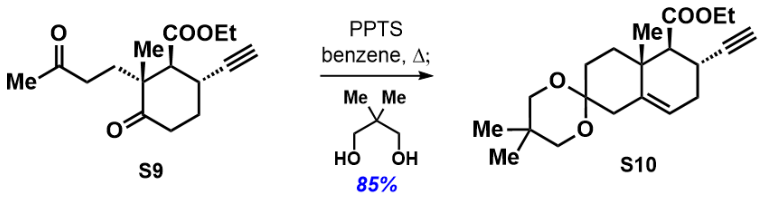

A 1 L round bottom flask was charged with diketone **S9** (4 g, 14.3 mmol, 1 eq) and PPTS (7.2 g, 28.7 mmol, 2 eq). Then a Dean-Stark apparatus equipped with a reflux condenser was attached and the system was flushed with N_2. Benzene (0.05 M, 286 mL) was then added, and the reaction mixture was placed in oil bath pre-heated to 110 °C. After refluxing for 96 h, the mixture was cooled down to room temperature and neopentyl glycol (7.4 g, 71.5 mmol, 5 eq) was added. After refluxing 18 h, the mixture was once again cooled to ambient temperature and quenched with sat. aq. $NaHCO_3$. The layers were separated, and the aqueous phase was extracted with EtOAc/hexanes = 1:1 twice. The combined organic extract was washed with brine, dried over Na_2SO_4, filtered, and evaporated providing crude material, which was purified using silica gel column chromatography (EtOAc/hexanes = 1:19 → 1:19 → 1:6) furnishing cyclic ketal **S10** (4.21 g, 85%) as a white solid.

R_f 0.29 (EtOAc/hexanes = 1:9), visualized with $KMnO_4$ stain; $[\alpha]_D^{20}$ = +87° (*c* = 0.4, $CHCl_3$); mp (MeOH) 101-102 °C; ^{1}H NMR (600 MHz, $CDCl_3$) δ 5.32 (dt, *J* = 4.8, 2.2 Hz, 1H), 4.20 (q, *J* = 7.1 Hz, 2H), 3.58 (d, *J* = 11.3 Hz, 1H), 3.49 – 3.40 (m, 3H), 2.89 (tdd, *J* = 11.7, 5.8, 2.3 Hz, 1H), 2.56 (dd, *J* = 14.4, 3.2 Hz, 1H), 2.48 (d, *J* = 12.0 Hz, 1H), 2.37 (dtd, *J* = 17.9, 5.5, 2.9 Hz, 1H), 2.30 (dddd, *J* = 17.3, 9.2, 4.6, 2.6 Hz, 2H), 2.20 (dddd, *J* = 17.6, 11.4, 3.5, 2.2 Hz, 1H), 2.02 (d, J = 2.2 Hz, 1H), 1.68 – 1.59 (m, 1H), 1.58 – 1.48 (m, 2H), 1.28 (t, *J* = 7.1 Hz, 3H), 1.10 (s, 3H), 1.01 (s, 3H), 0.89 (s, 3H); ^{13}C NMR (150 MHz, $CDCl_3$) δ 172.6, 138.5, 120.6, 98.0, 86.4, 70.4, 70.1, 69.2, 60.4, 57.7, 40.1, 37.4, 35.5, 31.9, 30.2, 27.0, 25.3, 22.9, 22.7, 20.0, 14.5; HRMS (ESI): m/z $[M]^+$ calcd for 346.2144, found 346.2144.

Preparation of **S11**: DIBAL reduction of ester **S10**

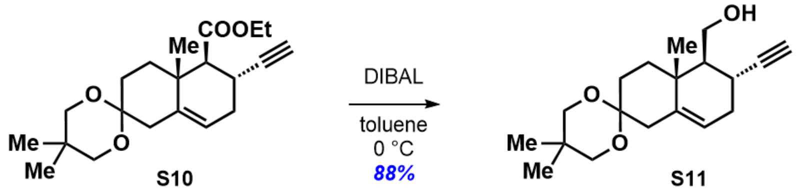

To a stirred solution of ethyl ester **S10** (3.3 g, 9.52 mmol, 1 eq) in toluene (0.2 M, 48 mL) was cannulated a freshly cracked bottle of DIBAL (1.0 M solution in toluene, 25 mL, 2.6 eq) at 0 °C over 20 min. After complete addition, the reaction mixture was allowed to stir for 5 min, then quenched with sat. aq. Rochelle salt. A cooling bath was removed, and the mixture was diluted with EtOAc and water. The resulting slurry was stirred at room temperature until clear biphasic mixture formed (*ca.* 1.5 h). Then, the layers were separated, and the aqueous layer was extracted with EtOAc four times. The combined organic extract was washed with brine, dried over Na_2SO_4, filtered, and evaporated providing white solid, which was purified using silica gel column chromatography (wet-load [CH_2Cl_2], EtOAc/hexanes = 1:9 → 1:4 → 3:7) to furnish alcohol **S11** (2.55 g, 88%) as a white solid.

R_f 0.64 (EtOAc/hexanes = 1:1), visualized with PMA stain; $[\alpha]_D^{23.0}$ = −37° (*c* = 1.0, $CHCl_3$); ^{1}H NMR (400 MHz, $CDCl_3$): δ 5.35 (m, 1H), 3.93 – 3.85 (m, 1H), 3.85 – 3.78 (m, 1H), 3.58 (d, *J* = 11.3 Hz, 1H), 3.48 (d, *J* = 11.3 Hz, 1H), 3.45 (s, 2H), 2.69 – 2.59 (tdd, *J* = 11.2, 5.4, 2.5 Hz, 1H), 2.58 – 2.49 (m, 2H), 2.38 – 2.19 (m, 4H), 2.23 (d, *J* = 2.4 Hz), 1.87 (dt, *J* = 13.0, 3.5 Hz, 1H), 1.60 – 1.50 (m, 2H), 1.43 – 1.33 (td, *J* = 13.6, 3.7 Hz, 1H), 1.01 (s, 3H), 0.99 (s, 3H) 0.91 (s, 3H); ^{13}C NMR (100 MHz, $CDCl_3$): δ 139.6, 120.9, 98.2, 87.9, 70.9, 70.4, 70.0, 63.0, 52.6, 39.8, 37.7, 34.5, 33.1, 30.2, 27.2, 26.5, 22.9, 22.7, 18.5; IR (film, cm^{-1}): 3461 (br), 3301 (s), 2951 (s), 2866 (m), 2110 (w), 1671 (w), 1471 (m), 1394 (m), 1363 (s), 1269 (m), 1097 (s), 1058 (w), 1030 (s), 975 (m), 948 (w), 907 (w), 801 (w), 735 (s), 702 (m), 630 (s); HRMS (ESI): m/z $[M+H]^+$ calcd for 305.2116, found 305.2119.

Preparation of **S12**: Sulfonylation of primary alcohol **S11**

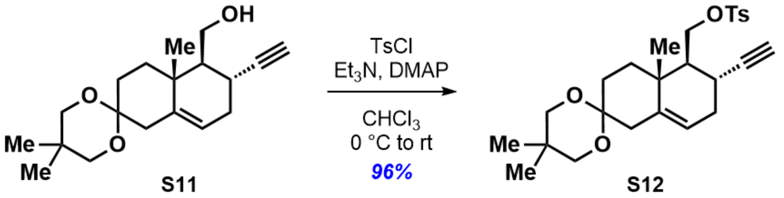

To a pre-cooled solution of primary alcohol **S11** (0.729 g, 2.39 mmol, 1 eq) in $CHCl_3$ (35 mL) were added TsCl (1.37 g, 7.17 mmol, 3 eq), Et_3N (3.3 mL, 23.7 mmol, 10 eq), and DMAP (0.584 g, 4.78 mmol, 2 eq) sequentially at 0 °C. The resultant mixture was brought to ambient temperature and stirred for 24 h. Upon consumption of starting material as indicated by TLC, the reaction mixture was treated with sat. aq. $NaHCO_3$ (25 mL), diluted with H_2O (25 mL), and stirred vigorously for 30 min to hydrolyze unreacted TsCl. The mixture was then extracted with $CHCl_3$ (5 x 40 mL), and the combined organic extract was dried over Na_2SO_4. Concentration of the dried extract provided a crude yellow oil, which was purified via flash column chromatography (hexanes/EtOAc/CH_2Cl_2 = 20:2:1 → 20:3:2) to afford tosylate **S12** (1.05 g, 96%) as a white solid.

R_f 0.45 (EtOAc/hexanes = 1:3), visualized with UV and PMA stain; $[\alpha]_D^{22}$ = −60° (*c* = 1.0, $CHCl_3$); ^1H NMR (400 MHz, $CDCl_3$): δ 7.82 (d, *J* = 8.3 Hz, 2H), 7.34 (d, *J* = 8.3 Hz, 2H), 5.31 (m, 1H), 4.48 (dd, *J* = 10.1, 2.3 Hz, 1H), 4.28 (dd, *J* = 10.1, 5.7 Hz, 1H), 3.57 (d, *J* = 11.2 Hz, 1H), 3.47 (d, *J* = 11.2 Hz, 1H), 3.42 (s, 2H), 2.64 – 2.56 (tdd, *J* = 11.4, 5.5, 2.3 Hz, 1H), 2.56 – 2.50 (dd, *J* = 14.2, 3.1 Hz, 1H), 2.45 (s, 3H), 2.39 – 2.13 (m, 4H), 1.86 (d, *J* = 2.3 Hz, 1H), 1.83 – 1.76 (dt, *J* = 13.0, 3.5 Hz, 1H), 1.68 – 1.62 (ddd, *J* = 11.6, 5.7, 2.4 Hz, 1H), 1.59 – 1.49 (td, *J* = 14.1, 3.8 Hz, 1H), 1.34 – 1.24 (td, *J* = 13.6, 3.8 Hz, 1H), 1.05 (s, 3H), 1.00 (s, 3H), 0.90 (s, 3H); ^{13}C NMR (101 MHz, $CDCl_3$): δ 144.8, 139.2, 133.0, 130.0, 128.4, 120.9, 98.0, 85.4, 70.8, 70.4, 70.1, 69.0, 48.6, 39.9, 37.7, 35.4, 33.0, 30.2, 27.2, 25.1, 22.9, 22.7, 21.8, 19.3; IR (film, cm^{-1}): 3276 (s), 2952 (s), 2867 (m), 1598 (m), 1467 (m), 1361 (s), 1269 (w), 1189 (m), 1176 (s), 1104 (s), 1022 (m), 941 (m), 857 (m), 814 (m), 711 (w), 664 (s), 555 (s); HRMS (ESI): m/z $[M+H]^+$ calcd for 459.2205, found 459.2205.

Preparation of **12**: Alkynylation of tosylate **S12** with lithium acetylide

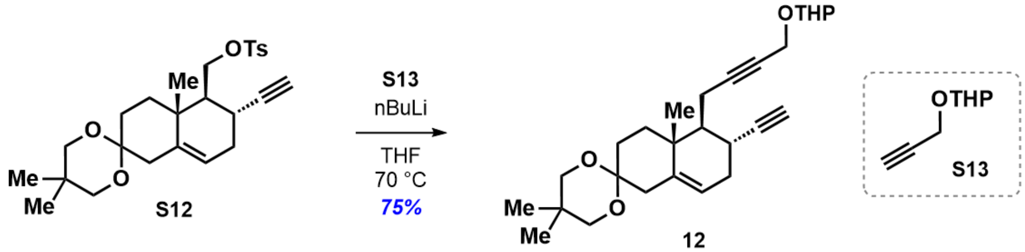

To a solution of tetrahydro-2-(2-propynyloxy)-2*H*-pyran (**S13**, purified via short-path distillation under reduced pressure prior to use; 0.80 mL, 5.66 mmol, 2.5 eq) in dry THF (16 mL) was added nBuLi (1.60 M in hexanes; 3.3 mL, 5.28 mmol, 2.4 eq) dropwise at −78 °C. The cold bath was removed, and the mixture was allowed to warm to ambient temperature, then the pale-yellow solution was stirred at room temperature for 2 h. After re-cooling to −78 °C, the acetylide was treated dropwise with a solution of tosylate **S12** (azeotroped with benzene; 1.01 g, 2.21 mmol, 1 eq) in dry THF (7.5 mL). Following complete addition, the cooling bath was removed, and the mixture was allowed to warm to ambient temperature and stirred for an additional 30 min before heating to 70 °C in a pre-heated oil bath. The reaction mixture was stirred at this temperature for 18 h, then cooled to ambient temperature, quenched with sat. aq. $NaHCO_3$ (40 mL) and extracted with EtOAc/hexanes = 1:1 (3 x 40 mL). The combined organic extract was washed with brine (1 x 100 mL), dried over Na_2SO_4, then concentrated in vacuo. The resulting crude product, a light-brown oil, was purified via flash column chromatography (EtOAc/hexanes = 1:18 → 1:9) to afford diyne **12** (0.708 g, 75%) as a white, amorphous solid.

R_f 0.46 (EtOAc/hexanes = 1:4), visualized with $KMnO_4$ stain; $[\alpha]_D^{22}$ = −45° (*c* = 1.0, $CHCl_3$); ^{1}H NMR (400 MHz, $CDCl_3$): δ 5.33 (m, 1H), 4.85 (m, 1H), 4.24 (m, 2H), 3.89 – 3.80 (m, 1H), 3.57 (d, *J* = 11.2 Hz, 1H), 3.47 (d, *J* = 11.2 Hz, 1H), 3.56 – 3.48 (m, 1H), 3.45 (s, 1H), 3.44 (s, 1H), 2.72 – 2.52 (m, 4H), 2.40 – 2.17 (m, 4H), 2.11 (d, *J* = 2.2 Hz, 1H), 2.00 – 1.92 (dt, *J* = 13.1, 3.1 Hz, 1H), 1.88 – 1.78 (m, 1H), 1.78 – 1.68 (m, 1H), 1.68 – 1.46 (m, 6H), 1.38 – 1.27 (td, *J* = 13.7, 3.7 Hz, 1H), 1.10 (s, 3H), 1.00 (s, 3H), 0.91 (s, 3H); ^{13}C NMR (101 MHz, $CDCl_3$): δ 139.5, 120.9, 98.2, 96.5, 87.1, 86.4, 77.3, 70.4, 70.0, 62.3, 62.2, 54.7, 48.0, 39.9, 38.6, 35.3, 33.2, 30.5, 30.2, 27.6, 27.5, 25.6, 22.9, 22.7, 19.4, 18.9, 18.3; IR (film, cm^{-1}): 3303 (s), 2948 (s), 2867 (m), 2113 (w), 1673 (w), 1453 (m), 1394 (m), 1362 (m), 1266 (m), 1200 (m), 1077 (s), 1104 (w), 1020 (s), 968 (m), 902 (m), 869 (m), 811 (m), 735 (s), 702 (w), 633 (m); HRMS (ESI): m/z $[M+H]^+$ calcd for 427.2848, found 427.2847.

Preparation of **13**: Methanolysis of acetal **12**

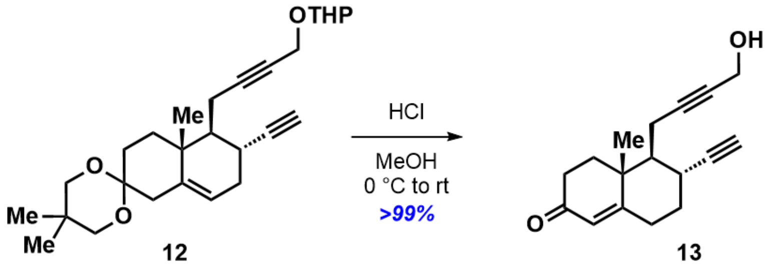

To a stirred solution of cyclic ketal **12** (1.76 g, 4.12 mmol, 1 eq) in MeOH (0.05 M, 82 mL) was added aq. HCl (6.0 M, 13.7 mL, 82.5 mmol, 20 eq) dropwise over 10 min at 0 °C using an addition funnel. After complete addition, the reaction mixture was stirred at 0 °C for 3 h, then quenched with sat. aq. $NaHCO_3$ and allowed to warm to room temperature. The mixture was extracted with EtOAc four times, and the combined organic layers were washed with brine, dried over Na_2SO_4, filtered, and concentrated under reduced pressure. The crude material was purified by silica gel column chromatography (acetone/CH_2Cl_2 = 1:19 → 1:4) to afford octalone **13** (1.05 g, >99%) as a white solid.

R_f 0.55 (EtOAc/hexanes = 1:2), visualized with UV and $KMnO_4$ stain; $[\alpha]_D^{25}$ = +93° (*c* = 1.0, $CHCl_3$); mp (EtOAc/hexanes) 91-92 °C; ^1H NMR (500 MHz, $CDCl_3$) δ 5.74 (d, *J* = 1.8 Hz, 1H), 4.26 (dd, *J* = 2.2, 2.2 Hz, 2H), 2.80 (tdd, *J* = 12.0, 3.7, 2.3 Hz, 1H), 2.73 (ddt, *J* = 17.7, 6.5, 2.3 Hz, 1H), 2.63 (dq, *J* = 17.7, 2.4 Hz, 1H), 2.50 – 2.35 (m, 3H), 2.31 – 2.16 (m, 3H), 2.14 (d, *J* = 2.3 Hz, 1H), 1.85 (td, *J* = 13.4, 5.3 Hz, 1H), 1.67 – 1.53 (m, 3H), 1.30 (s, 3H); ^{13}C NMR (125 MHz, $CDCl_3$) δ 199.0, 168.1, 124.6, 86.0, 85.0, 80.5, 70.5, 51.6, 50.3, 39.8, 36.1, 33.8, 33.0, 32.2, 30.3, 18.4, 18.0; IR (film, cm^{-1}): 3406 (br), 3284 (s), 2937 (s), 2865 (m), 2232 (w), 1660 (s), 1618 (m), 1430 (m), 1351 (m), 1274 (w), 1232 (m), 1187 (w), 1140 (w), 1020 (s), 948 (m), 868 (w), 647 (s); HRMS (ESI): m/z $[M]^+$ calcd for 256.1463, found 256.1466.

Preparation of **14**: C6-hydroxylation of octalone **13** via enolization/epoxidation sequence

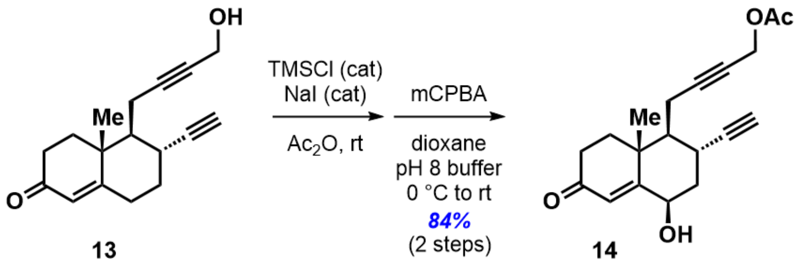

To a stirred solution of octalone **13** (0.84 g, 3.28 mmol, 1 eq) in Ac_2O (0.25 M, 13.1 mL) were sequentially added NaI (50 mg, 0.33 mmol, 0.1 eq) and TMSCl (0.05 mL, 0.39 mmol, 0.12 eq) at room temperature. After stirring for 7 h, additional NaI and TMSCl (same amount) were added, and the mixture was allowed to stir for 3 more hours. Then, after full consumption of starting material as indicated by TLC, the reaction mixture was quenched with pyridine (0.26 mL, 3.28 mmol, 1 eq) and evaporated to dryness (high vacuum, 40 °C). The residue was re-dissolved in EtOAc/hexanes = 1:1 washed with sat. aq. $Na_2S_2O_3$, sat. aq. $NaHCO_3$, water, and brine. The organic phase was dried over Na_2SO_4, filtered, and evaporated providing crude material, which was used in the next step without further purification.

To a stirred solution of crude dienol acetate in 1,4-dioxane (0.1 M, 33 mL) were sequentially added aq. phosphate buffer (pH 8, 0.1 M, 33 mL) and mCPBA (77% w/w H_2O, 0.81 g, 3.61 mmol, 1.1 eq) at 0 °C. The reaction mixture was brought to ambient temperature slowly overnight. After stirring for 18 h in total, the mixture was treated with sat. aq. $Na_2S_2O_3$ and sat. aq. $NaHCO_3$. The mixture was then stirred vigorously for 15 min and diluted with EtOAc. The layers were separated, and the aqueous phase was extracted with EtOAc three times. The combined organic extract was dried over Na_2SO_4, filtered, and evaporated providing crude material, which was purified using silica gel column chromatography (acetone/hexanes = 20% → 25% → 30% → 40%) to furnish γ-hydroxyenone **14** (dr = 5:1, 0.86 g, 84%) as a white solid. The characterization data is provided for the major β-epimer.

R_f 0.34 (acetone/hexanes = 1:2), visualized with UV and $KMnO_4$ stain; $[\alpha]_D^{22}$ = +62° (*c* = 0.8, $CHCl_3$); mp (EtOAc/hexanes) 111-113 °C; ^{1}H NMR (500 MHz, $CDCl_3$) δ 5.85 (d, *J* = 1.0 Hz, 1H), 4.68 (t, *J* = 2.3 Hz, 2H), 4.38 (t, *J* = 3.0 Hz, 1H), 3.22 (tt, *J* = 12.2, 3.0 Hz, 1H), 2.72 (dq, *J* = 6.2, 2.2 Hz, 2H), 2.57 (ddd, *J* = 17.4, 14.8, 5.0 Hz, 1H), 2.45 (dddd, *J* = 17.4, 4.1, 2.9, 1.0 Hz, 1H), 2.38 (dt, *J* = 14.3, 3.2 Hz, 1H), 2.28 (ddd, *J* = 13.3, 5.0, 2.9 Hz, 1H), 2.15 (d, *J* = 2.4 Hz, 1H), 2.10 (s, 3H), 1.89 – 1.81 (m, 2H), 1.59 (ddd, *J* = 11.7, 5.9, 3.7 Hz, 1H), 1.48 (s, 3H); ^{13}C NMR (125 MHz, $CDCl_3$) δ 199.7, 170.5, 165.7, 127.0, 86.2, 86.0, 76.2, 72.4, 70.6, 52.9, 50.5, 39.3, 39.2, 37.7, 34.1, 25.4, 20.9, 20.3, 18.1; HRMS (ESI): m/z $[M]^+$ calcd for 314.1518, found 314.1522.

Preparation of **15**: Isomerization/reduction of γ-hydroxyenone **14**

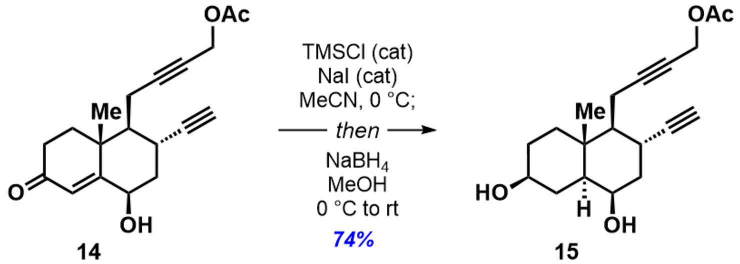

14 15

To a stirred solution of γ-hydroxyenone **14** (0.76 g, 2.42 mmol, 1 eq) in MeCN (0.1 M, 24 mL) were sequentially added NaI (36 mg, 0.24 mmol, 0.1 eq) and TMSCl (0.061 mL, 0.48 mmol, 0.2 eq) at 0 °C. After stirring at 0 °C for 1 h and 20 min, clean formation of the intermediate diketone was observed on TLC, and the mixture was neutralized with Et_3N (0.33 mL, 2.42 mmol, 1 eq). After stirring for 10 min, the reaction mixture was diluted with MeOH (24 mL) and $NaBH_4$ (0.37 g, 9.68 mmol, 4 eq) was added. The reaction mixture was allowed to stir for 50 more minutes at 0 °C, then dry-loaded on silica gel. Elution (acetone/hexanes = 30% → 40% → 50% → 60%) provided diol **15** (0.57 g, 74%) as a white solid.

R_f 0.44 (acetone/hexanes = 1:1), visualized with $KMnO_4$ stain; $[\alpha]_D^{23}$ = +15° (*c* = 0.45, $CHCl_3$); mp (acetone) 87-90 °C; ^1H NMR (500 MHz, $CDCl_3$) δ 4.64 (t, *J* = 2.3 Hz, 2H), 3.80 (dd, *J* = 2.9, 2.9 Hz, 1H), 3.65 (ddd, *J* = 10.6, 10.6, 4.9 Hz, 1H), 2.97 – 2.87 (m, 1H), 2.66 – 2.51 (m, 2H), 2.18 – 2.13 (m, 2H), 2.08 (d, *J* = 2.4 Hz, 1H), 2.07 (s, 3H), 1.84 (ddq, *J* = 10.5, 5.4, 2.7 Hz, 2H), 1.78 – 1.67 (m, 2H), 1.67 – 1.52 (m, 1H), 1.52 – 1.43 (m, 1H), 1.34 (ddd, *J* = 11.6, 6.4, 3.2 Hz, 1H), 1.21 (dt, *J* = 12.7, 2.9 Hz, 1H), 1.13 – 1.08 (m, 1H), 1.07 (s, 3H); ^{13}C NMR (125 MHz, $CDCl_3$) δ 170.6, 87.5, 87.3, 75.3, 71.3, 71.0, 69.9, 53.1, 51.7, 46.8, 41.1, 38.9, 37.0, 35.1, 31.3, 26.2, 21.0, 18.0, 15.6; HRMS (ESI): m/z $[M]^+$ calcd for 318.1831, found 318.1838.

Preparation of **16**: TBS protection of diol **15**

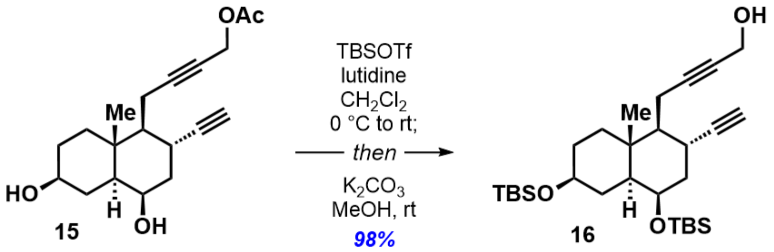

To a stirred solution of diol **15** (0.695 g, 2.18 mmol, 1 eq) in CH_2Cl_2 (0.1 M, 22 mL) was added 2,6-lutidine (0.76 mL, 6.54 mmol, 3 eq) at room temperature. The mixture was cooled to 0 °C and treated dropwise with TBSOTf (1.3 mL, 5.67 mmol, 2.6 eq). The reaction mixture was slowly warmed to room temperature overnight. After stirring for 12 h in total, the reaction mixture was diluted with MeOH (22 mL) and treated with K_2CO_3 (1.2 g, 8.72 mmol, 4 eq). After stirring at room temperature for 6 more hours, the reaction mixture was quenched with sat. aq. NH_4Cl and extracted with CH_2Cl_2 three times. The combined organic extract was dried over Na_2SO_4, filtered, and evaporated providing crude material, which was purified using silica gel column chromatography (EtOAc/hexanes = 5% → 10% → 15%) to furnish propargyl alcohol **16** (1.08 g, 98%) as a white solid.

R_f 0.49 (EtOAc/hexanes = 1:4), visualized with $KMnO_4$ stain; $[\alpha]_D^{20}$ = –81° (*c* = 0.8, $CHCl_3$); ^1H NMR (500 MHz, $CDCl_3$) δ 4.23 (ddd, *J* = 6.1, 2.2, 2.2 Hz, 2H), 3.70 (dd, *J* = 2.7, 2.7 Hz, 1H), 3.62 – 3.53 (m, 1H), 2.89 – 2.80 (m, 1H), 2.58 (ddd, *J* = 17.6, 2.6, 2.5 Hz, 1H), 2.51 (dddd, *J* = 17.6, 6.6, 2.3, 2.3 Hz, 1H), 2.08 (d, *J* = 2.3 Hz, 1H), 2.04 – 1.98 (m, 1H), 1.80 (dt, *J* = 13.3, 3.6 Hz, 1H), 1.73 – 1.63 (m, 3H), 1.53 – 1.36 (m, 3H), 1.31 (ddd, *J* = 11.5, 6.6, 3.1 Hz, 1H), 1.10 (dt, *J* = 13.0, 2.6 Hz, 1H), 1.09 – 0.99 (m, 1H), 1.03 (s, 3H), 0.89 (s, 9H), 0.88 (s, 9H), 0.05 (s, 6H), 0.03 (s, 3H), 0.02 (s, 3H); ^{13}C NMR (125 MHz, $CDCl_3$) δ 88.0, 87.0, 79.3, 72.4, 71.5, 69.6, 52.3, 51.7, 47.5, 41.9, 39.3, 37.3, 36.5, 31.7, 26.5, 26.1, 26.0, 18.4, 18.1, 15.9, -4.3, -4.4, -4.6, -4.8; HRMS (ESI): m/z $[M]^+$ calcd for 504.3455, found 504.3452.

Preparation of **17**: DMP oxidation of propargyl alcohol **16**

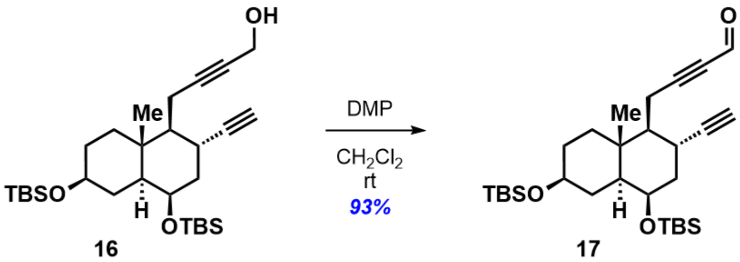

To a stirred solution of propargyl alcohol **16** (0.89 g, 1.76 mmol, 1 eq) in CH_2Cl_2 (0.05 M, 35 mL) were sequentially added $NaHCO_3$ (0.443 g, 5.28 mmol, 3 eq) and DMP (0.822 g, 1.94 mmol, 1.1 eq) at room temperature. After stirring for 10 min, the reaction mixture was dry-loaded on column packed with silica gel and purified (EtOAc/hexanes = 1:19) providing propiolaldehyde **17** (0.823 g, 93%) as a colorless oil.

R_f 0.5 (EtOAc/hexanes = 1:19), visualized with UV and $KMnO_4$ stain; $[\alpha]_D^{22}$ = –69° (*c* = 0.4, $CHCl_3$); ^{1}H NMR (500 MHz, $CDCl_3$) δ 9.17 (s, 1H), 3.72 (q, *J* = 2.7 Hz, 1H), 3.57 (tt, *J* = 11.0, 4.8 Hz, 1H), 2.85 (tt, *J* = 12.0, 3.5 Hz, 1H), 2.77 (dd, *J* = 15.3, 3.0 Hz, 1H), 2.68 (dd, *J* = 18.4, 6.5 Hz, 1H), 2.11 (d, *J* = 2.4 Hz, 1H), 2.03 (dt, *J* = 13.8, 3.4 Hz, 1H), 1.75 – 1.63 (m, 4H), 1.53 – 1.38 (m, 2H), 1.17 – 1.04 (m, 2H), 1.03 (s, 3H), 0.89 (s, 9H), 0.88 (s, 9H), 0.05 (s, 6H), 0.04 (s, 3H), 0.02 (s, 3H); ^{13}C NMR (125 MHz, $CDCl_3$) δ 177.3, 100.1, 87.1, 82.8, 72.2, 71.3, 70.4, 52.2, 47.4, 41.8, 39.3, 37.3, 36.5, 31.6, 26.7, 26.1, 26.0, 18.7, 18.4, 18.1, 15.8, -4.3, -4.4, -4.6, -4.8; HRMS (ESI): m/z $[M]^+$ calcd for 502.3298, found 502.3291.

Preparation of **19**: *N*-benzoylation of piperidone **18**

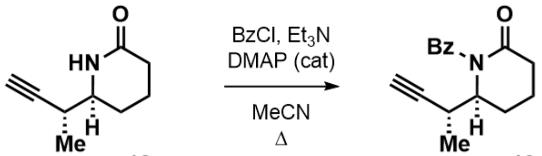

18 **19**

To a stirred solution of lactam **18**[4] (0.364 g, 2.41 mmol, 1 eq) in MeCN (0.2 M, 12 mL) were sequentially added Et_3N (1 mL, 7.23 mmol, 3 eq), DMAP (29 mg, 0.24 mmol, 0.1 eq), and BzCl (0.7 mL, 6 mmol, 2.5 eq) at room temperature. After stirring at 90 °C for 24 h, the reaction mixture was cooled to ambient temperature, treated with sat. aq. $NaHCO_3$ and vigorously stirred for another 30 minutes. The whole mixture was extracted with EtOAc/hexanes = 1:1 three times. The combined organic extract was washed with 1 M aq. HCl, brine, dried over Na_2SO_4, filtered, and evaporated providing crude material, which was purified using silica gel column chromatography (EtOAc/hexanes = 15% → 20% → 30% → 40%) furnishing imide **19** (0.58 g, 95%) as a colorless, waxy solid.

R_f 0.4 (EtOAc/hexanes = 1:2), visualized with UV and $KMnO_4$ stain; ^{1}H NMR (600 MHz, $CDCl_3$) δ 7.66 – 7.62 (m, 2H), 7.50 – 7.45 (m, 1H), 7.41 – 7.36 (m, 2H), 4.69 (q, *J* = 5.5 Hz, 1H), 3.07 (qdd, *J* = 7.0, 5.9, 2.5 Hz, 1H), 2.67 – 2.60 (m, 1H), 2.59 – 2.52 (m, 1H), 2.17 – 2.09 (m, 2H), 2.08 (d, *J* = 2.5 Hz, 1H), 2.07 – 2.00 (m, 1H), 1.91 – 1.84 (m, 1H), 1.25 (d, *J* = 7.1 Hz, 3H); ^{13}C NMR (150 MHz, $CDCl_3$) δ 174.8, 174.2, 136.3, 131.8, 128.5, 128.2, 85.5, 71.8, 57.5, 34.0, 30.4, 25.2, 18.1, 17.7; HRMS (ESI): m/z $[M]^+$ calcd for 255.1259, found 255.1249.

[4] Cassaidy, K. J.; Rawal, V. H. Enantioselective total synthesis of (+)-heilonine. *J. Am. Chem. Soc.* **2021**, *143*, 16394–16400.

Preparation of **21**: Hydroxymethylation of **19**

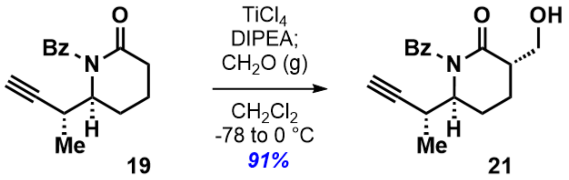

To a stirred solution of imide **19** (0.41 g, 1.6 mmol, 1 eq) in CH_2Cl_2 (0.2 M, 8 mL) was added $TiCl_4$ (1.0 M in CH_2Cl_2, 1.76 mmol, 1.76 mL) at 0 °C. After stirring for 10 min, freshly distilled DIPEA (0.33 mL, 1.92 mmol, 1.2 eq) was added, and the dark-red reaction mixture was allowed to stir for 1 h at 0 °C. Then, the mixture was cooled to -78 °C and paraformaldehyde (dried in a desiccator over P_2O_5 for 24 h prior to use; 0.24 g, 8 mmol, 5 eq) was cracked in a separate two-neck round bottom flask, and the vapors were carried by a weak steam of N_2 directly into the reaction mixture at -78 °C. After stirring for 10 min at 0 °C, the reaction mixture was quenched with sat. aq. $NaHCO_3$. The whole mixture was extracted with CH_2Cl_2 three times. The combined organic extract was dried over Na_2SO_4, filtered, and evaporated providing crude material, which was purified using silica gel column chromatography (EtOAc/hexanes = 40% → 50% → 100%) furnishing carbinol **21** (0.415 g, 91%) as a colorless oil.

R_f 0.32 (EtOAc/hexanes = 1:1), visualized with UV and $KMnO_4$ stain; ^{1}H NMR (600 MHz, $CDCl_3$) δ 7.74 – 7.70 (m, 2H), 7.54 – 7.49 (m, 1H), 7.44 – 7.40 (m, 2H), 4.75 (q, *J* = 6.1 Hz, 1H), 3.73 – 3.69 (m, 2H), 3.04 (pd, *J* = 7.0, 2.5 Hz, 1H), 2.90 – 2.78 (m, 2H), 2.21 – 2.01 (m, 3H), 2.06 (d, *J* = 2.5 Hz, 1H), 1.68 (dddd, *J* = 15.2, 12.7, 6.5, 3.3 Hz, 1H), 1.24 (d, *J* = 7.0 Hz, 3H); ^{13}C NMR (150 MHz, $CDCl_3$) δ 177.3, 174.7, 135.8, 132.4, 128.7, 128.4, 85.1, 71.8, 63.3, 58.2, 44.6, 29.8, 24.2, 21.5, 17.4; HRMS (ESI): m/z $[M]^+$ calcd for 285.1365, found 285.1369.

Preparation of **22**: DBU-mediated epimerization of carbinol **21**

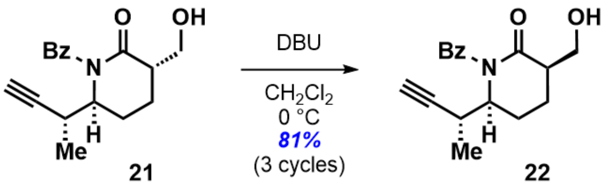

To a stirred solution of carbinol **21** (0.524 g, 1.84 mmol, 1 eq) in CH_2Cl_2 (0.1 M, 18 mL) was added DBU (0.82 mL, 5.51 mmol, 3 eq) at 0 °C. After stirring the pale-yellow reaction mixture for 6 h at 0 °C, it was quenched with sat. aq. NH_4Cl, warmed to room temperature, and stirred vigorously until decolorized (*ca.* 10 min). The layers were separated, and the aq. phase was extracted with CH_2Cl_2 three times. The combined organic extract was dried over Na_2SO_4, filtered, and evaporated providing crude material, which was purified using silica gel column chromatography (EtOAc/hexanes = 30% → 40% → 50% → 60%) giving back *trans*-carbinol **21** (0.246 g, 47%) and providing the desired *cis*-carbinol **22** (0.25 g, 48%) both as colorless oils. Two more cycles of the epimerization protocol on recovered **21** gave a total of 0.42 g of **22** (81% overall yield).

R_f 0.18 (EtOAc/hexanes = 1:1), visualized with UV and $KMnO_4$ stain; ^{1}H NMR (600 MHz, $CDCl_3$) δ 7.64 – 7.59 (m, 2H), 7.54 – 7.45 (m, 1H), 7.43 – 7.37 (m, 2H), 4.63 – 4.57 (m, 1H), 3.85 – 3.75 (m, 2H), 3.04 (qdd, *J* = 7.2, 5.7, 2.5 Hz, 1H), 2.88 (dd, *J* = 8.3, 4.0 Hz, 1H), 2.72 (dtd, *J* = 11.1, 6.9, 4.2 Hz, 1H), 2.28 – 2.19 (m, 1H), 2.09 (d, *J* = 2.5 Hz, 1H), 2.12 – 2.02 (m, 2H), 1.98 – 1.87 (m, 1H), 1.32 (d, *J* = 7.1 Hz, 3H); ^{13}C NMR (150 MHz, $CDCl_3$) δ 177.3, 174.1, 136.0, 131.6, 128.2, 128.1, 85.6, 72.6, 63.8, 57.6, 46.3, 31.2, 25.8, 21.2, 19.0; HRMS (ESI): m/z $[M]^+$ calcd for 285.1365, found 285.1369.

Preparation of **23**: THP protection of carbinol **22**

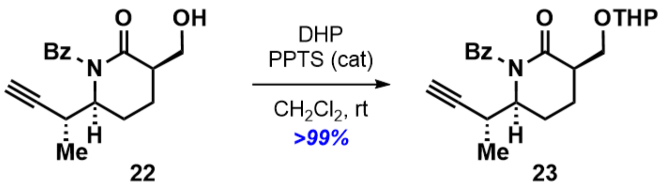

To a stirred solution of **22** (0.342 g, 1.2 mmol, 1 eq) in CH_2Cl_2 (0.1 M, 12 mL) were sequentially added DHP (0.16 mL, 1.8 mmol, 1.5 eq) and PPTS (60 mg, 0.24 mmol, 0.2 eq) at room temperature. After stirring for 48 h at room temperature, the reaction mixture was evaporated providing crude material, which was purified using silica gel column chromatography (EtOAc/hexanes = 15% → 40%) to yield THP ether **23** (0.443 g, >99%) as a colorless oil.

R_f 0.42 (EtOAc/hexanes = 1:2), visualized with UV and $KMnO_4$ stain; ^{1}H NMR (500 MHz, $CDCl_3$) δ 7.80 – 7.69 (m, 4H), 7.50 – 7.44 (m, 2H), 7.36 (t, *J* = 7.7 Hz, 4H), 4.76 – 4.69 (m, 3H), 4.67 (dd, *J* = 4.6, 2.7 Hz, 1H), 4.24 (dd, *J* = 9.4, 4.9 Hz, 1H), 4.00 – 3.84 (m, 4H), 3.61 – 3.54 (m, 2H), 3.53 (dd, *J* = 9.4, 3.2 Hz, 1H), 3.28 – 3.14 (m, 2H), 2.80 – 2.67 (m, 2H), 2.36 – 2.14 (m, 4H), 2.03 – 1.95 (m, 2H), 1.94 (d, *J* = 2.5 Hz, 1H), 1.91 (d, *J* = 2.4 Hz, 1H), 1.94 – 1.55 (m, 12H), 1.31 (d, *J* = 5.4 Hz, 3H), 1.30 (d, *J* = 5.4 Hz, 3H); ^{13}C NMR (125 MHz, $CDCl_3$) δ 175.2, 175.1, 174.6, 174.2, 136.3, 131.8, 131.7, 128.9, 128.9, 127.9, 99.5, 98.9, 86.1, 72.0, 72.0, 68.5, 68.3, 62.7, 61.6, 57.5, 57.5, 44.7, 44.6, 30.7, 30.5, 30.1, 29.9, 25.6, 25.5, 24.3, 24.1, 21.0, 20.9, 19.8, 19.1, 18.1, 17.9; HRMS (ESI): m/z $[M]^+$ calcd for 369.1940, found 369.1941.

Preparation of **24**: Hydrazine-mediated *N*-deprotection of THP ether **23**

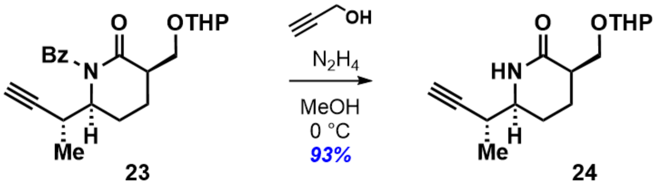

To a stirred solution of **23** (0.446 g, 1.21 mmol, 1 eq) in MeOH/propargyl alcohol = 9:1 (0.1 M, 12 mL) was added hydrazine hydrate (35% w/w, 0.32 mL, 3.62 mmol, 3 eq) at 0 °C. After stirring for 3.5 h at 0 °C, the mixture was quenched with sat. aq. NH_4Cl while cold. The mixture was then extracted with EtOAc/hexanes = 1:1 three times. Most of the $BzNHNH_2$ and propargyl alcohol remained in the aqueous layer. The remaining impurities were removed by washing the combined organic extracts successively with 0.5 M aq. HCl and brine. The washed organic layer was dried over Na_2SO_4, filtered, and concentrated to afford crude material, which was purified by silica gel column chromatography (acetone/hexanes = 20% → 30% → 40% → 50%) to yield lactam **24** (0.296 g, 93%) as a colorless oil that solidified upon refrigeration.

R_f 0.24 (acetone/hexanes = 1:2), visualized with $KMnO_4$ stain; ^{1}H NMR (500 MHz, $CDCl_3$) δ 6.21 (bs, 2H), 4.62 (t, *J* = 3.4 Hz, 1H), 4.58 (dd, *J* = 4.4, 2.8 Hz, 1H), 4.01 – 3.92 (m, 2H), 3.87 – 3.79 (m, 2H), 3.76 (dd, *J* = 9.6, 6.4 Hz, 1H), 3.65 (dd, *J* = 9.5, 3.9 Hz, 1H), 3.55 – 3.47 (m, 2H), 3.29 – 3.20 (m, 2H), 2.69 – 2.63 (m, 1H), 2.63 – 2.58 (m, 1H), 2.51 – 2.41 (m, 2H), 2.17 (d, *J* = 2.3 Hz, 1H), 2.16 (d, *J* = 2.8 Hz, 1H), 2.07 – 1.97 (m, 2H), 1.93 – 1.63 (m, 9H), 1.63 – 1.45 (m, 8H), 1.21 (d, *J* = 7.0 Hz, 3H), 1.20 (d, *J* = 7.0 Hz, 3H); ^{13}C NMR (125 MHz, $CDCl_3$) δ 172.6, 172.3, 99.9, 98.5, 85.0, 84.9, 71.7, 71.6, 69.3, 67.9, 62.5, 62.2, 56.9, 41.2, 41.1, 32.6, 32.5, 30.7, 30.7, 25.5, 23.7, 23.3, 22.6, 22.2, 19.8, 19.5, 16.9, 16.9; HRMS (ESI): m/z $[M]^+$ calcd for 265.1678, found 265.1677.

Preparation of **25**: $LiAlH_4$ reduction of secondary lactam **24**

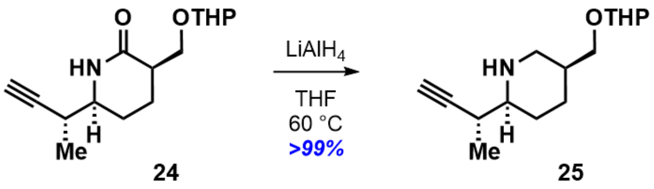

To a stirred solution of lactam **24** (0.296 g, 1.12 mmol, 1 eq) in THF (0.1 M, 11.2 mL) was dropwise added $LiAlH_4$ (1.0 M in THF, 5.6 mL, 5.6 mmol, 5 eq) at 0 °C. The reaction mixture was first allowed to stir at ambient temperature for 30 min, then the flask was placed in oil bath pre-heated to 70 °C and stirred for 20 h. Then, the mixture was cooled to room temperature and diluted with ether. The mixture was further cooled to 0 °C and slowly treated with H_2O (0.21 mL), then 15% aq. NaOH (0.21 mL), and again with H_2O (0.63 mL). The mixture was then warmed to room temperature and stirred for 15 min. Then, $MgSO_4$ was added, and the stirring was continued for 15 more minutes. The whole mixture was filtered through a pad of Celite® and washed with excess EtOAc. The filtrate was then evaporated providing piperidine **25** (0.28 g, >99%) as a colorless oil.

R_f 0.36 ($MeOH/CH_2Cl_2$ = 10%), visualized with $KMnO_4$ stain; ^1H NMR (600 MHz, $CDCl_3$) δ 4.58 (td, *J* = 4.9, 2.9 Hz, 2H), 3.92 – 3.82 (m, 3H), 3.79 (dd, *J* = 9.5, 6.6 Hz, 1H), 3.55 (dd, *J* = 9.5, 7.6 Hz, 1H), 3.52 – 3.47 (m, 2H), 3.43 (dd, *J* = 9.7, 6.8 Hz, 1H), 3.06 (ddt, *J* = 12.3, 3.9, 1.9 Hz, 2H), 2.85 (td, *J* = 12.0, 3.7 Hz, 2H), 2.69 – 2.32 (m, 4H), 2.10 (dd, *J* = 2.2, 1.2 Hz, 2H), 1.91 – 1.77 (m, 3H), 1.76 – 1.65 (m, 4H), 1.64 – 1.33 (m, 11H), 1.20 (d, *J* = 4.4 Hz, 2H), 1.19 (d, *J* = 4.5 Hz, 3H); ^{13}C NMR (150 MHz, $CDCl_3$) δ 99.3, 99.1, 86.7, 86.6, 70.4, 68.7, 68.6, 62.6, 62.5, 60.3, 59.9, 47.3, 47.3, 33.7, 33.6, 31.2, 30.9, 30.9, 25.7, 25.7, 25.6, 25.6, 25.3, 19.9, 19.8, 17.6, 17.5; HRMS (ESI): m/z $[M]^+$ calcd for 251.1885, found 251.1888.

Preparation of **26**: Reductive amination of propiolaldehyde **17** with piperidine **25**

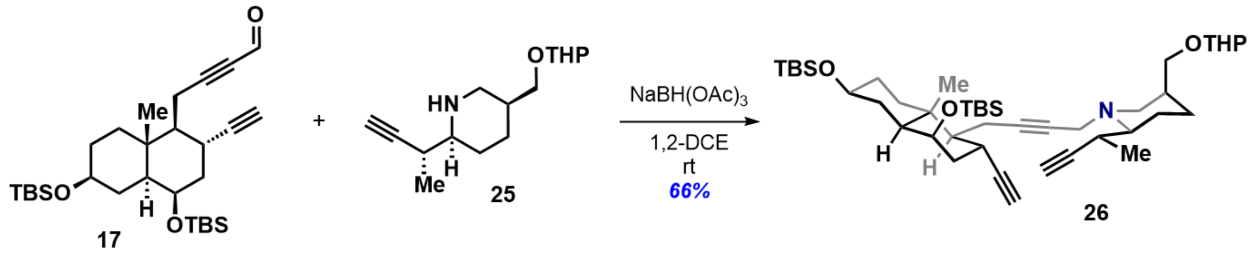

To a stirred solution of propiolaldehyde **17** (37.2 mg, 0.0074 mmol, 1 eq) and piperidine **25** (18.6 mg, 0.0074 mmol, 1 eq) in 1,2-DCE (0.025 M, 3 mL) was added $NaBH(OAc)_3$ (47 mg, 0.22 mol, 3 eq) at room temperature. After stirring for 10 min, the reaction mixture was quenched with sat. aq. $NaHCO_3$ and extracted with CH_2Cl_2 three times. The combined organic extract was dried over Na_2SO_4, filtered, and evaporated providing crude material, which was purified using silica gel column chromatography (EtOAc/hexanes = 5% → 10% → 20%) providing unreacted **17** (7.4 mg, 20%) and triyne **26** (36.1 mg, 66%) both as colorless oils.

R_f 0.37 (EtOAc/hexanes = 1:9), visualized with $KMnO_4$ stain; ^1H NMR (600 MHz, $CDCl_3$) δ 4.59 (ddd, *J* = 7.0, 4.0, 2.9 Hz, 1H), 3.94 – 3.76 (m, 2H), 3.71 (q, *J* = 2.7 Hz, 1H), 3.61 – 3.39 (m, 4H), 3.12 (dt, *J* = 17.5, 2.6 Hz, 1H), 2.96 (ddd, *J* = 6.9, 4.2, 2.5 Hz, 1H), 2.89 (d, *J* = 12.2 Hz, 1H), 2.69 (dt, *J* = 11.7, 2.9 Hz, 2H), 2.58 – 2.48 (m, 3H), 2.09 (d, *J* = 2.3 Hz, 1H), 2.06 – 1.99 (m, 1H), 2.01 (d, *J* = 2.5 Hz, 1H), 1.95 (bs, 1H), 1.86 – 1.64 (m, 8H), 1.61 – 1.36 (m, 9H), 1.31 – 1.23 (m, 1H), 1.10 (d, *J* = 7.2 Hz, 3H), 1.07 (s, 3H), 1.14 – 1.02 (m, 1H), 0.89 (s, 9H), 0.88 (s, 9H), 0.05 (s, 6H), 0.03 (s, 3H), 0.02 (s, 3H); ^{13}C NMR (150 MHz, $CDCl_3$) δ 99.2, 98.8, 88.1, 87.9, 86.0, 86.0, 75.2, 72.4, 71.5, 69.4, 69.3, 68.6, 68.2, 62.4, 62.1, 61.1, 61.0, 54.9, 54.9, 52.4, 47.6, 43.6, 43.5, 41.9, 39.5, 37.3, 36.5, 34.1, 34.0, 31.7, 30.9, 30.9, 26.7, 26.4, 26.1, 26.0, 25.7, 25.6, 25.4, 20.8, 19.8, 19.7, 18.4, 18.1, 17.9, 16.2, 13.7, 13.7, -4.3, -4.4, -4.5, -4.8; HRMS (ESI): m/z $[M]^+$ calcd for 737.5235, found 737.5239.

Preparation of **27**: Rh-catalyzed cyclotrimerization of amine-triyne **26**

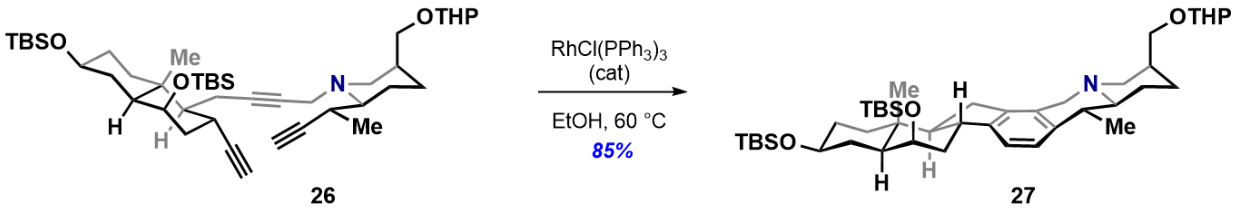

To a stirred solution of triyne **26** (42.2 mg, 0.057 mmol, 1 eq) in freshly distilled EtOH (0.025M, 2.3 mL) was added $RhCl(PPh_3)_3$ (5.3 mg, 0.006 mmol, 10 mol%) at room temperature. The initially heterogenous mixture was placed in oil bath pre-heated to 60 °C and stirred for 1 h. Then, the reaction mixture was cooled to ambient temperature and evaporated to dryness providing crude material, which was purified using silica gel column chromatography (acetone/hexanes = 10% → 15% → 20%) to afford hexacycle **27** (35.9 mg, 85%) as a colorless oil that solidified upon refrigeration.

R_f 0.29 (acetone/hexanes = 1:4), visualized with $KMnO_4$ stain; ^{1}H NMR (600 MHz, $CDCl_3$) δ 7.07 (d, *J* = 7.7 Hz, 1H), 6.95 (d, *J* = 7.7 Hz, 1H), 4.55 (td, *J* = 4.6, 2.9 Hz, 1H), 3.91 (q, *J* = 2.6 Hz, 1H), 3.89 – 3.81 (m, 2H), 3.67 – 3.59 (m, 2H), 3.58 – 3.50 (m, 1H), 3.50 – 3.42 (m, 1H), 3.42 – 3.33 (m, 1H), 3.22 (t, *J* = 12.3 Hz, 1H), 2.94 – 2.82 (m, 1H), 2.76 (q, *J* = 7.9 Hz, 1H), 2.54 (ddd, *J* = 14.5, 7.0, 4.0 Hz, 1H), 2.46 (td, *J* = 13.1, 7.0 Hz, 1H), 2.38 – 2.25 (m, 2H), 2.06 – 2.00 (m, 2H), 1.93 – 1.86 (m, 1H), 1.86 – 1.78 (m, 1H), 1.78 – 1.65 (m, 4H), 1.63 – 1.44 (m, 9H), 1.26 (d, *J* = 6.9 Hz, 3H), 1.12 (s, 3H), 1.12 – 1.06 (m, 1H), 0.90 (s, 9H), 0.89 (s, 9H), 0.07 (s, 9H), 0.06 (s, 3H); ^{13}C NMR (150 MHz, $CDCl_3$) δ 144.2, 139.9, 139.9, 137.0, 130.4, 130.3, 125.2, 125.1, 120.1, 120.0, 99.3, 99.3, 73.4, 73.0, 69.2, 69.1, 65.2, 64.8, 62.7, 62.6, 61.4, 61.4, 56.5, 56.4, 49.2, 39.4, 38.8, 38.0, 36.3, 36.0, 34.9, 34.6, 31.8, 30.9, 30.9, 29.9, 28.9, 28.9, 26.1, 26.0, 25.7, 25.6, 20.0, 19.9, 19.7, 19.6, 18.4, 18.1, 15.8, -4.3, -4.3, -4.4, -4.7; HRMS (ESI): m/z $[M]^+$ calcd for 737.5235, found 737.5239.

Preparation of **28**: Deprotection/sulfonylation of hexacyclic acetal **27**

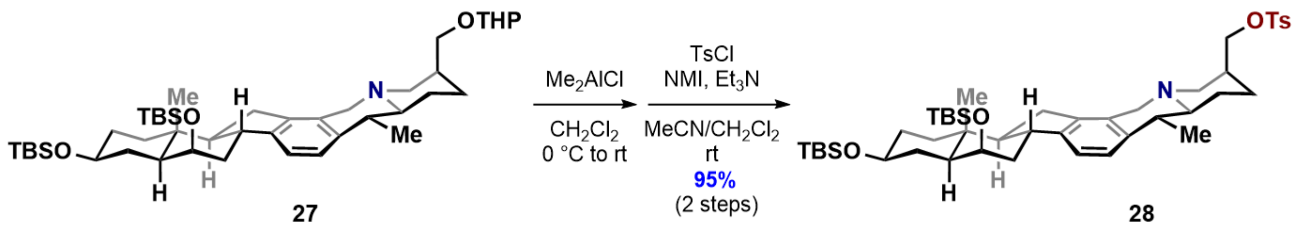

To a stirred solution of **27** (21 mg, 0.028 mmol, 1 eq) in CH_2Cl_2 (0.025 M, 1.1 mL) was dropwise added Me_2AlCl (0.9 M in heptane; 0.13 mL, 0.114 mmol, 4 eq) at 0 °C The reaction mixture was slowly allowed to reach room temperature over 1 h. After stirring at room temperature for 6 h, the mixture was quenched with sat. aq. $NaHCO_3$ and stirred for additional 5 h at ambient temperature. Then, the mixture was diluted with brine and extracted with CH_2Cl_2 three times. The combined organic extract was dried over Na_2SO_4, filtered, and evaporated providing crude material, which was used in the next step without further purification.

To a stirred solution of crude aminoalcohol intermediate in $MeCN/CH_2Cl_2$ = 4:1 (0.025 M, 1.3 mL) were sequentially added Et_3N (24 μL, 0.175 mmol, 6.2 eq), NMI (8 μL, 0.1 mmol, 3.6 eq), and TsCl (12 mg, 0.064 mmol, 2.3 eq) at room temperature. After stirring for 1 h, the mixture was evaporated to dryness providing crude material, which was purified using silica gel column chromatography (EtOAc/hexanes = 1:4 → 1:2 → 1:1) to afford tosylate **28** (21.8 mg, 95%) as a white solid.

R_f 0.52 (EtOAc/hexanes = 1:2), visualized with UV and $KMnO_4$ stain; ^1H NMR (600 MHz, $CDCl_3$) δ 7.79 – 7.74 (m, 2H), 7.27 (d, *J* = 8.5 Hz, 2H), 7.04 (d, *J* = 7.8 Hz, 1H), 6.96 (d, *J* = 7.7 Hz, 1H), 4.23 (dd, *J* = 9.7, 8.1 Hz, 1H), 4.14 (dd, *J* = 9.7, 6.8 Hz, 1H), 3.92 (q, *J* = 2.6 Hz, 1H), 3.63 (tt, *J* = 10.4, 4.7 Hz, 1H), 3.48 (d, *J* = 15.1 Hz, 1H), 3.26 – 3.19 (m, 2H), 2.80 (d, *J* = 11.8 Hz, 1H), 2.60 (t, *J* = 7.7 Hz, 1H), 2.52 (dd, *J* = 14.5, 6.9 Hz, 1H), 2.44 (dd, *J* = 13.1, 13.1 Hz, 1H), 2.37 (s, 3H), 2.28 (tt, *J* = 8.5, 3.2 Hz, 2H), 2.17 – 2.10 (m, 1H), 1.94 – 1.82 (m, 2H), 1.81 – 1.68 (m, 3H), 1.63 – 1.44 (m, 6H), 1.34 – 1.27 (m, 2H), 1.22 (d, *J* = 6.9 Hz, 3H), 1.13 (s, 3H), 1.12 – 1.06 (m, 2H), 0.91 (s, 9H), 0.89 (s, 9H), 0.08 (s, 9H), 0.06 (s, 3H); ^{13}C NMR (150 MHz, $CDCl_3$) δ 144.6, 144.1, 139.6, 136.5, 133.1, 130.0, 129.7, 127.9, 124.9, 120.1, 73.2, 72.9, 71.7, 65.2, 61.2, 56.2, 49.0, 39.3, 38.7, 37.9, 37.6, 36.1, 35.9, 34.1, 33.6, 31.7, 28.8, 26.7, 26.0, 25.8, 24.2, 22.4, 21.5, 19.4, 18.3, 18.0, 15.7, 14.1, -4.4, -4.4, -4.6, -4.8; HRMS (ESI): m/z $[M]^+$ calcd for 807.4748, found 807.4756.

Preparation of **30**: Intramolecular *N*-alkylation/Stevens sequence on tosylate **28**

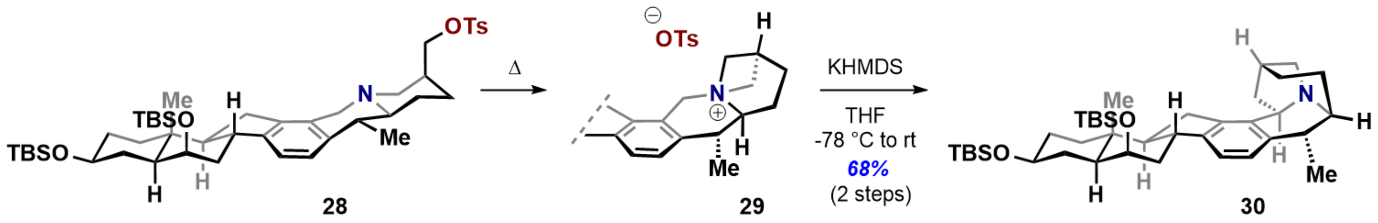

A flame-dried 250 mL round-bottom flask was charged with tosylate **28** (0.141 g, 0.174 mmol, 1 eq), a Teflon®-coated stir bar, and MeCN (freshly distilled from CaH_2; 0.0017 M, 100 mL). The vessel was sealed under the atmosphere of Ar and sonicated until substrate dissolved. A clear, colorless solution was then placed in oil bath pre-heated to 90 °C and stirred for 13 h. The mixture was then cooled to ambient temperature and evaporated to dryness providing *ca.* 7:3 mixture of azetidinium **29** and starting tosylate **28**. The mixture was transferred to a 50 mL round-bottom flask, azeotroped with benzene, and used in the subsequent step as such.

To a stirred solution of the mixture obtained from previous step in THF (0.025M, 7 mL) was dropwise added KHMDS (1.0 M in THF; 0.35 mL, 0.35 mmol, 2 eq) at -78 °C. The reaction mixture was first stirred at -78 °C for 10 min, then warmed to 0 °C and stirred for additional 20 min. The reaction mixture was then quenched with sat. aq. $NaHCO_3$, diluted with water, and extracted with CH_2Cl_2 four times. The combined organic extract was dried over Na_2SO_4, filtered, and evaporated providing crude material, which was purified using silica gel column chromatography (MeOH/CH_2Cl_2/aq. NH_4OH = 5:94.5:0.5 → 10:89.5:0.5 → 15:84.5:0.5) to afford unreacted tosylate **28** (44 mg, 31%) as a white solid and amine **30** (76 mg, 68%) as a white, amorphous solid.

R_f 0.62 (MeOH/CH_2Cl_2 = 10%), visualized with PMA stain; mp (MeCN) 129-131 °C; ^{1}H NMR (500 MHz, $CDCl_3$) δ 6.99 (d, *J* = 7.7 Hz, 1H), 6.96 (d, *J* = 7.7 Hz, 1H), 4.18 (dd, *J* = 10.3, 6.2 Hz, 1H), 3.95 (d, *J* = 2.7 Hz, 1H), 3.67 (td, *J* = 10.6, 5.2 Hz, 1H), 3.39 – 3.29 (m, 1H), 3.08 – 2.99 (m, 2H), 2.94 (dd, *J* = 11.3, 1.8 Hz, 1H), 2.61 (p, *J* = 6.6 Hz, 2H), 2.49 – 2.40 (m, 1H), 2.40 – 2.25 (m, 3H), 1.86 – 1.70 (m, 3H), 1.68 – 1.48 (m, 5H), 1.43 (d, *J* = 11.8 Hz, 1H), 1.38 (d, *J* = 7.1 Hz, 3H), 1.36 – 1.22 (m, 3H), 1.18 (s, 3H), 1.16 – 1.10 (m, 1H), 0.93 (s, 9H), 0.92 (s, 9H), 0.11 (s, 3H), 0.10 (s, 6H), 0.09 (s, 3H); ^{13}C NMR (125 MHz, $CDCl_3$) δ 144.5, 140.9, 135.8, 133.0, 126.9, 119.8, 73.3, 72.9, 61.6, 60.5, 59.0, 49.0, 40.1, 39.2, 39.0, 38.2, 37.8, 36.1, 35.9, 34.3, 31.7, 30.6, 29.8, 26.0, 25.9, 25.3, 22.9, 18.3, 18.0, 15.7, -4.4, -4.6, -4.9; HRMS (ESI): m/z $[M]^+$ calcd for 635.4554, found 635.4550.

Preparation of **32**: Oxidation of the Stevens product **30**

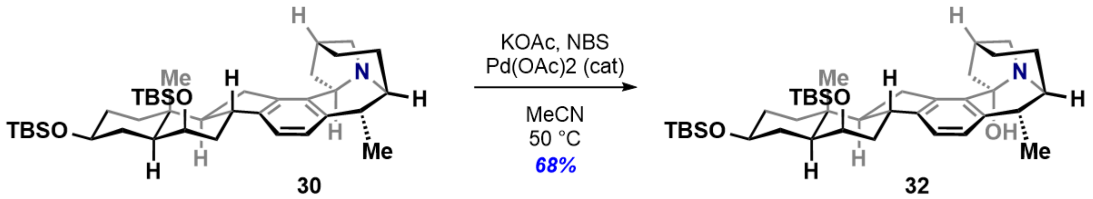

A flame-dried 25 mL round-bottom flask was charged with amine **30** (46 mg, 0.072 mmol, 1 eq), $Pd(OAc)_2$ (3.2 mg, 0.014 mmol, 20 mol%), KOAc (28 mg, 0.29 mmol, 4 eq), NBS (25 mg, 0.14 mmol, 2 eq) and a Teflon®-coated stir bar under N_2 atmosphere. MeCN (freshly distilled from CaH_2; 0.02 M, 3.6 mL) was added, the flask was sealed, and the heterogenous reaction mixture was placed in oil bath pre-heated to 50 °C. After stirring for 2 h, the reaction mixture was cooled to ambient temperature, dry-loaded on silica gel, and purified (MeOH/CH_2Cl_2/aq. NH_4OH = 5:95:1 → 10:89:1 → 15:84:1) to afford aminal **32** (32 mg, 68%) as a white, amorphous solid.

R_f 0.54 (MeOH/CH_2Cl_2 = 10%), visualized with PMA stain; ^{1}H NMR (600 MHz, pyridine-d_5) δ 7.17 (d, *J* = 7.5 Hz, 1H), 7.01 (d, *J* = 7.5 Hz, 1H), 3.99 – 3.86 (m, 2H), 3.82 (dd, *J* = 14.7, 6.6 Hz, 1H), 3.73 (dq, *J* = 10.9, 5.3 Hz, 1H), 3.38 (td, *J* = 12.1, 3.4 Hz, 1H), 3.21 – 3.08 (m, 1H), 2.78 (d, *J* = 11.1 Hz, 1H), 2.75 (dd, *J* = 13.3, 6.8 Hz, 1H), 2.67 (dd, *J* = 14.5, 12.5 Hz, 1H), 2.55 (q, *J* = 7.1 Hz, 1H), 2.43 (dt, *J* = 12.9, 3.2 Hz, 1H), 2.40 – 2.34 (m, 1H), 1.99 (dd, *J* = 13.1, 1.8 Hz, 1H), 1.92 (td, *J* = 12.6, 10.8 Hz, 1H), 1.77 (d, *J* = 12.4 Hz, 1H), 1.74 – 1.61 (m, 3H), 1.61 – 1.45 (m, 6H), 1.50 (d, *J* = 7.1 Hz, 3H), 1.45 – 1.39 (m, 2H), 1.38 – 1.32 (m, 1H), 1.23 (s, 3H), 1.10 – 1.06 (m, 1H), 0.98 (d, *J* = 0.8 Hz, 9H), 0.94 (s, 9H), 0.19 (s, 3H), 0.17 (s, 3H), 0.16 (s, 3H), 0.13 (s, 3H); ^{13}C NMR (150 MHz, pyridine-d_5) δ 146.0, 142.9, 136.6, 134.9, 127.0, 121.4, 92.7, 73.4, 72.8, 61.8, 61.6, 58.6, 48.7, 46.6, 39.8, 38.9, 38.9, 38.2, 36.4, 35.8, 35.3, 32.0, 31.0, 29.9, 25.9, 25.8, 24.7, 22.3, 18.1, 17.9, 15.5, -4.4, -4.4, -4.7, -5.0; HRMS (ESI): m/z $[M]^+$ calcd for 651.4503, found 651.4513.

Preparation of **1**: TfOH-mediated global deprotection of hemiaminal **32**

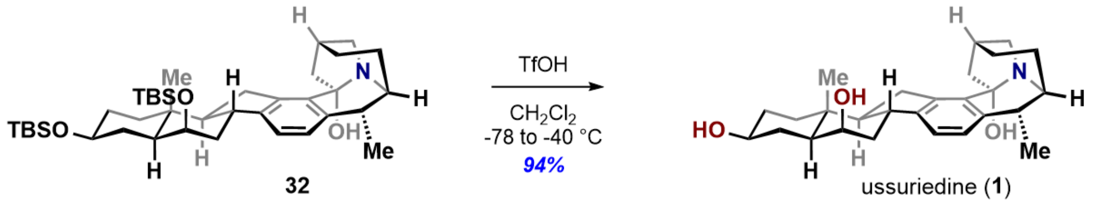

To a stirred solution of **32** (32 mg, 0.049 mmol, 1 eq) in CH_2Cl_2 (0.01 M, 4.9 mL) was added TfOH (0.17 mL, 1.96 mmol, 40 eq) at -78 °C. The reaction mixture was warmed to -40 °C, stirred for 10 min, and quenched with excess 1 M aq. NaOH. The mixture was warmed to ambient temperature and vigorously stirred for 10 more minutes. Then, the whole mixture was extracted with $CHCl_3$ three times. The combined organic extract was dried over $MgSO_4$, filtered through a pad of Celite®, and evaporated providing crude material, which was purified using silica gel column chromatography ($MeOH/CH_2Cl_2$/aq. NH_4OH = 10:89:1 → 15:84:1 → 20:79:1) providing ussuriedine (**1**, 19 mg, 94%) as a white solid.

R_f 0.17 ($MeOH/CH_2Cl_2$ = 10%), visualized with PMA stain; $[\alpha]_D^{22}$ = +20° (*c* = 1.0, $CHCl_3$); mp (ether/pentane)190-192 °C; ^{1}H NMR (600 MHz, pyridine-*d*$_5$) δ 7.04 (d, *J* = 7.6 Hz, 1H), 6.84 (d, *J* = 7.6 Hz, 1H), 6.03 (bs, 1H), 5.79 (bs, 1H), 4.89 (bs, 1H), 4.05 (dd, *J* = 2.9, 2.8 Hz, 1H), 3.82 – 3.73 (m, 2H), 3.65 (d, *J* = 10.9 Hz, 1H), 3.55 (ddd, *J* = 11.8, 11.5, 3.2 Hz, 1H), 2.92 – 2.81 (m, 1H), 2.66 – 2.49 (m, 4H), 2.36 (dd, *J* = 7.1, 7.0 Hz, 1H), 2.26 (q, *J* = 12.2 Hz, 1H), 2.21 – 2.15 (m, 1H), 1.97 – 1.89 (m, 2H), 1.82 – 1.71 (m, 2H), 1.56 – 1.47 (m, 3H), 1.44 (ddd, *J* = 13.0, 3.6, 3.6 Hz, 1H), 1.37 (s, 3H), 1.31 (d, *J* = 7.0 Hz, 3H), 1.28 – 1.16 (m, 2H), 1.16 – 1.05 (m, 3H), 0.93 (td, *J* = 13.5, 3.8 Hz, 1H); ^{13}C NMR (150 MHz, pyridine-*d*$_5$) δ 146.5, 143.4, 137.2, 136.7, 127.1, 121.5, 92.5, 72.1, 71.6, 62.5, 62.0, 59.1, 49.4, 47.0, 40.3, 39.5, 39.5, 38.9, 36.6, 36.3, 36.1, 32.5, 31.4, 30.6, 25.2, 22.6, 15.9; HRMS (ESI): m/z $[M]^+$ calcd for 423.2773, found 423.2764.

Data Matching

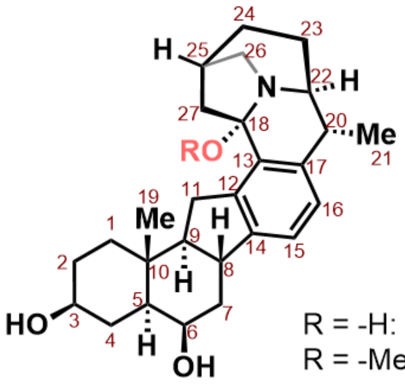

R = -H: ussuriedine (**1**)
R = -Me: ussurienine (**2**)

[C] No.	Synthetic **1** (150 MHz)	Natural **1**[a] (22.5 MHz)	Δδ, ppm ref. a	Synthetic **2** (125 MHz)	Natural **2**[a] (100 MHz)	Natural **2**[b] (N/A)	Δδ, ppm ref. a / b
**1**	39.6	39.6	0.0	39.6	39.6	39.6	0.0 / 0.0
**2**	32.5	32.4	+0.1	32.4	32.5	32.4	–0.1 / 0.0
**3**	71.6	71.6	0.0	71.6	71.6	71.6	0.0 / 0.0
**4**	36.6	36.6	0.0	36.4	36.6	36.4	–0.2 / 0.0
**5**	49.4	49.5	–0.1	49.4	49.4	49.4	0.0 / 0.0
**6**	72.1	72.2	–0.1	72.1	72.1	72.1	0.0 / 0.0
**7**	38.9	38.9	0.0	38.6	38.7	38.6	–0.1 / 0.0
**8**	39.5	39.5	0.0	39.4	39.5	40.1	+0.1 / 0.0
**9**	62.5	62.5	0.0	62.8	62.8	62.8	0.0 / 0.0
**10**	36.3	36.3	0.0	36.3	36.4	36.3	–0.1 / 0.0
**11**	31.4	31.4	0.0	31.2	31.2	31.2	0.0 / 0.0
**12**	146.5	146.4	+0.1	146.4	146.4	146.4	0.0 / 0.0
**13**	137.2	137.4	–0.2	139.5	139.5	139.5	0.0 / 0.0
**14**	143.4	143.4	0.0	142.7	142.7	142.7	0.0 / 0.0
**15**	121.5	121.3	+0.2	121.6	122.0	121.6	+0.4 / 0.0
**16**	127.1	127.0	+0.1	127.3	127.4	127.3	+0.1 / 0.0
**17**	136.7	136.9	–0.2	131.8	131.9	131.8	+0.1 / 0.0
**18**	92.5	92.4	+0.1	97.8	97.8	97.8	0.0 / 0.0
**19**	15.9	15.9	0.0	15.9	15.9	15.9	0.0 / 0.0
**20**	40.3	40.4	–0.1	40.1	40.1	39.4	0.0 / 0.0
**21**	22.6	22.6	0.0	22.5	22.5	22.5	0.0 / 0.0
**22**	62.0	62.1	–0.1	61.6	61.6	61.6	0.0 / 0.0
**23**	25.2	25.3	–0.1	25.3	25.2	25.3	+0.1 / 0.0
**24**	30.6	30.8	–0.2	30.6	30.6	30.6	0.0 / 0.0
**25**	36.1	36.3	–0.2	35.5	35.5	35.5	0.0 / 0.0
**26**	59.1	59.2	–0.1	58.7	58.7	58.7	0.0 / 0.0
**27**	47.0	47.1	–0.1	47.0	46.9	47.0	–0.1 / 0.0
**-OMe**	-	-	-	50.2	50.2	50.2	0.0 / 0.0

[a] ussuriedine/ussurienine full paper: *Tetrahedron* **1989**, 5755
[b] original communication describing isolation of ussurienine: *TL* **1988**, 1959

The isolation team did not provide complete ^{1}H NMR data; however, the ^{13}C NMR data for both **1** and **2** obtained in this work are in perfect agreement with the reported values (see table).

Ussurienine (**2**) was obtained as follows: A sample of **1** was dissolved in methanolic 1 M HCl and refluxed for 24 h. The reaction mixture was then basified with 1 M aq. NaOH and extracted with $CHCl_3$ three times. The combined organic extracts were dried over Na_2SO_4, filtered, and evaporated to provide **2**.

Below are several stacked ^{13}C NMR spectra for **1**, illustrating the necessity of a base wash prior to NMR analysis. Ussurienine (**2**), in contrast, did not exhibit any peak shifting: samples obtained directly after column chromatography gave spectra identical to those obtained recorded following the base-wash procedure.

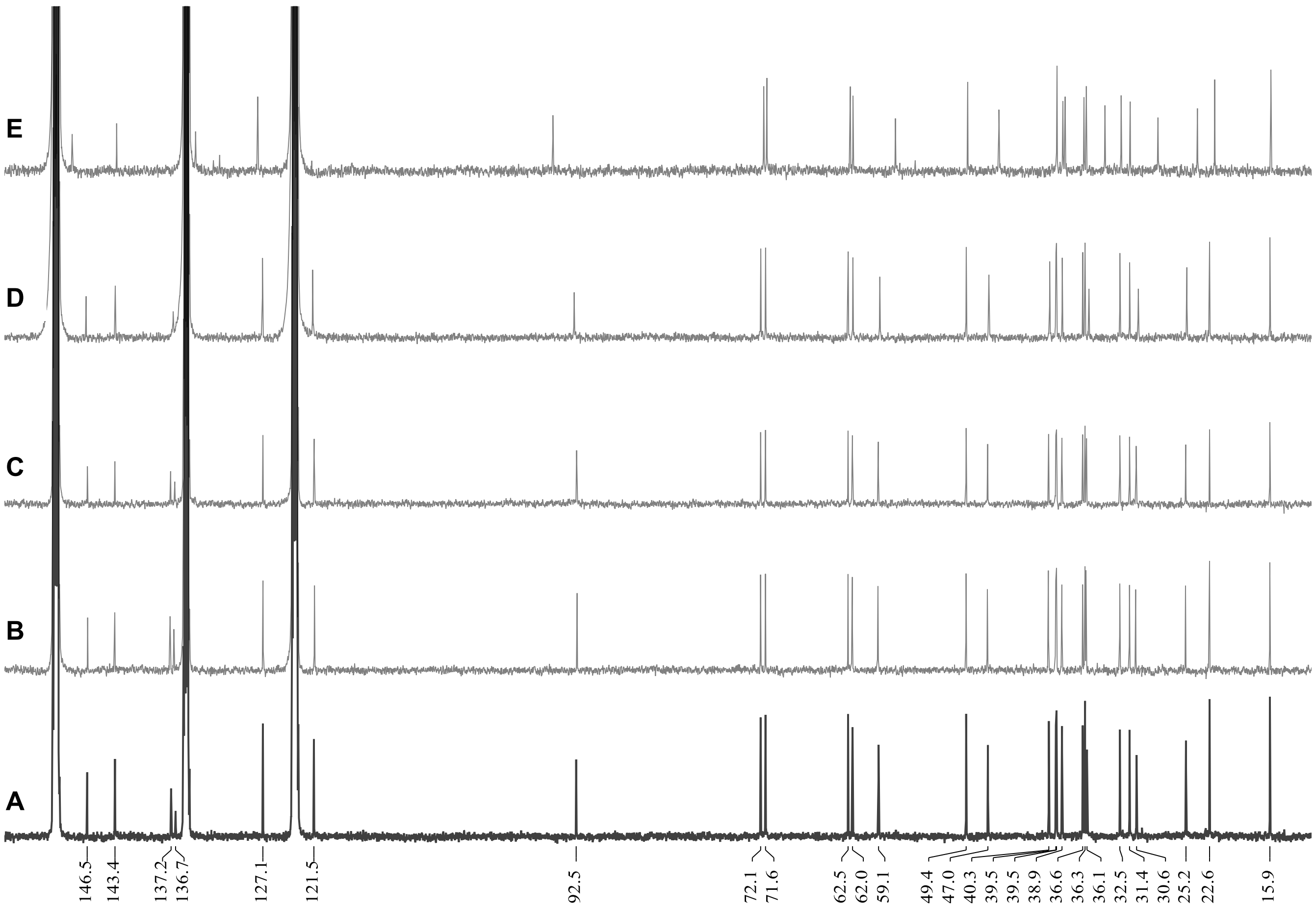

^{13}C NMR spectra of ussuriedine (**1**): **A** – base-washed sample; **B** – evaporation/redissolution (1x); **C** – evaporation/redissolution (2x); **D** – evaporation/redissolution (3x); **E** – sample straight after column chromatography.

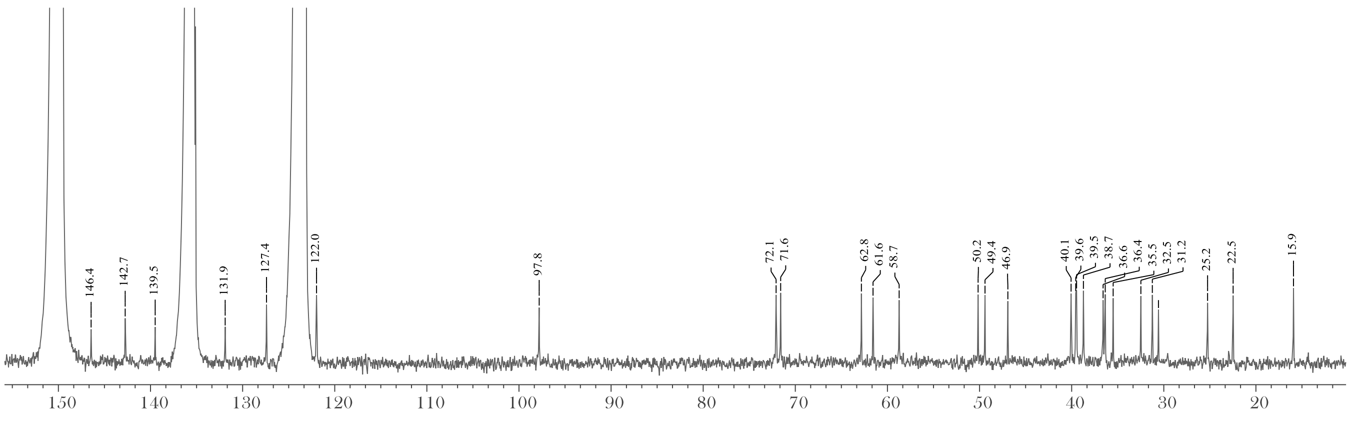

^{13}C NMR spectrum of ussurienine (**2**)

NMR Spectra

^{1}H NMR Spectrum of **S2** (400 MHz, $CDCl_3$, 25 °C) 34
^{13}C NMR Spectrum of **S2** (100 MHz, $CDCl_3$, 25 °C) 35
^{1}H NMR Spectrum of **S5** (400 MHz, $CDCl_3$, 25 °C) 36
^{13}C NMR Spectrum of **S5** (100 MHz, $CDCl_3$, 25 °C) 37
^{1}H–^{1}H COSY Spectrum of **S5** (400 MHz, $CDCl_3$, 25 °C) 38
^{1}H–^{13}C HSQC Spectrum of **S5** (100 MHz, $CDCl_3$, 25 °C) 39
^{1}H NMR Spectrum of **S6** (600 MHz, $CDCl_3$, 25 °C) 40
^{13}C NMR Spectrum of **S6** (150 MHz, $CDCl_3$, 25 °C) 41
^{1}H NMR Spectrum of **S8** (500 MHz, $CDCl_3$, 25 °C) 42
^{13}C NMR Spectrum of **S8** (125 MHz, $CDCl_3$, 25 °C) 43
^{1}H NMR Spectrum of **S9** (500 MHz, $CDCl_3$, 25 °C) 44
^{13}C NMR Spectrum of **S9** (125 MHz, $CDCl_3$, 25 °C) 45
^{1}H NMR Spectrum of **S10** (600 MHz, $CDCl_3$, 25 °C) 46
^{13}C NMR Spectrum of **S10** (150 MHz, $CDCl_3$, 25 °C) 47
^{1}H NMR Spectrum of **S11** (400 MHz, $CDCl_3$, 25 °C) 48
^{13}C NMR Spectrum of **S11** (100 MHz, $CDCl_3$, 25 °C) 49
^{1}H–^{1}H COSY Spectrum of **S11** (400 MHz, $CDCl_3$, 25 °C) 50
^{1}H–^{13}C HSQC Spectrum of **S11** (100 MHz, $CDCl_3$, 25 °C) 51
^{1}H NMR Spectrum of **S12** (400 MHz, $CDCl_3$, 25 °C) 52
^{13}C NMR Spectrum of **S12** (100 MHz, $CDCl_3$, 25 °C) 53
^{1}H–^{1}H COSY Spectrum of **S12** (400 MHz, $CDCl_3$, 25 °C) 54
^{1}H–^{13}C HSQC Spectrum of **S12** (100 MHz, $CDCl_3$, 25 °C) 55
^{1}H NMR Spectrum of **12** (400 MHz, $CDCl_3$, 25 °C) 56
^{13}C NMR Spectrum of **12** (100 MHz, $CDCl_3$, 25 °C) 57
^{1}H–^{1}H COSY Spectrum of **12** (400 MHz, $CDCl_3$, 25 °C) 58
^{1}H–^{13}C HSQC Spectrum of **12** (100 MHz, $CDCl_3$, 25 °C) 59
^{1}H NMR Spectrum of **13** (500 MHz, $CDCl_3$, 25 °C) 60
^{13}C NMR Spectrum of **13** (125 MHz, $CDCl_3$, 25 °C) 61
^{1}H NMR Spectrum of **14** (500 MHz, $CDCl_3$, 25 °C) 62
^{13}C NMR Spectrum of **14** (125 MHz, $CDCl_3$, 25 °C) 63
^{1}H NMR Spectrum of **15** (500 MHz, $CDCl_3$, 25 °C) 64
^{13}C NMR Spectrum of **15** (125 MHz, $CDCl_3$, 25 °C) 65
^{1}H NMR Spectrum of **16** (500 MHz, $CDCl_3$, 25 °C) 66
^{13}C NMR Spectrum of **16** (125 MHz, $CDCl_3$, 25 °C) 67
^{1}H NMR Spectrum of **17** (600 MHz, $CDCl_3$, 25 °C) 68
^{13}C NMR Spectrum of **17** (150 MHz, $CDCl_3$, 25 °C) 69
^{1}H NMR Spectrum of **19** (600 MHz, $CDCl_3$, 25 °C) 70

^{13}C NMR Spectrum of **19** (150 MHz, $CDCl_3$, 25 °C) 71
^{1}H NMR Spectrum of **21** (600 MHz, $CDCl_3$, 25 °C) 72
^{13}C NMR Spectrum of **21** (150 MHz, $CDCl_3$, 25 °C) 73
^{1}H NMR Spectrum of **22** (600 MHz, $CDCl_3$, 25 °C) 74
^{13}C NMR Spectrum of **22** (150 MHz, $CDCl_3$, 25 °C) 75
^{1}H–^{13}C HSQC Spectrum of **22** (150 MHz, $CDCl_3$, 25 °C) 76
^{1}H NMR Spectrum of **23** (500 MHz, $CDCl_3$, 25 °C) 77
^{13}C NMR Spectrum of **23** (125 MHz, $CDCl_3$, 25 °C) 78
^{1}H NMR Spectrum of **24** (500 MHz, $CDCl_3$, 25 °C) 79
^{13}C NMR Spectrum of **24** (125 MHz, $CDCl_3$, 25 °C) 80
^{1}H NMR Spectrum of **25** (600 MHz, $CDCl_3$, 25 °C) 81
^{13}C NMR Spectrum of **25** (150 MHz, $CDCl_3$, 25 °C) 82
^{1}H NMR Spectrum of **26** (600 MHz, $CDCl_3$, 25 °C) 83
^{13}C NMR Spectrum of **26** (150 MHz, $CDCl_3$, 25 °C) 84
^{1}H NMR Spectrum of **27** (600 MHz, $CDCl_3$, 25 °C) 85
^{13}C NMR Spectrum of **27** (150 MHz, $CDCl_3$, 25 °C) 86
^{1}H NMR Spectrum of **28** (600 MHz, $CDCl_3$, 25 °C) 87
^{13}C NMR Spectrum of **28** (150 MHz, $CDCl_3$, 25 °C) 88
^{1}H–^{13}C HSQC Spectrum of **28** (150 MHz, $CDCl_3$, 25 °C) 89
^{1}H–^{13}C HMBC Spectrum of **28** (150 MHz, $CDCl_3$, 25 °C) 90
^{1}H NMR Spectrum of **30** (500 MHz, $CDCl_3$, 25 °C) 91
^{13}C NMR Spectrum of **30** (125 MHz, $CDCl_3$, 25 °C) 92
^{1}H NMR Spectrum of **32** (600 MHz, pyridine-d_5, 25 °C) 93
^{13}C NMR Spectrum of **32** (150 MHz, pyridine-d_5, 25 °C) 94
^{1}H–^{13}C HSQC Spectrum of **32** (150 MHz, pyridine-d_5, 25 °C) 95
^{1}H–^{13}C HMBC Spectrum of **32** (150 MHz, pyridine-d_5, 25 °C) 96
^{1}H NMR Spectrum of Ussuriedine (**1**, 600 MHz, pyridine-d_5, 25 °C) 97
^{13}C NMR Spectrum of Ussuriedine (**1**, 150 MHz, pyridine-d_5, 25 °C) 98
Yang's ^{1}H NMR Spectrum of Natural Ussuriedine (**1**, methanol-d_4, 25 °C) 99
Yang's ^{13}C NMR Spectrum of Natural Ussuriedine (**1**, methanol-d_4, 25 °C) 100

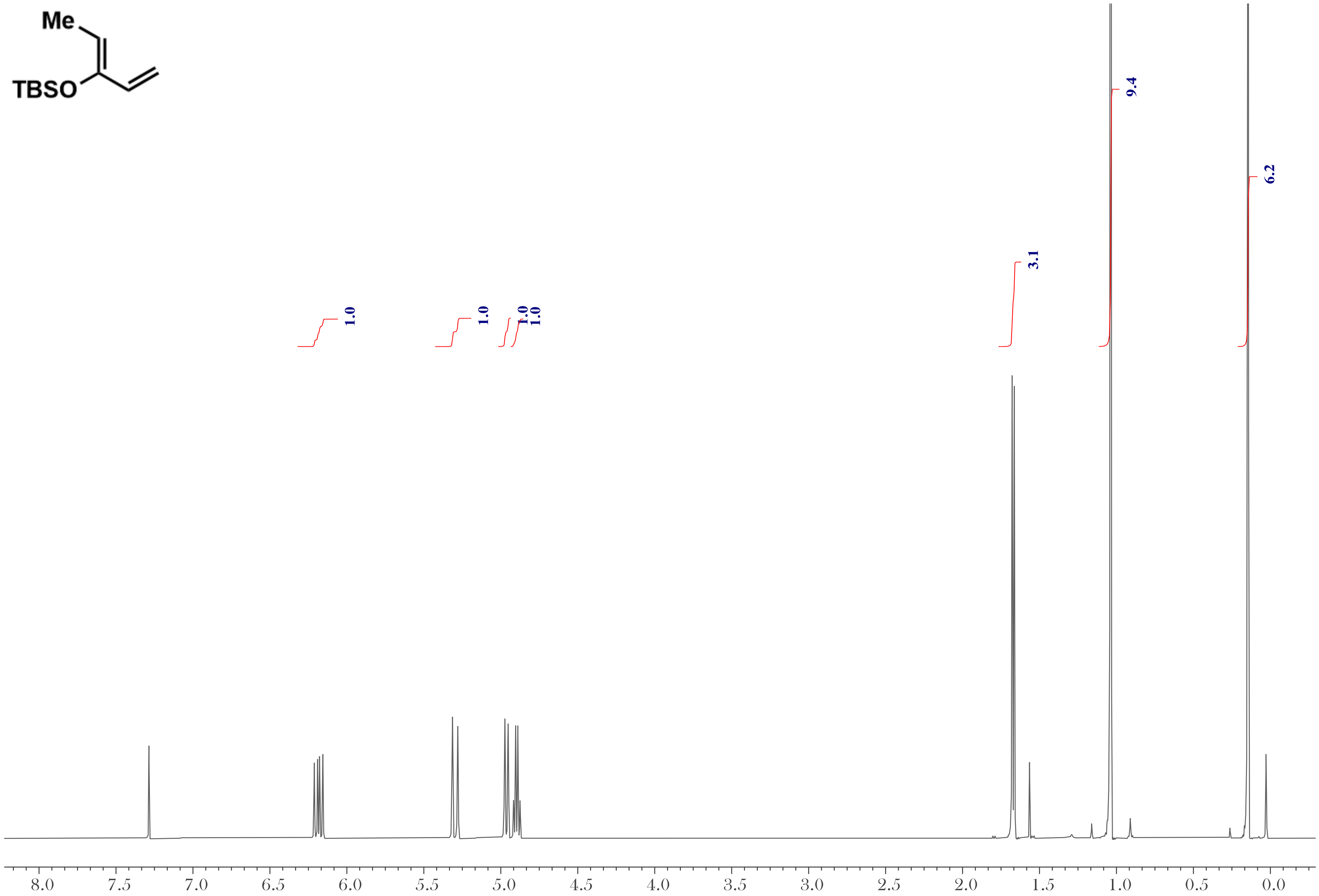

^{1}H NMR Spectrum of **S2** (400 MHz, $CDCl_3$, 25 °C)

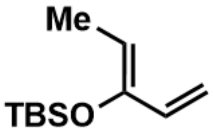

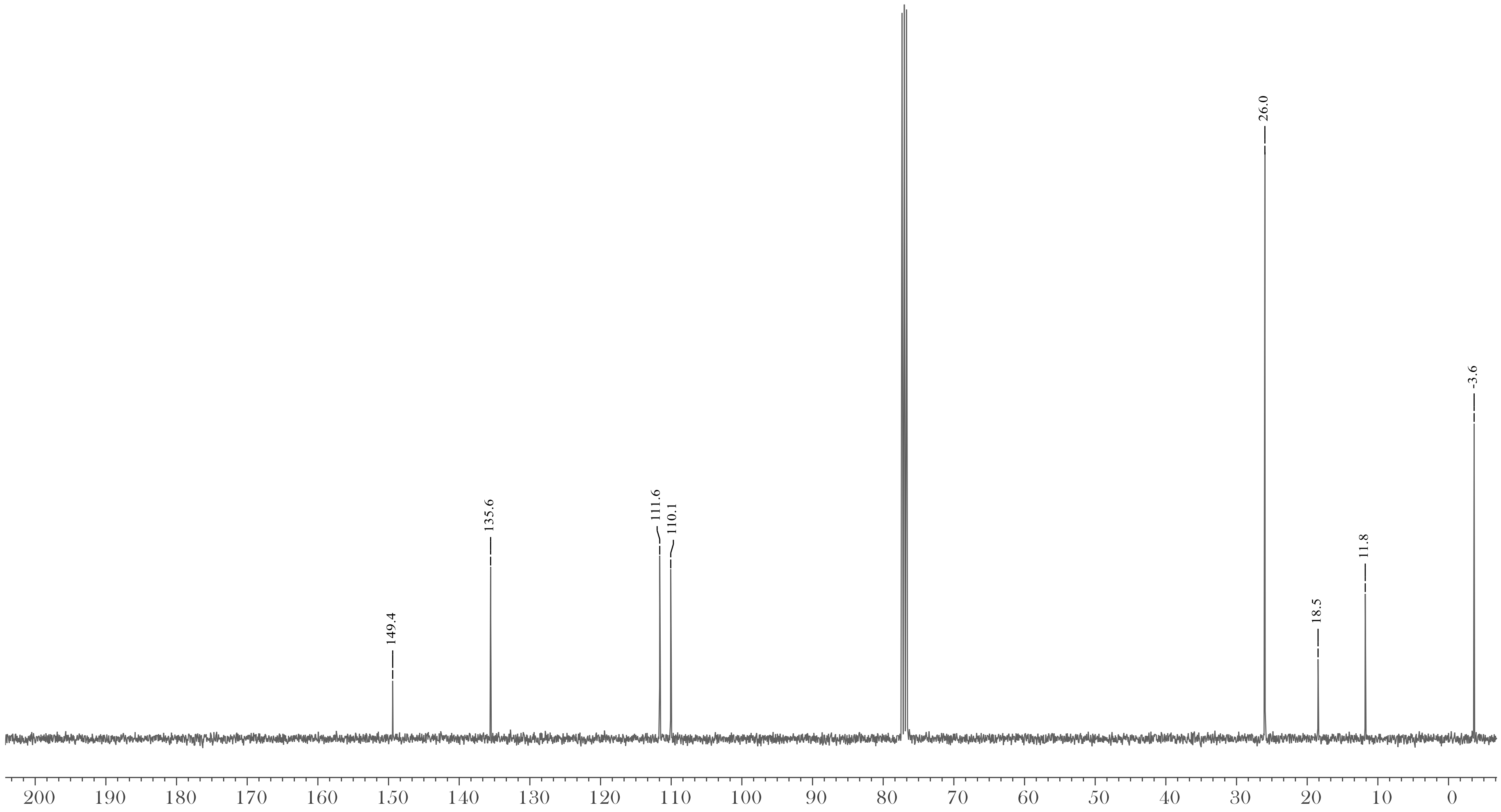

^{13}C NMR Spectrum of **S2** (100 MHz, $CDCl_3$, 25 °C)

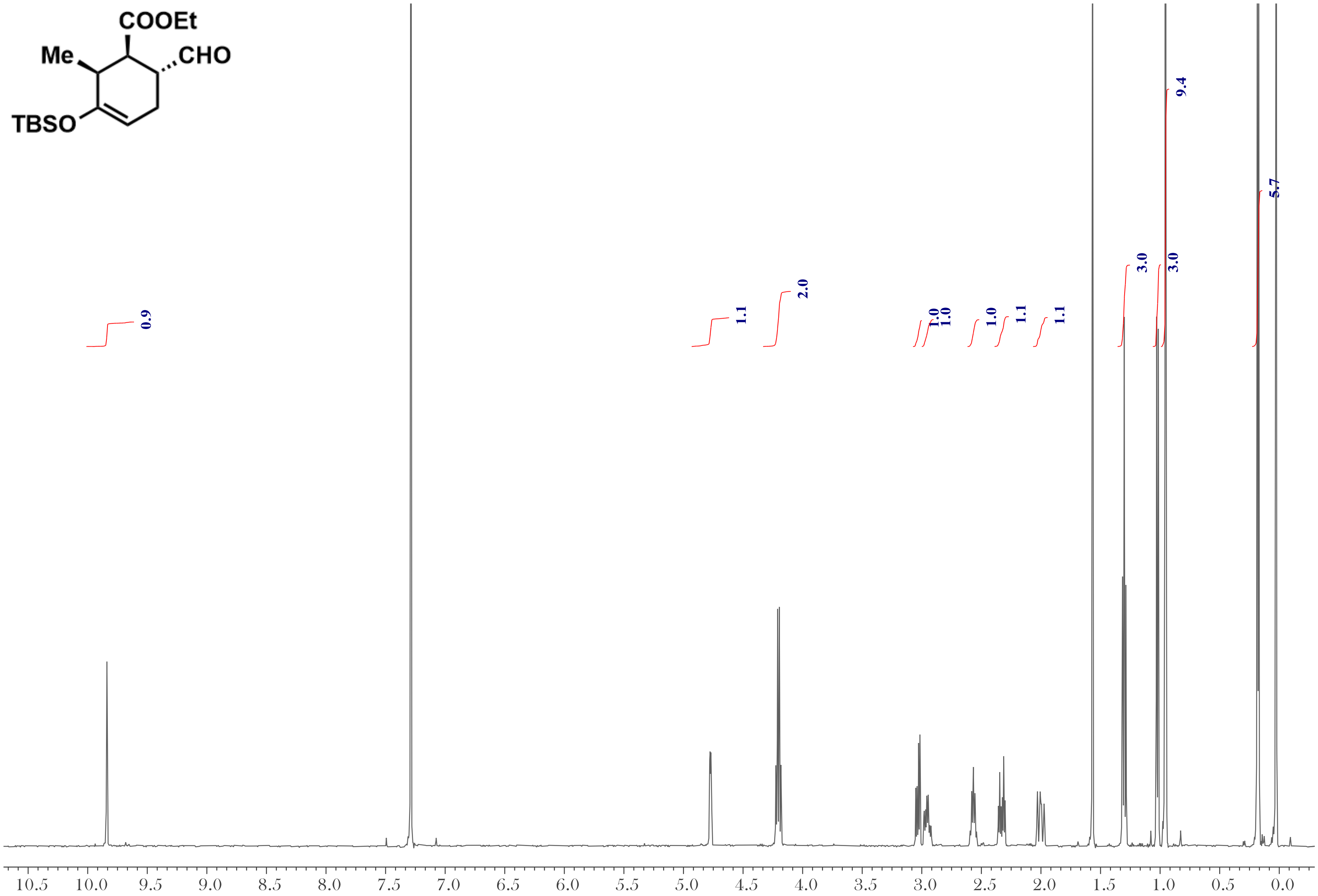

^{1}H NMR Spectrum of **S5** (400 MHz, $CDCl_3$, 25 °C)

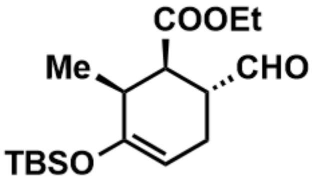

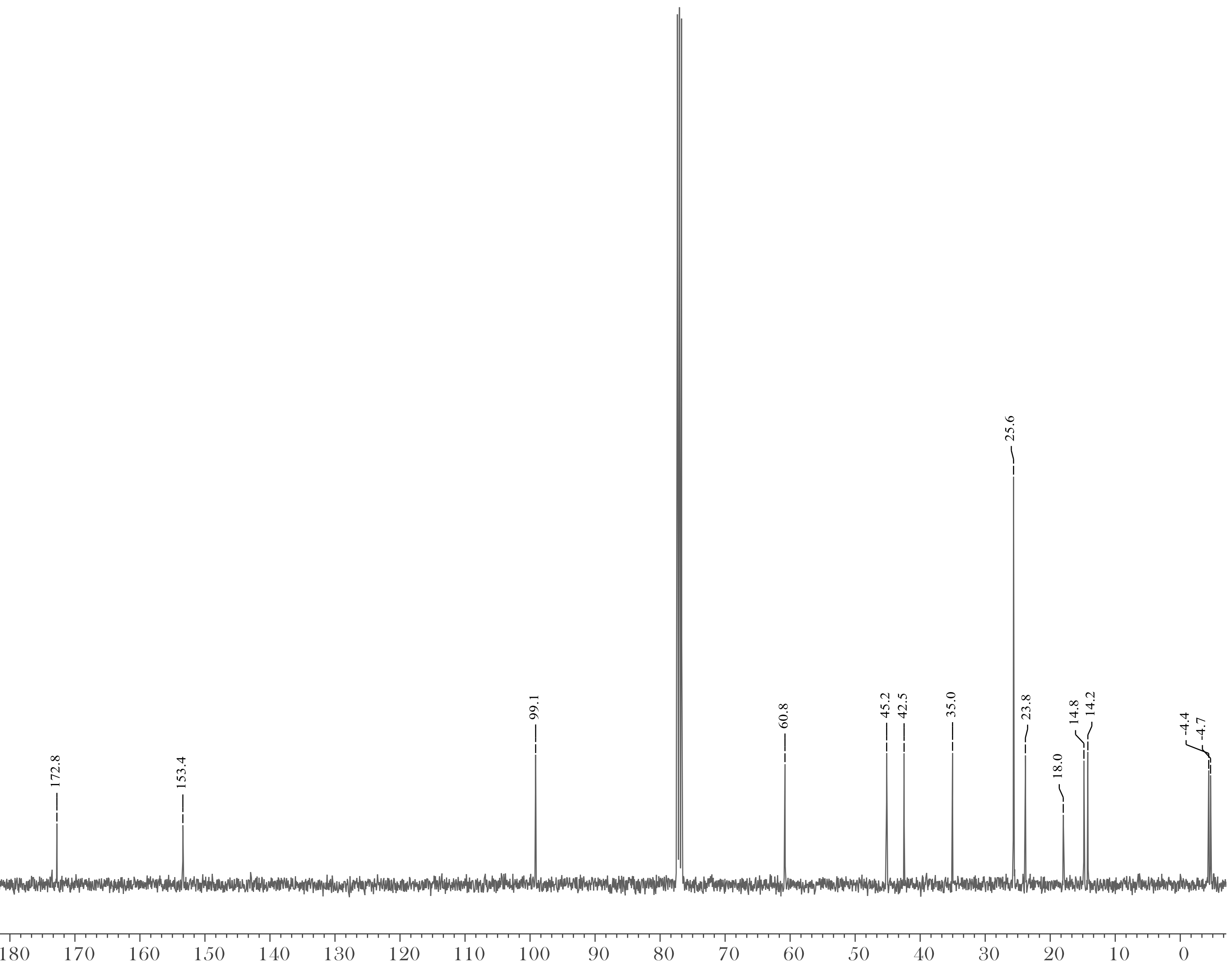

^{13}C NMR Spectrum of **S5** (100 MHz, $CDCl_3$, 25 °C)

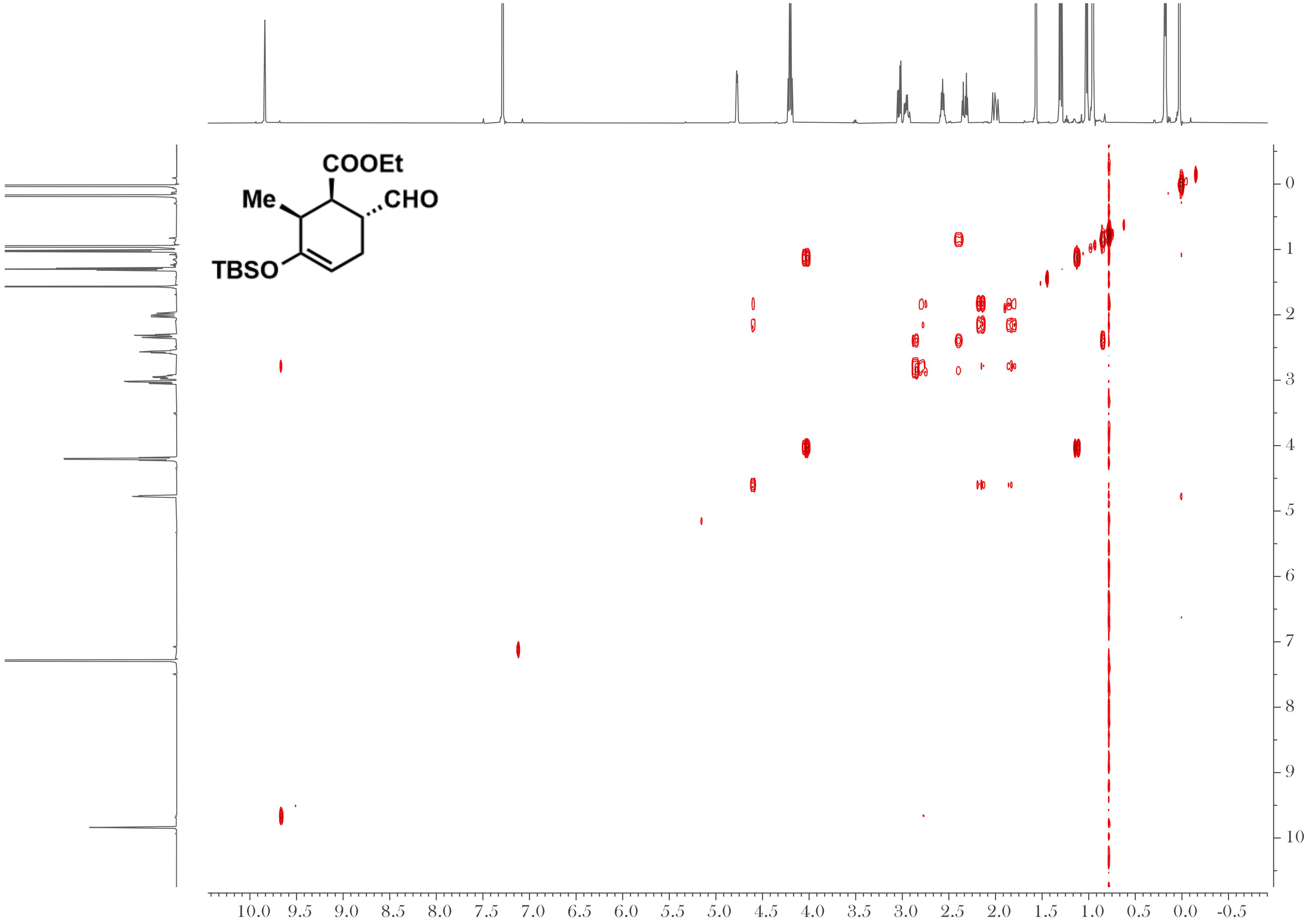

^{1}H–^{1}H COSY Spectrum of **S5** (400 MHz, $CDCl_3$, 25 °C)

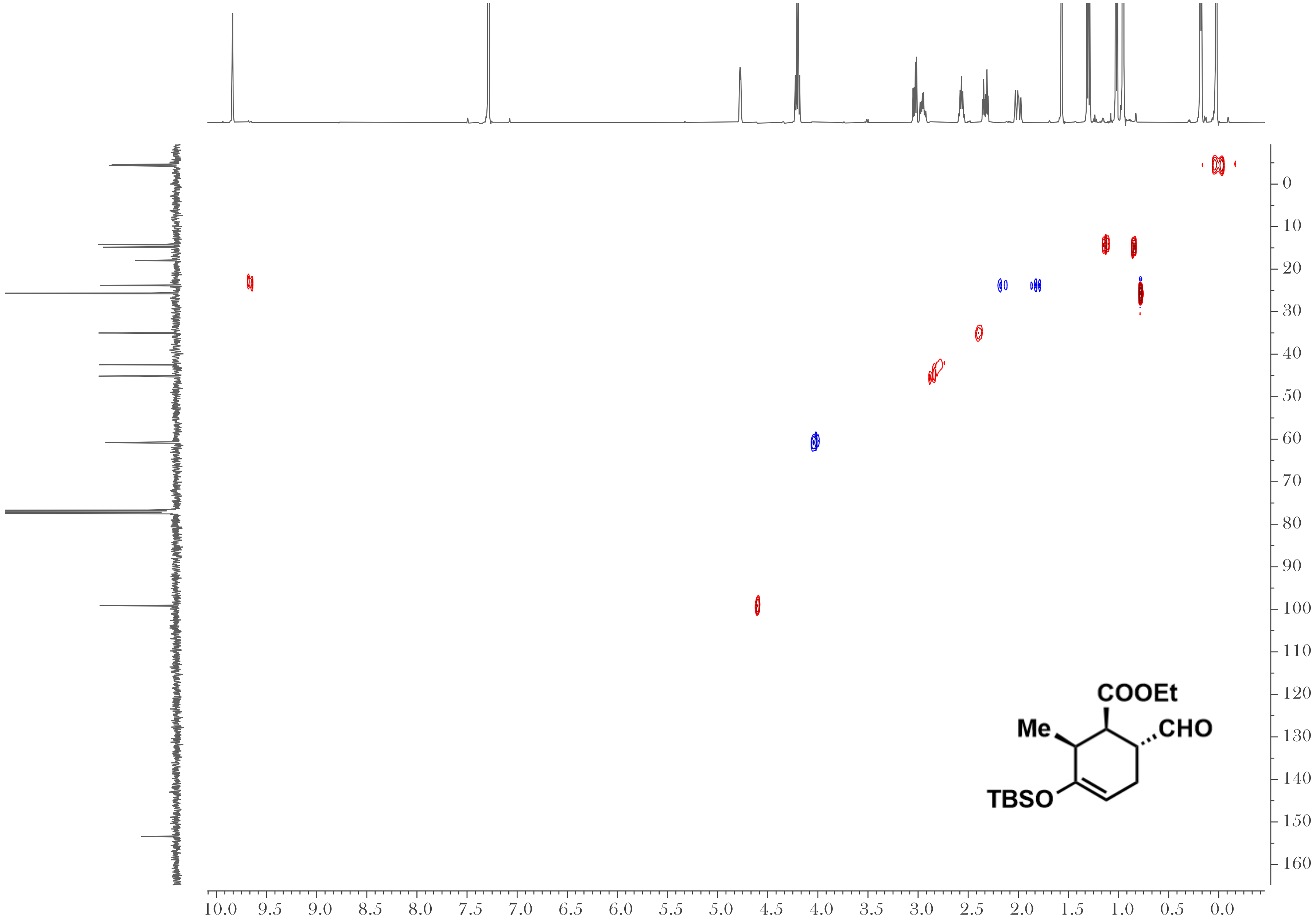

$^{1}H–^{13}C$ HSQC Spectrum of **S5** (100 MHz, $CDCl_3$, 25 °C)

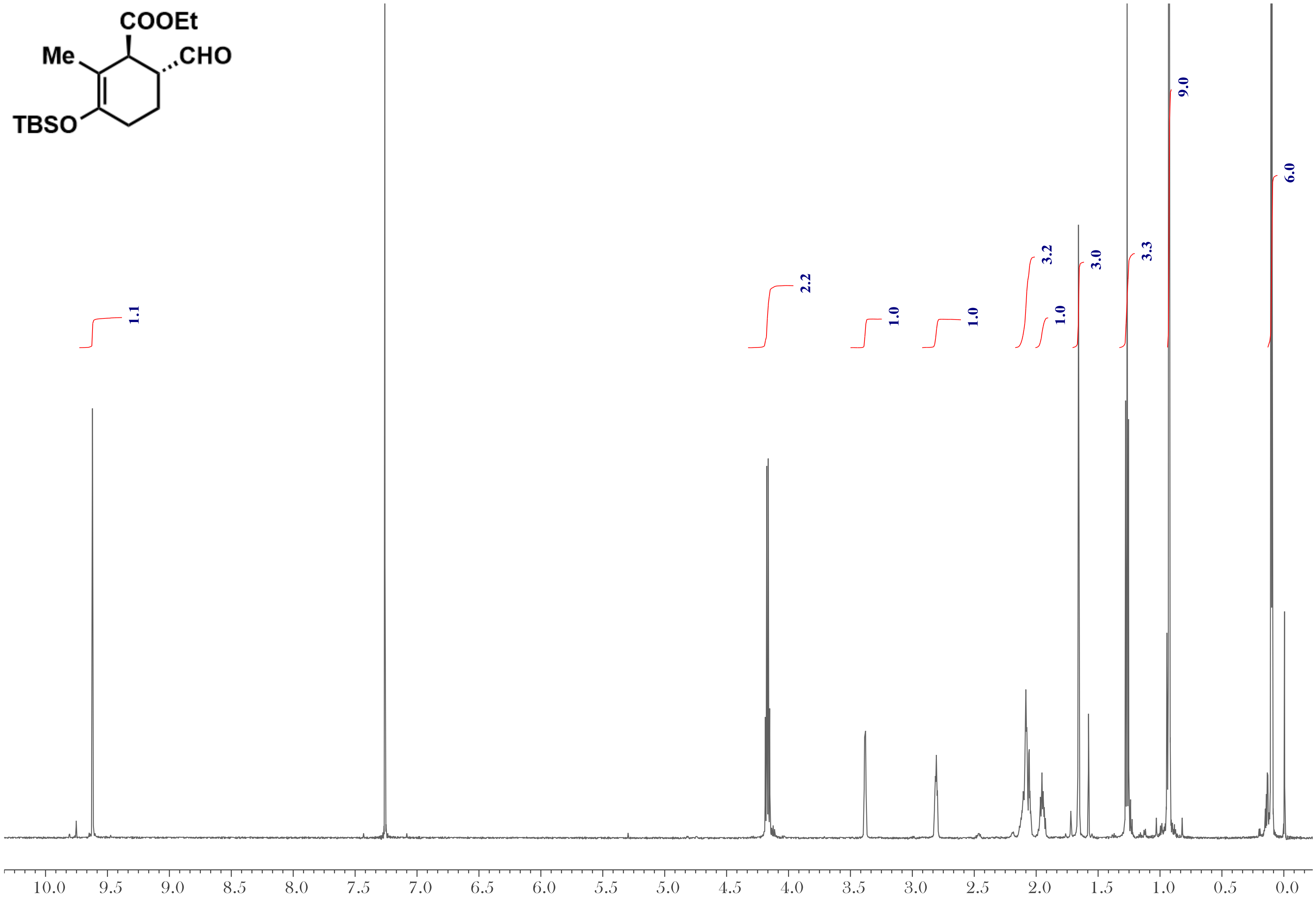

^{1}H NMR Spectrum of **S6** (600 MHz, $CDCl_3$, 25 °C)

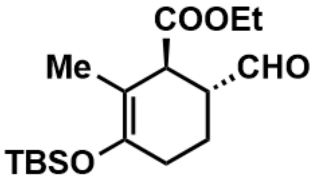

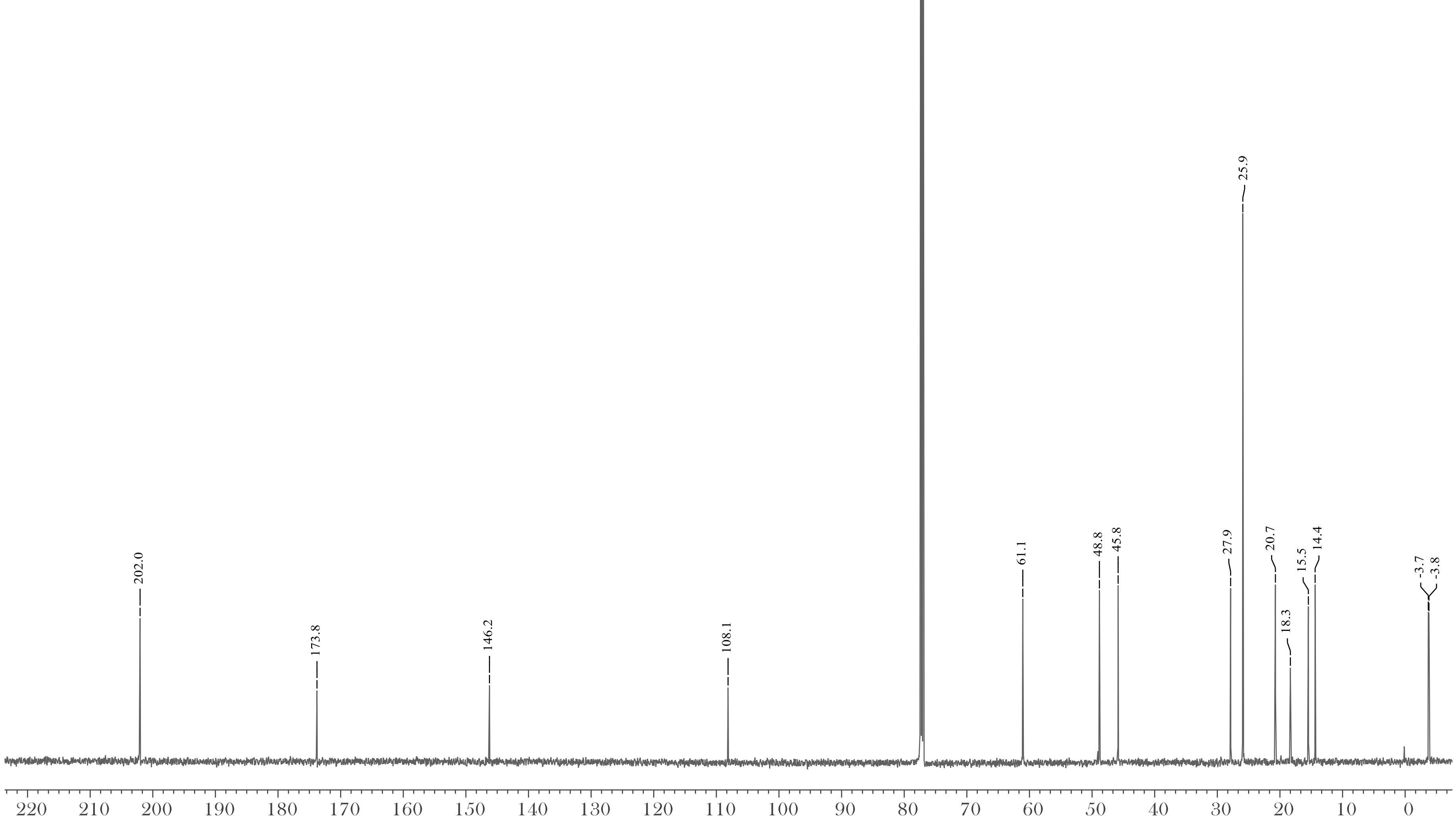

^{13}C NMR Spectrum of **S6** (150 MHz, $CDCl_3$, 25 °C)

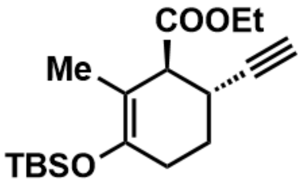

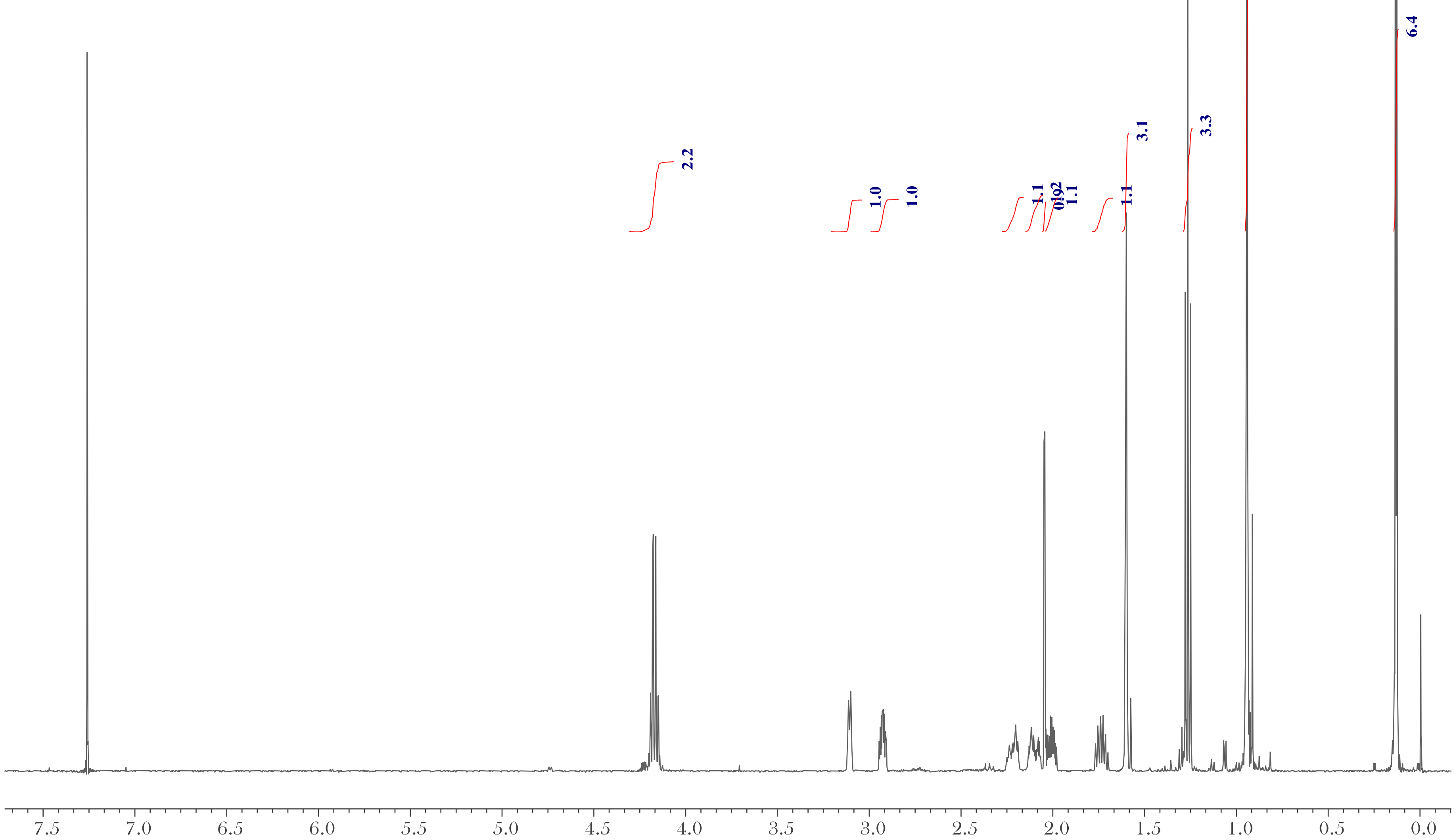

^{1}H NMR Spectrum of **S8** (500 MHz, $CDCl_3$, 25 °C)

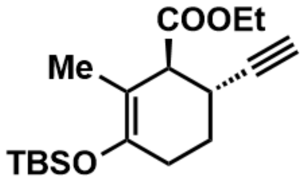

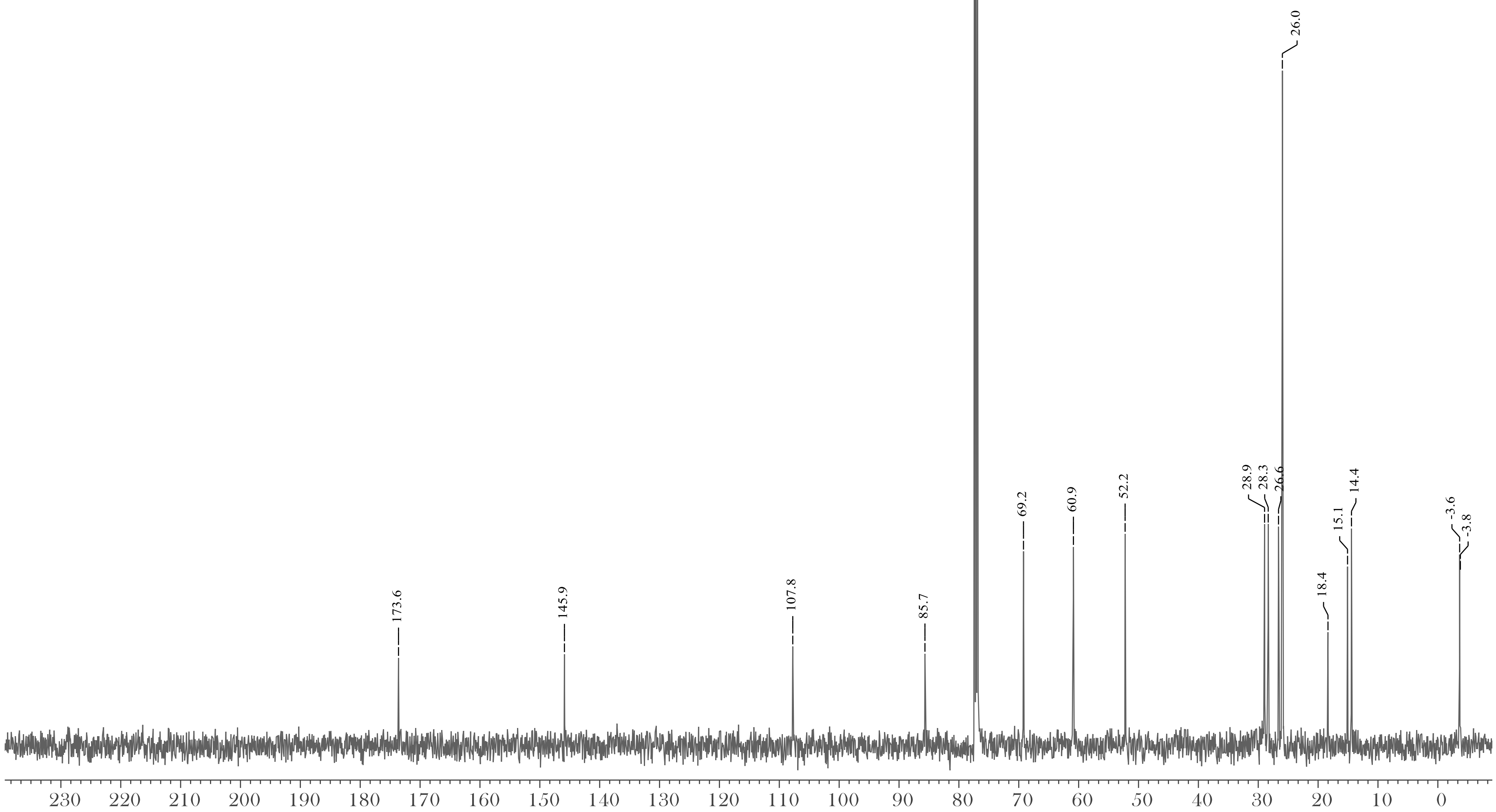

^{13}C NMR Spectrum of **S8** (125 MHz, $CDCl_3$, 25 °C)

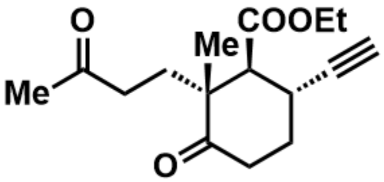

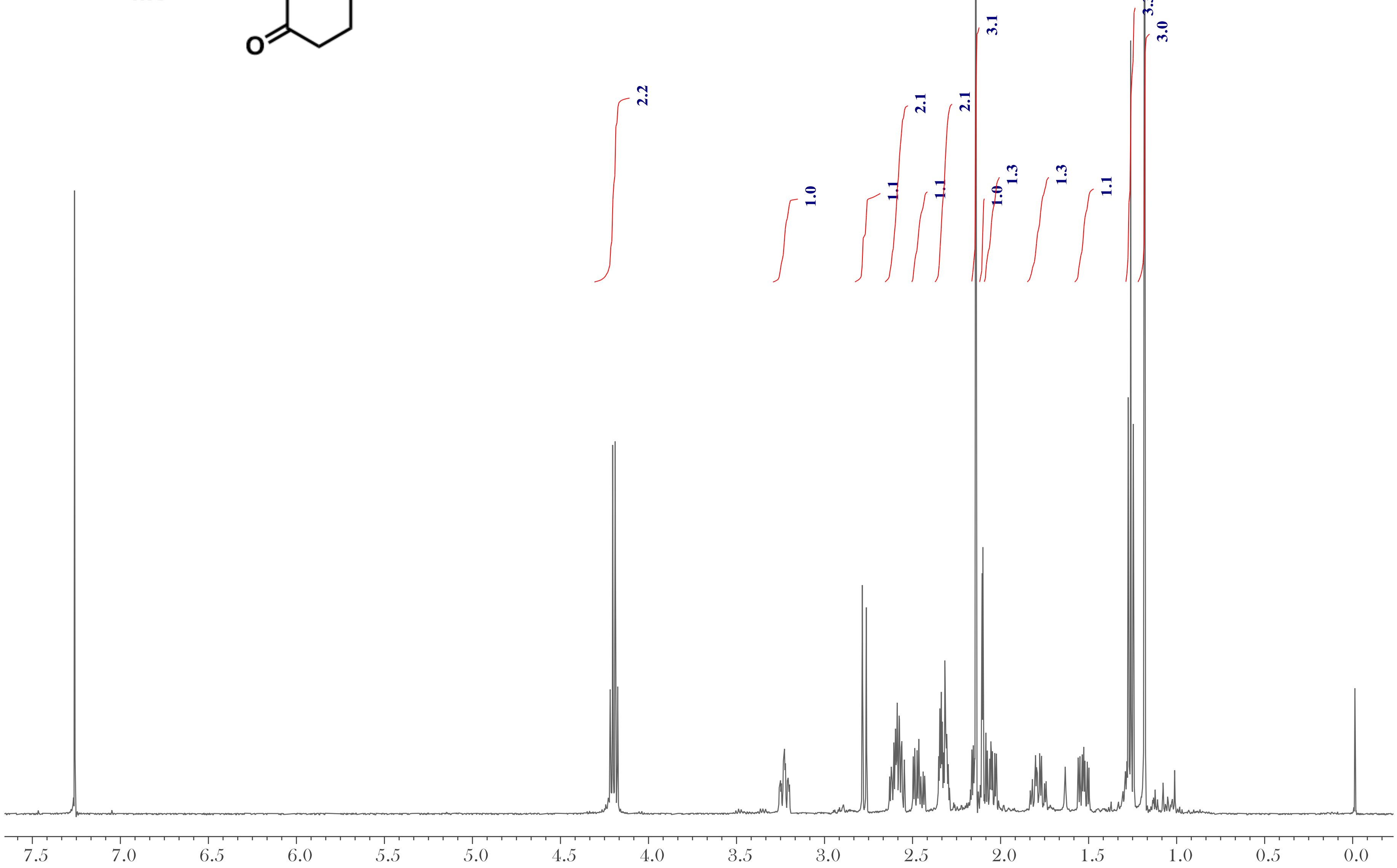

^{1}H NMR Spectrum of **S9** (500 MHz, $CDCl_3$, 25 °C)

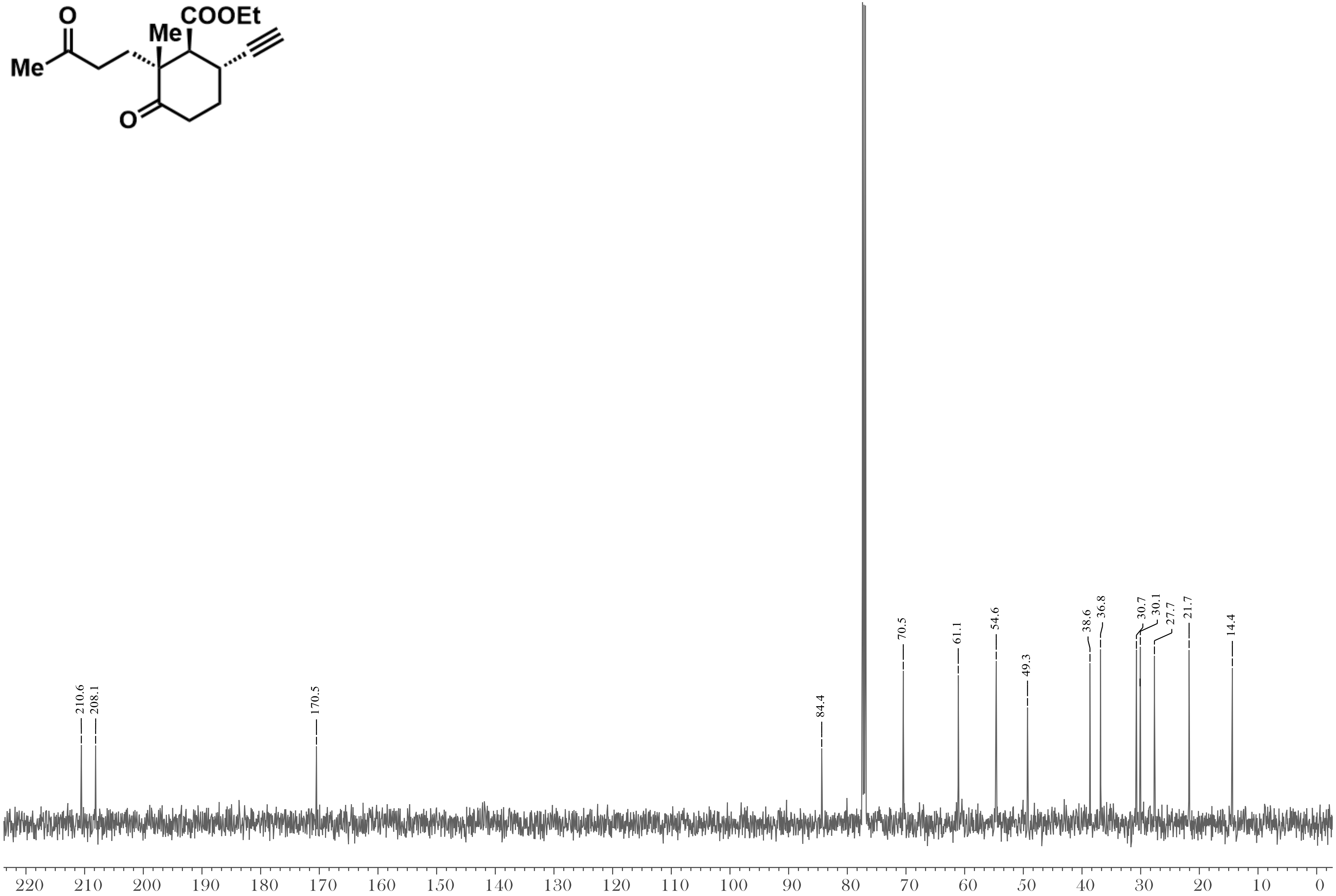

^{13}C NMR Spectrum of **S9** (125 MHz, $CDCl_3$, 25 °C)

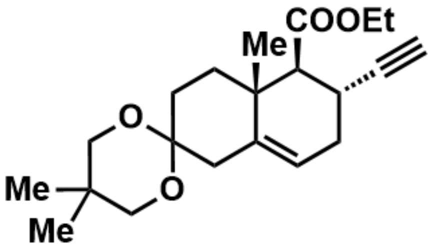

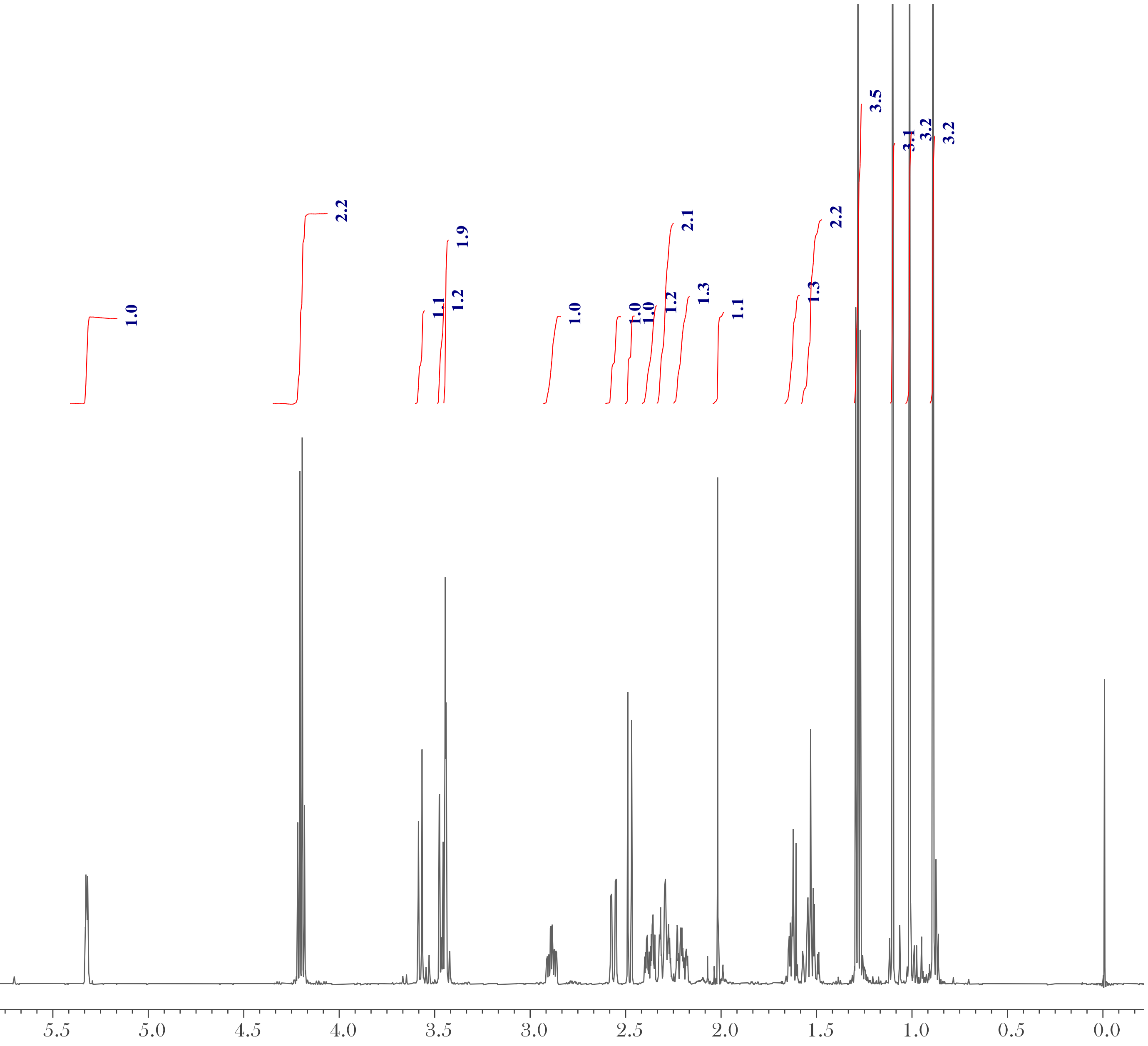

^{1}H NMR Spectrum of **S10** (600 MHz, $CDCl_3$, 25 °C)

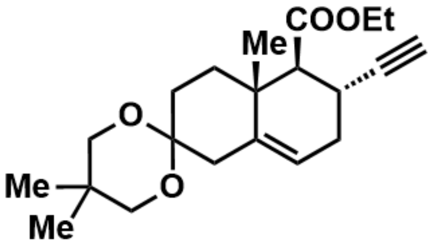

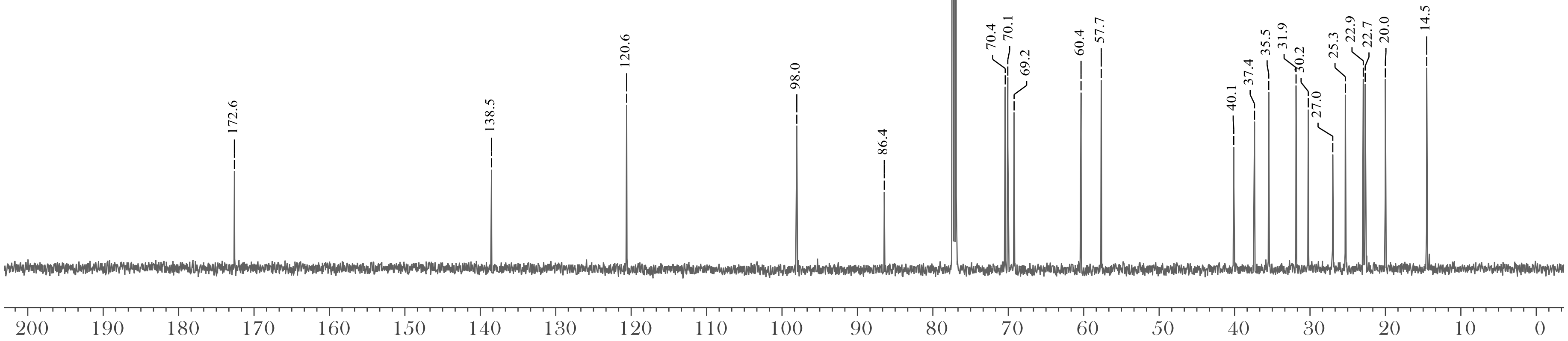

^{13}C NMR Spectrum of **S10** (150 MHz, $CDCl_3$, 25 °C)

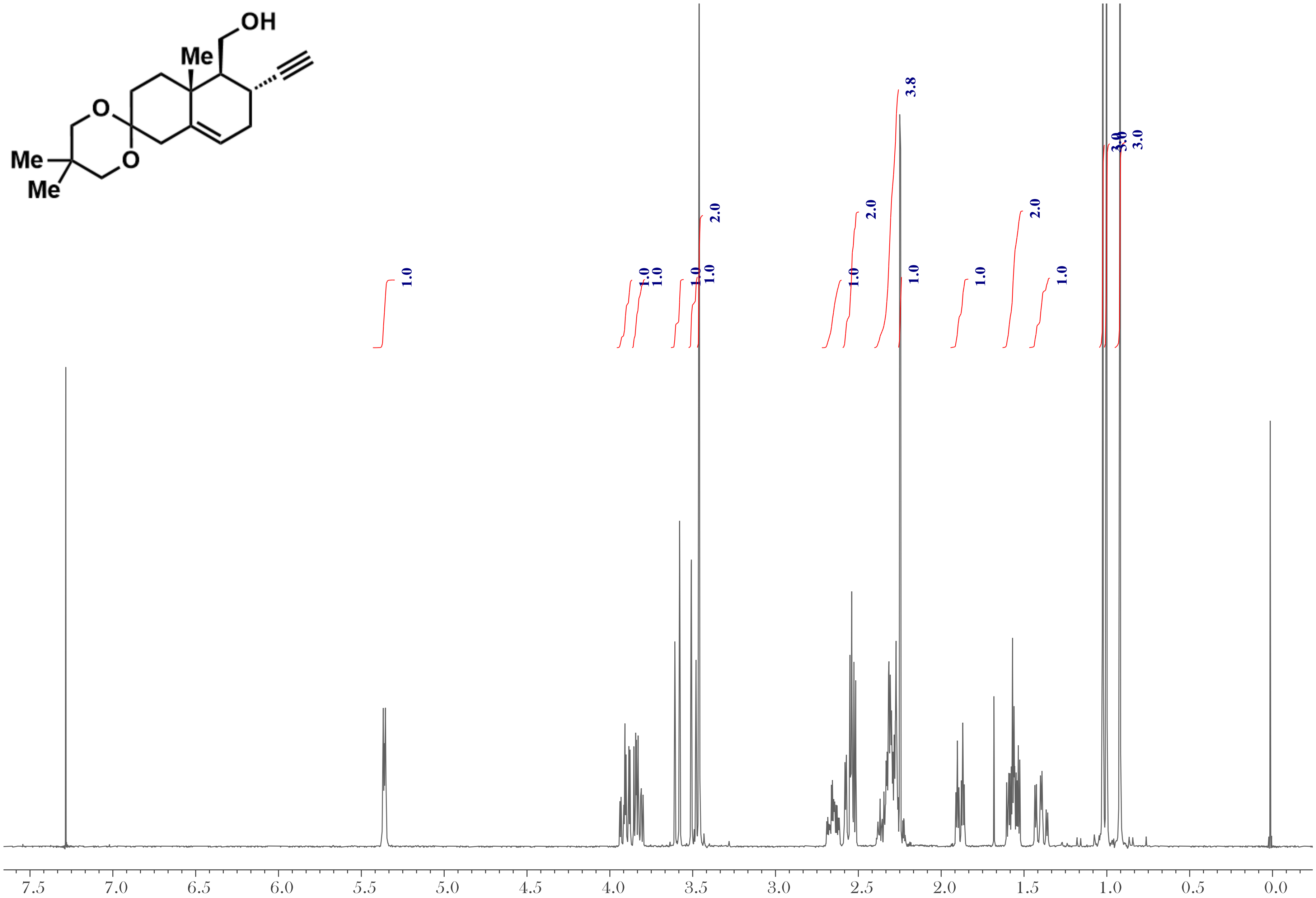

^{1}H NMR Spectrum of **S11** (400 MHz, $CDCl_3$, 25 °C)

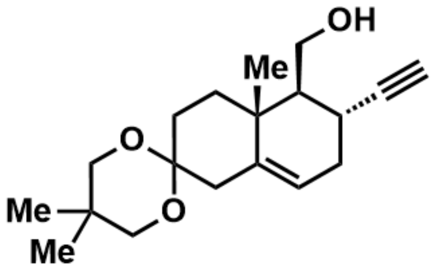

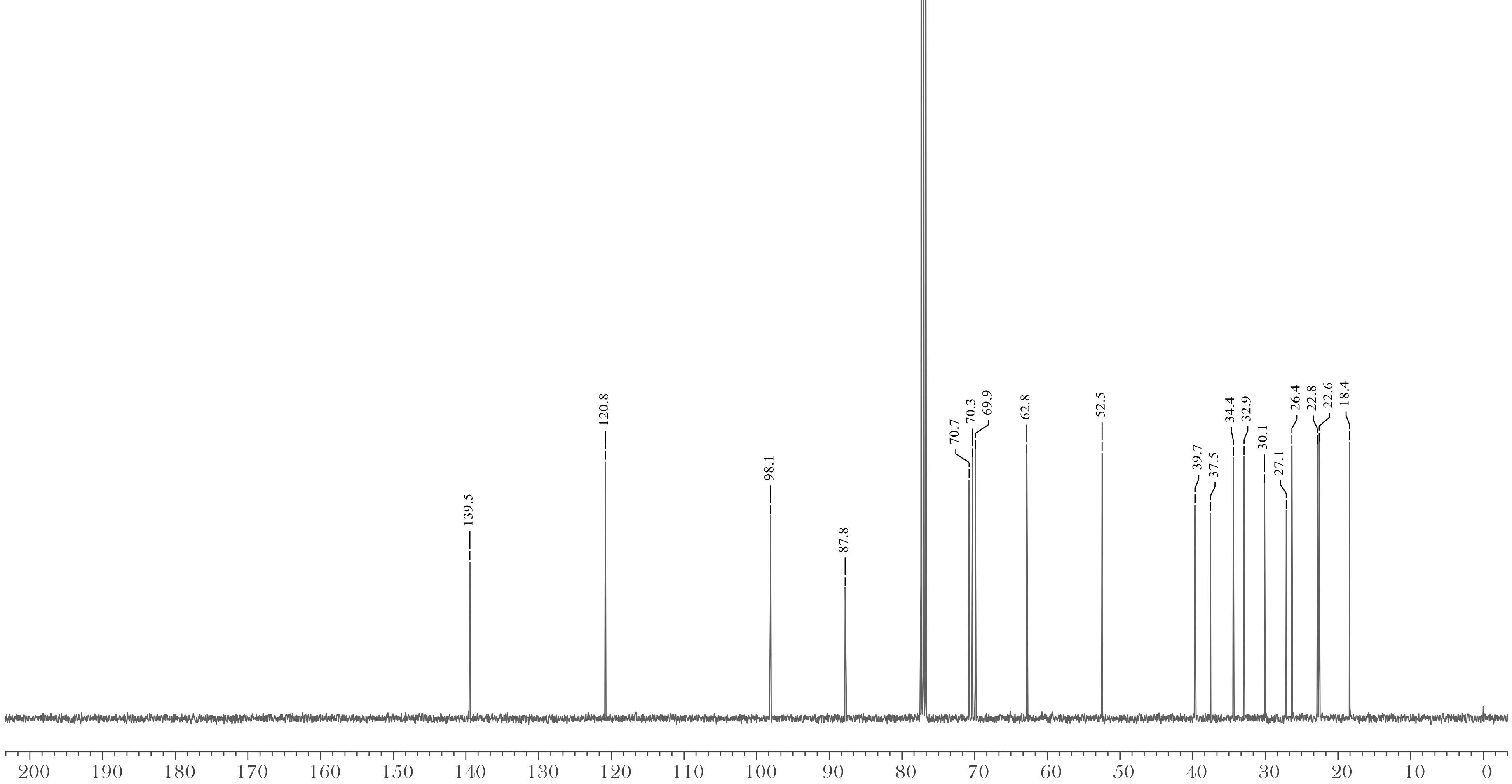

^{13}C NMR Spectrum of **S11** (100 MHz, $CDCl_3$, 25 °C)

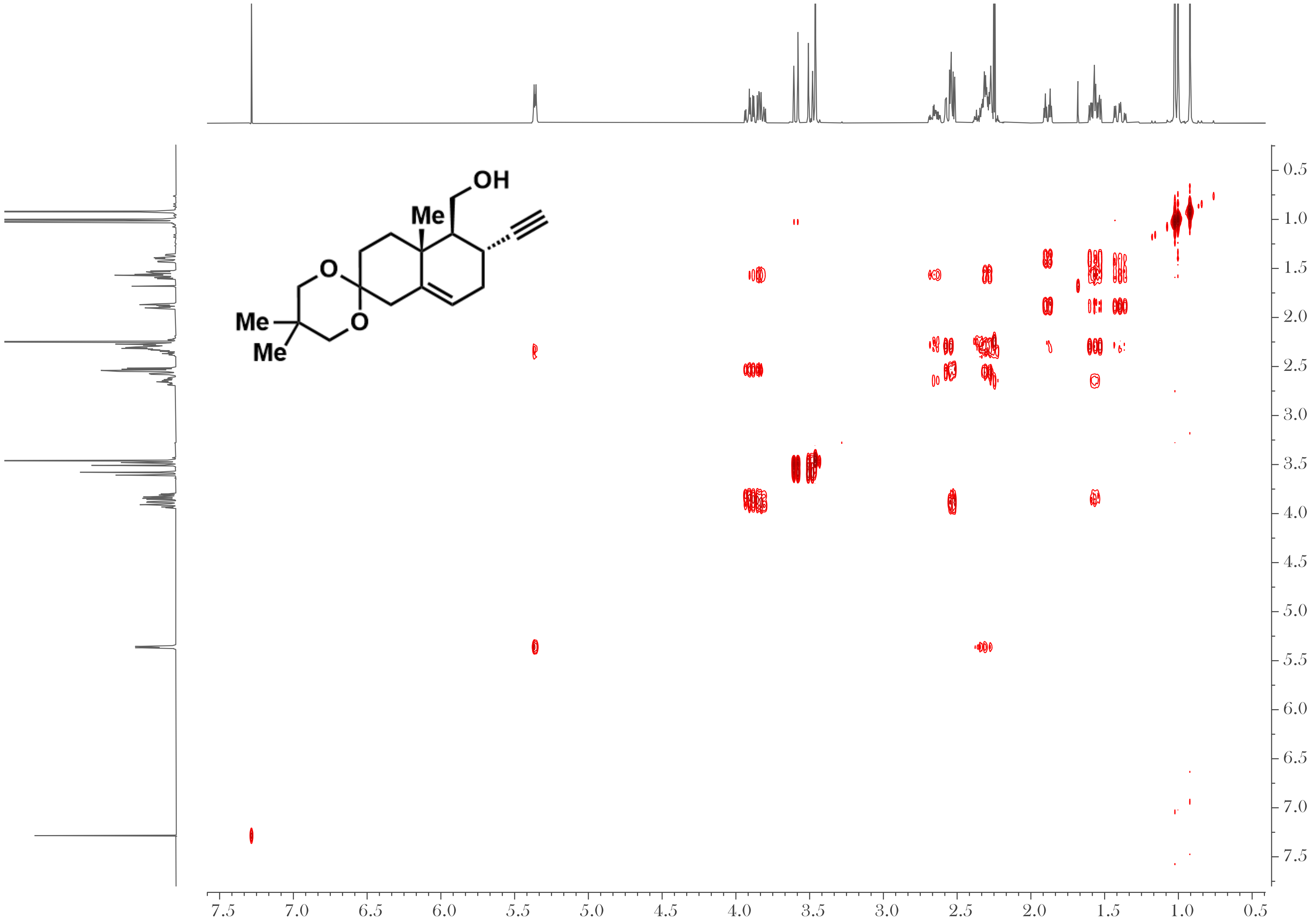

^{1}H–^{1}H COSY Spectrum of **S11** (400 MHz, $CDCl_3$, 25 °C)

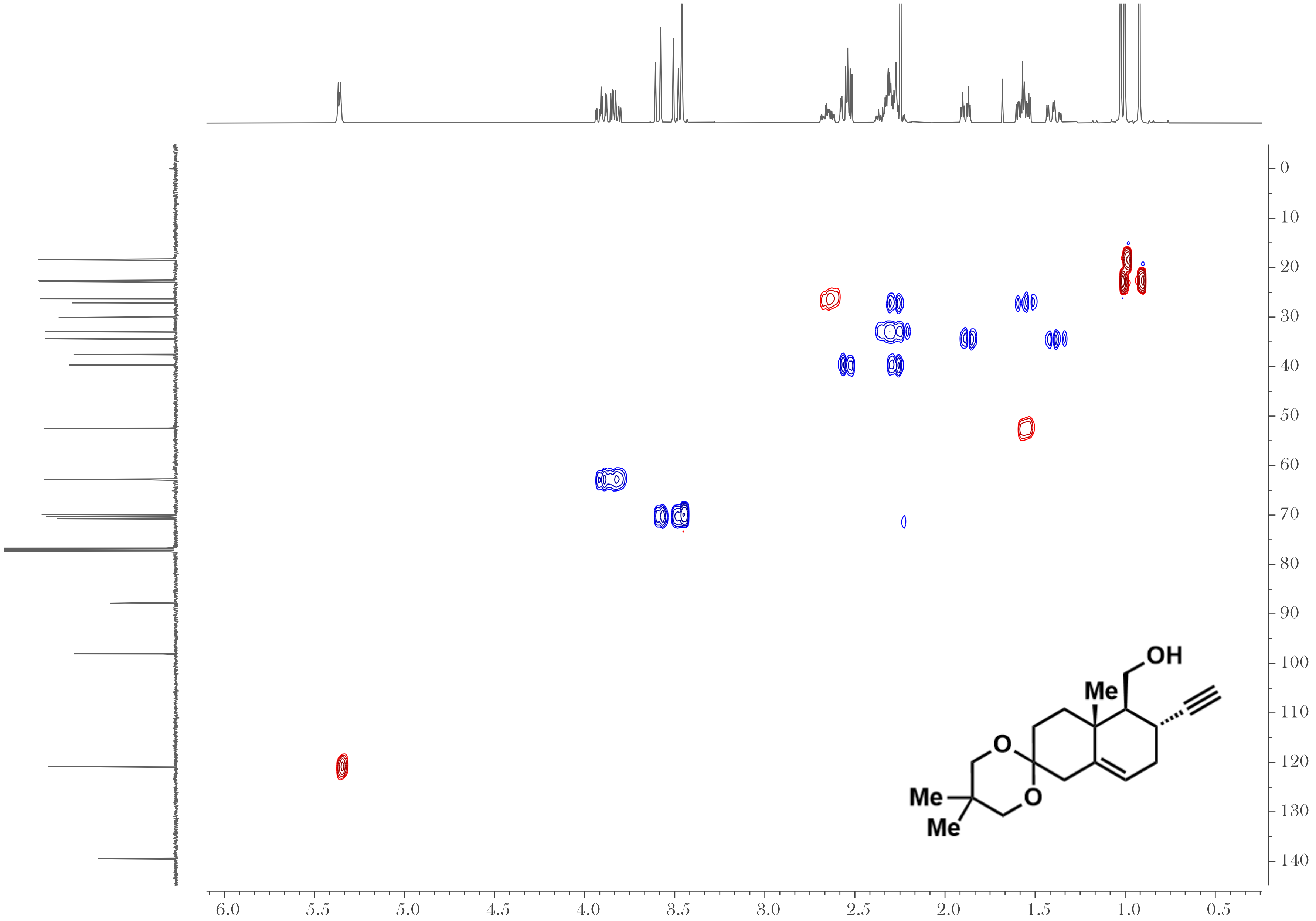

^{1}H–^{13}C HSQC Spectrum of **S11** (100 MHz, $CDCl_3$, 25 °C)

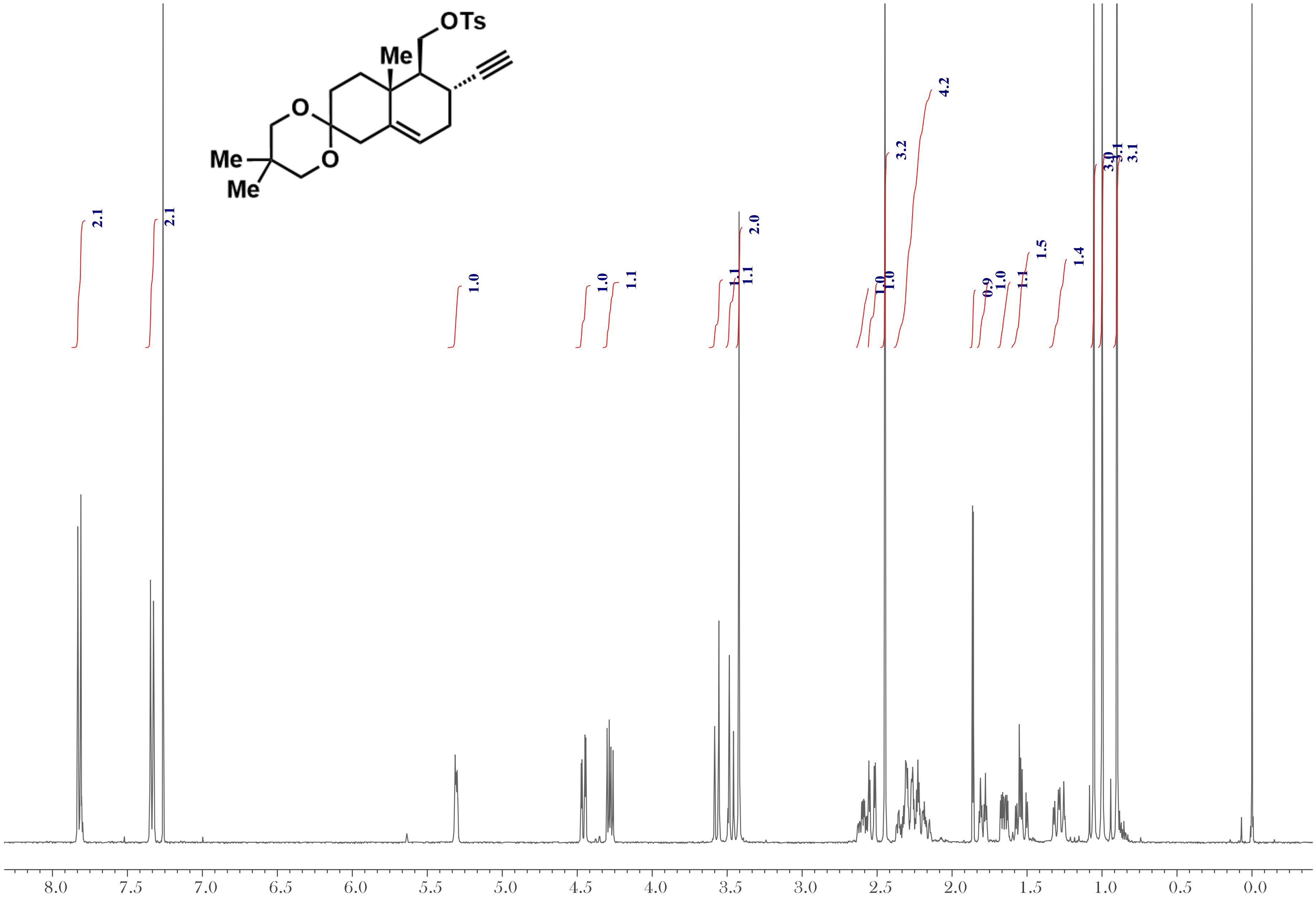

^{1}H NMR Spectrum of **S12** (400 MHz, $CDCl_3$, 25 °C)

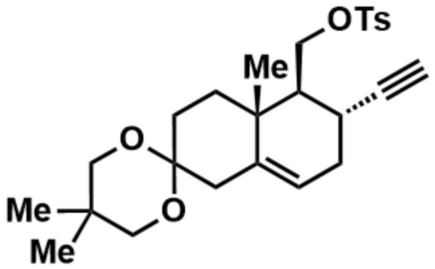

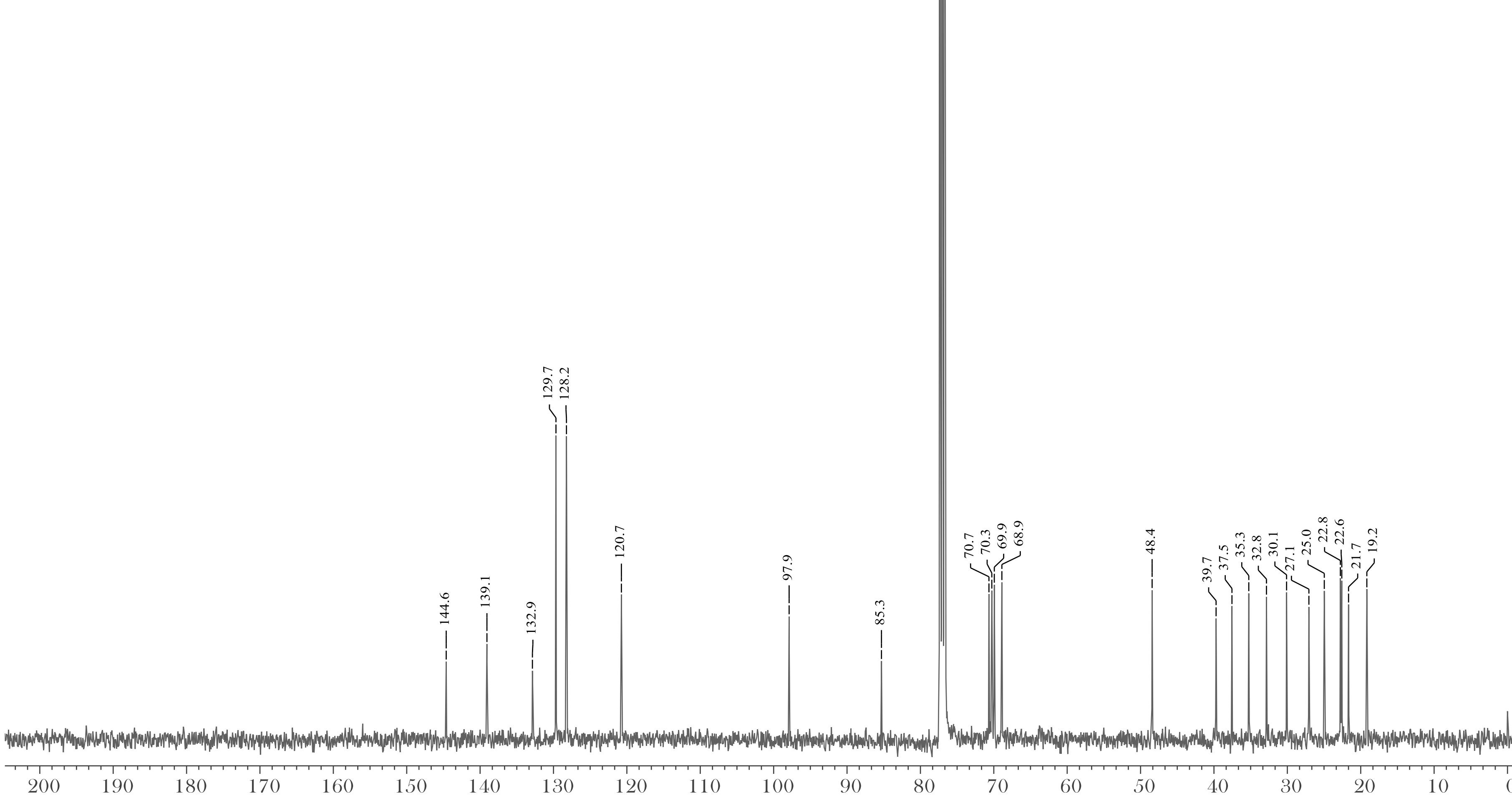

^{13}C NMR Spectrum of **S12** (100 MHz, $CDCl_3$, 25 °C)

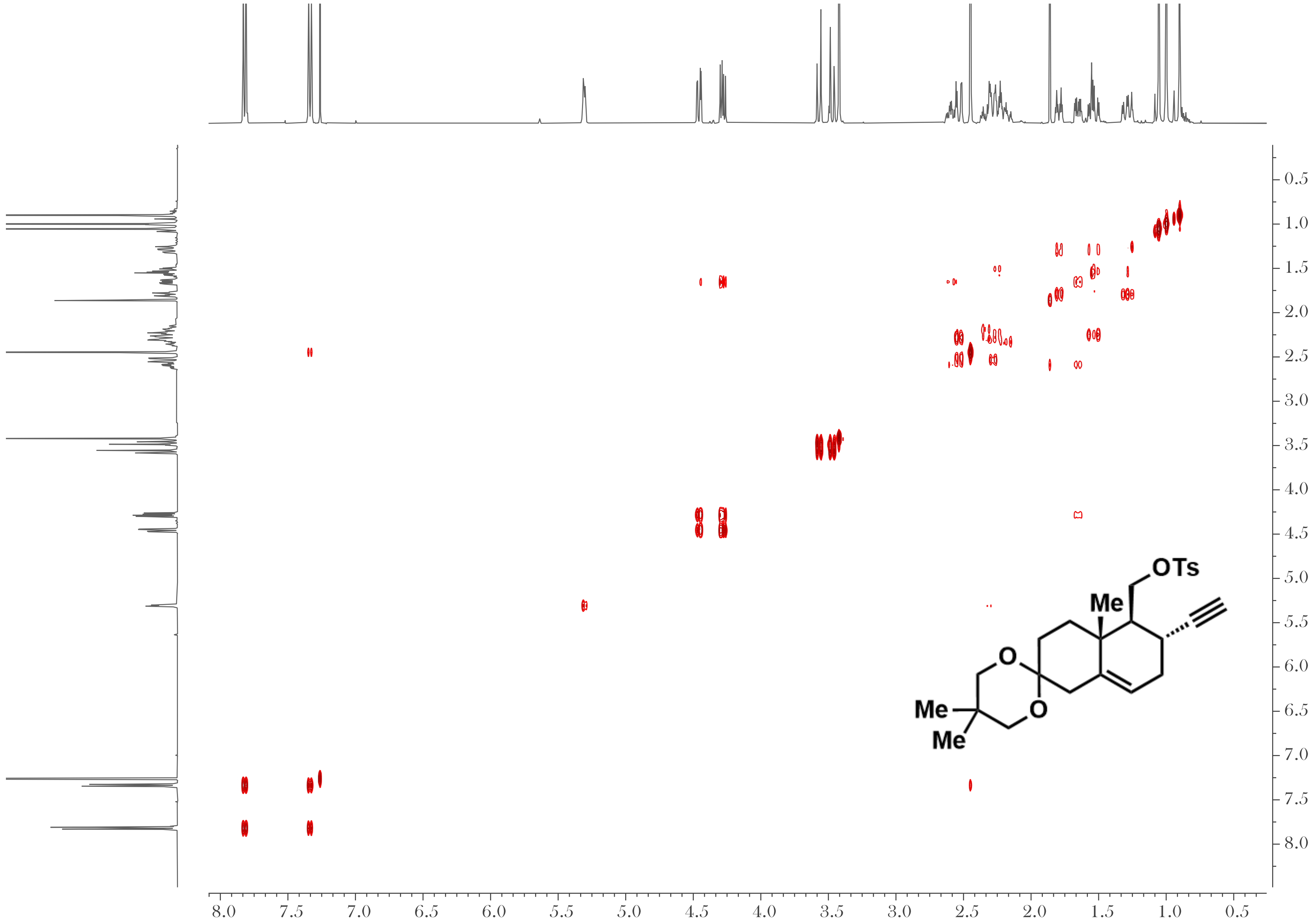

^1H–^1H COSY Spectrum of **S12** (400 MHz, $CDCl_3$, 25 °C)

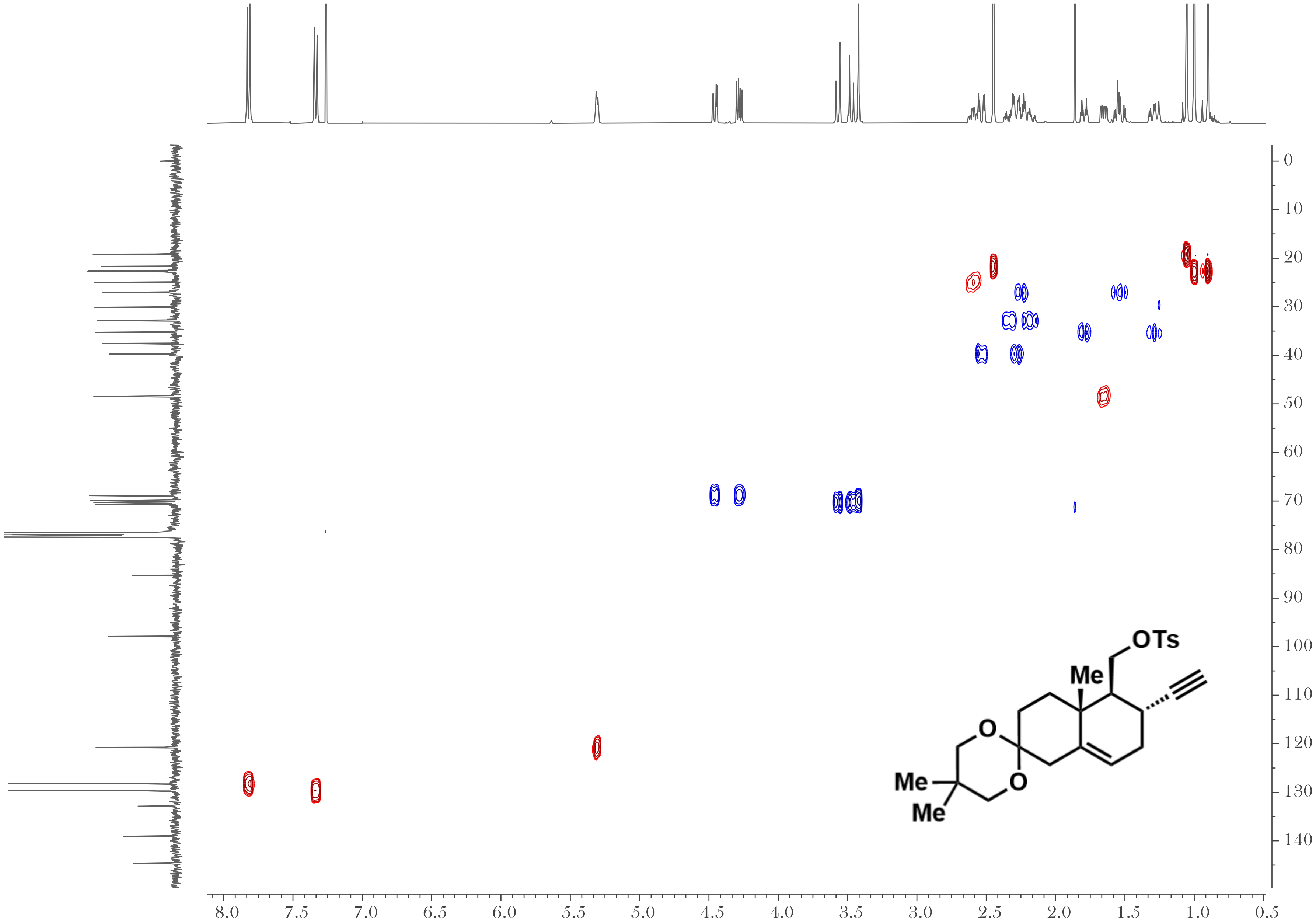

^1H–^{13}C HSQC Spectrum of **S12** (100 MHz, $CDCl_3$, 25 °C)

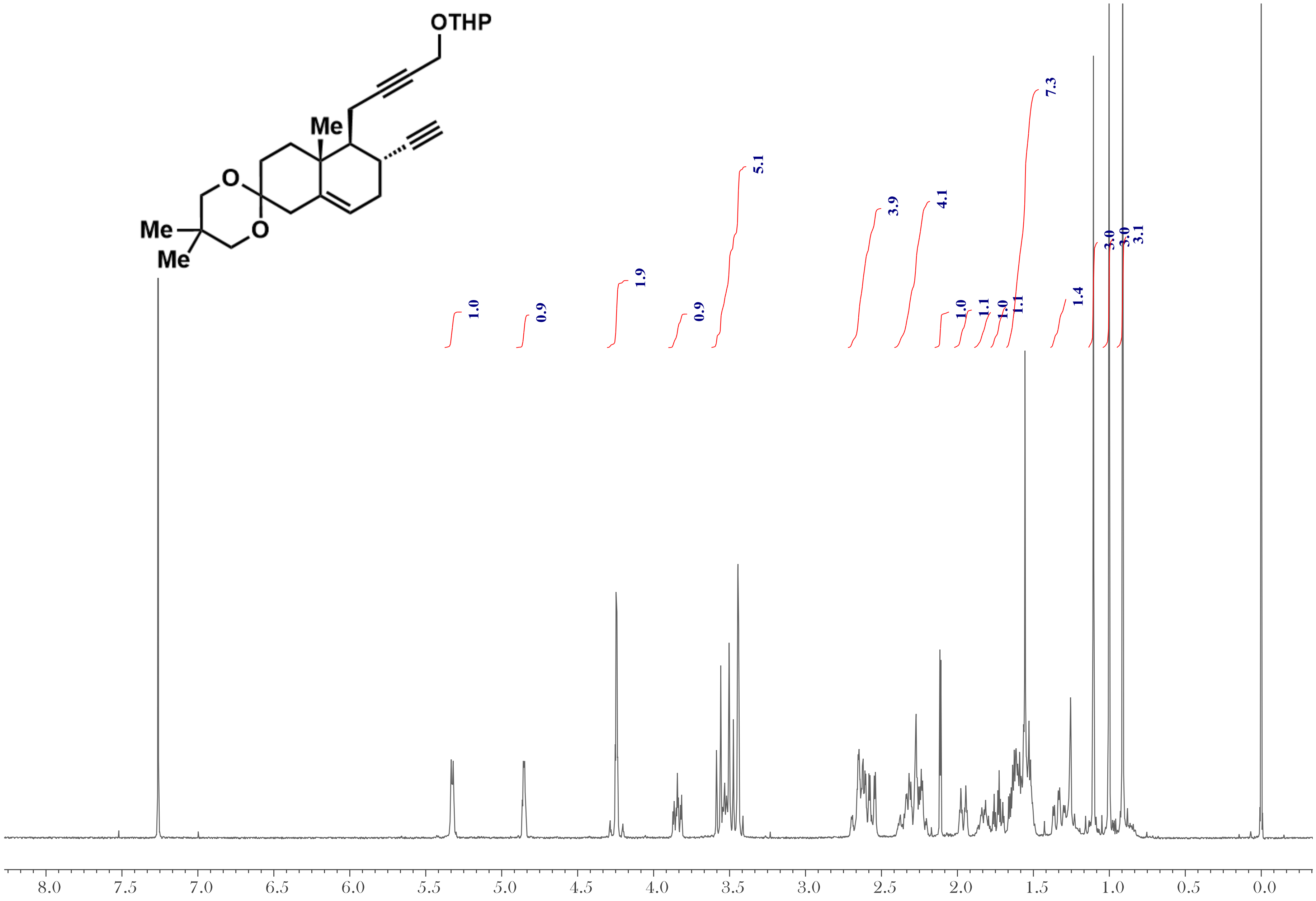

^{1}H NMR Spectrum of **12** (400 MHz, $CDCl_3$, 25 °C)

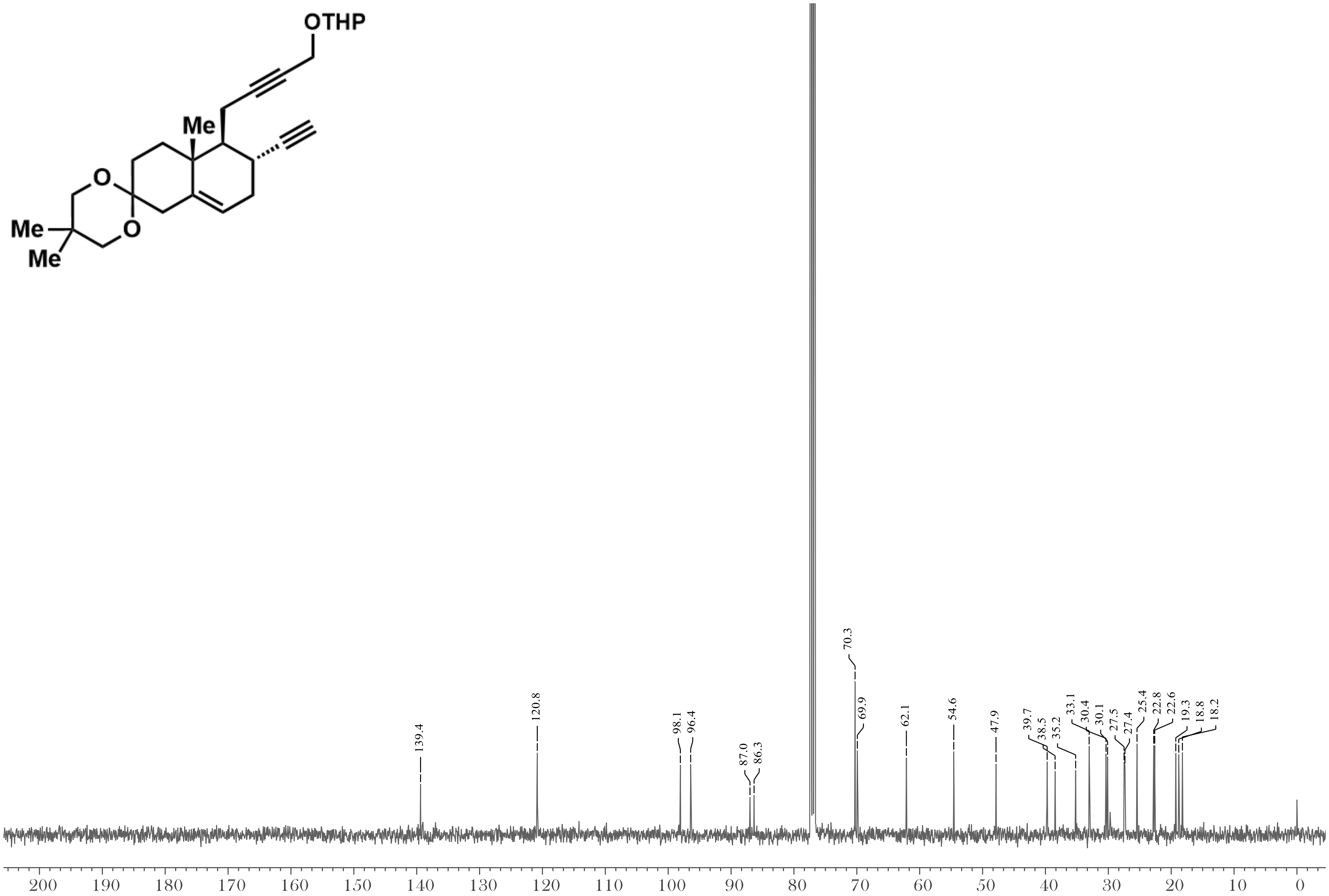

^{13}C NMR Spectrum of **12** (100 MHz, $CDCl_3$, 25 °C)

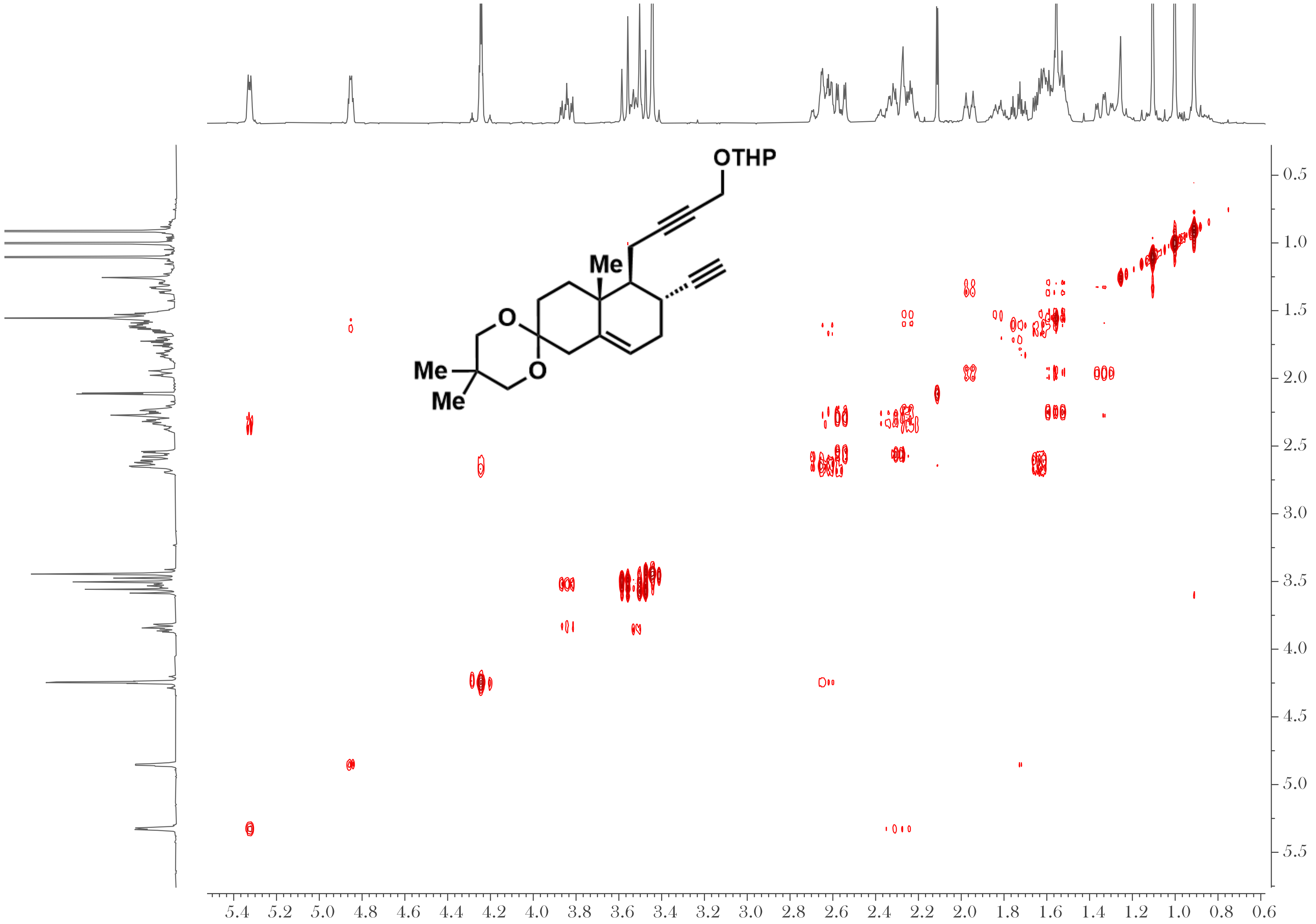

^1H–^1H COSY Spectrum of **12** (400 MHz, $CDCl_3$, 25 °C)

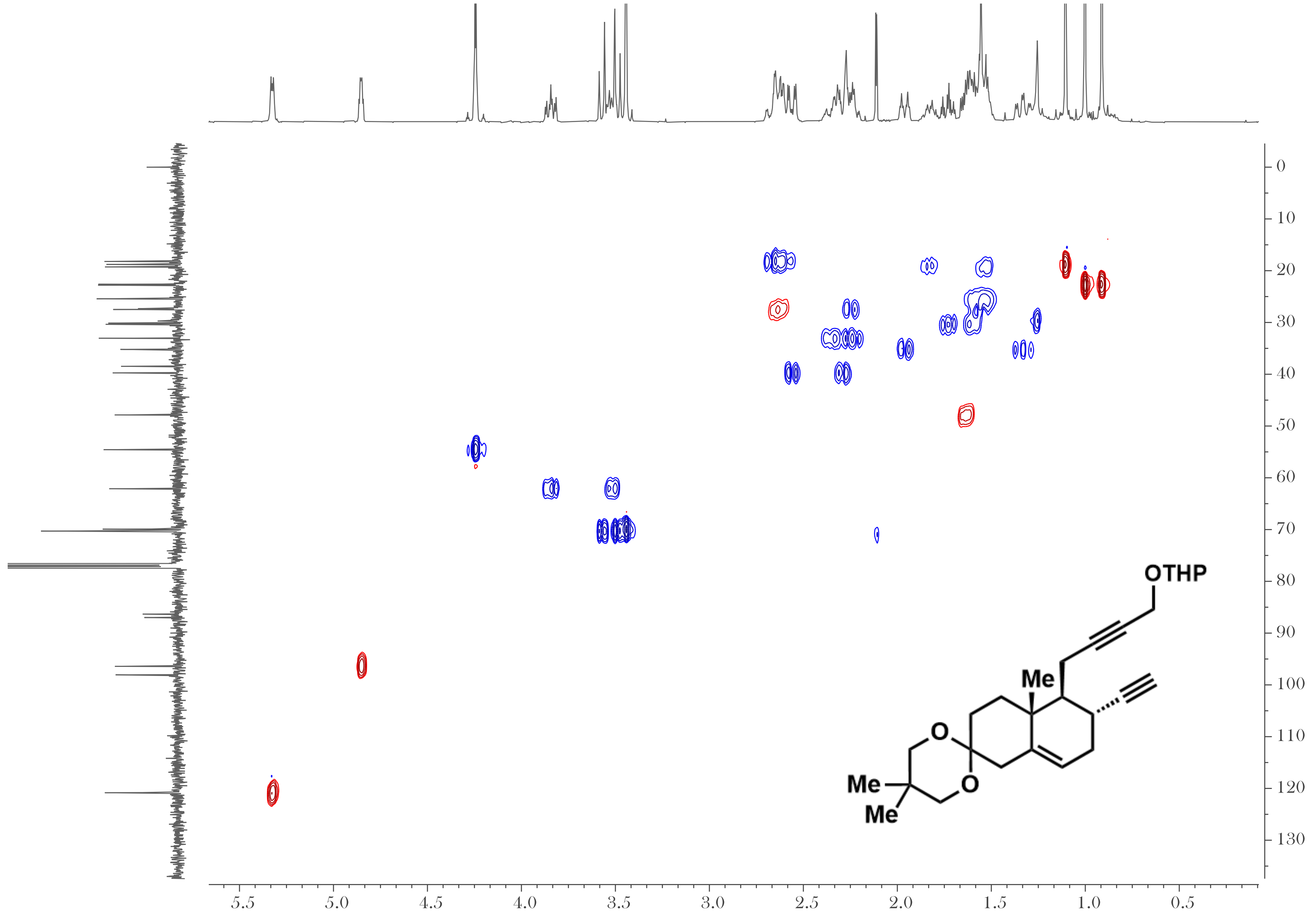

^{1}H–^{13}C HSQC Spectrum of **12** (100 MHz, $CDCl_3$, 25 °C)

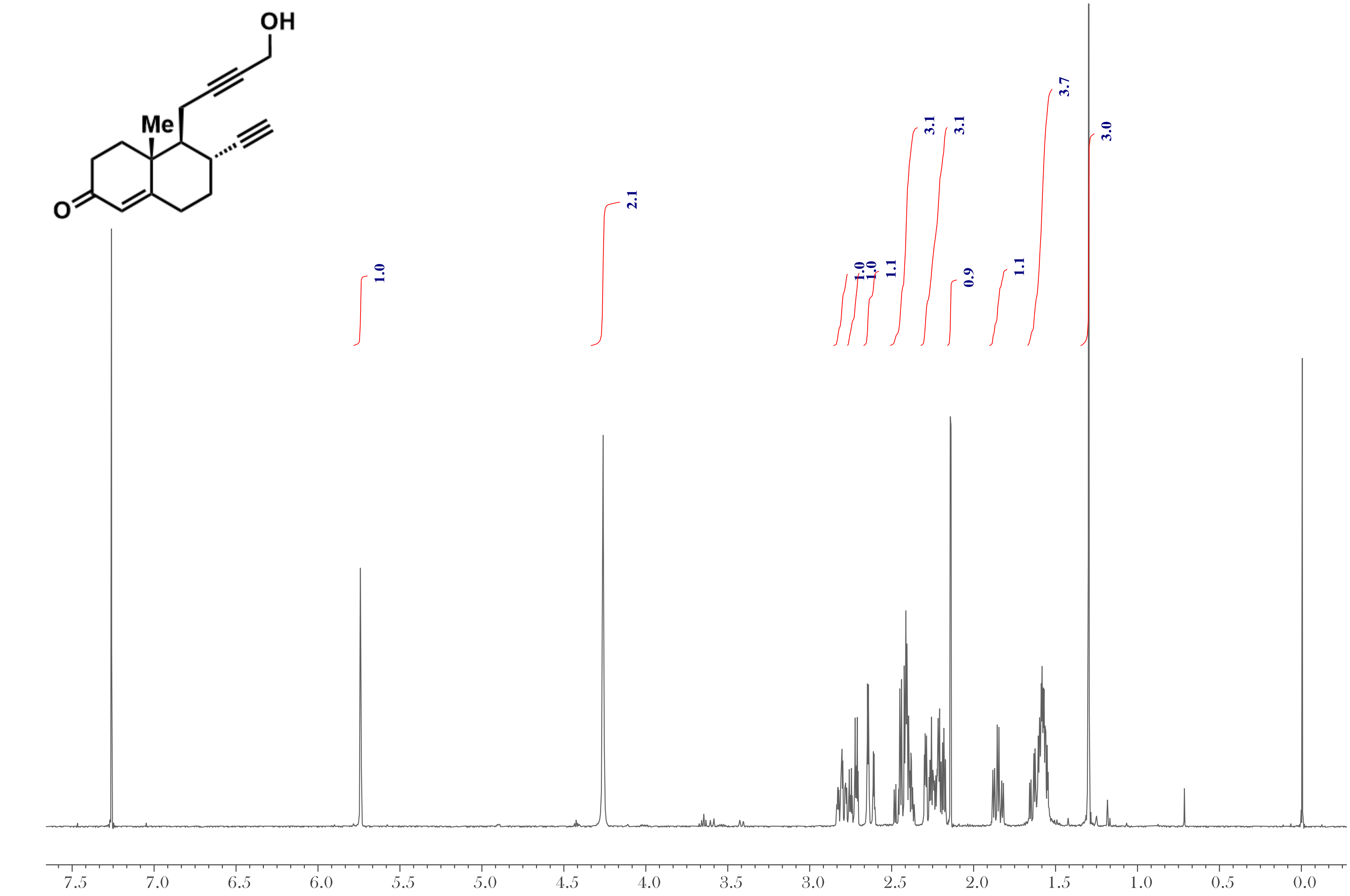

^{1}H NMR Spectrum of **13** (500 MHz, $CDCl_3$, 25 °C)

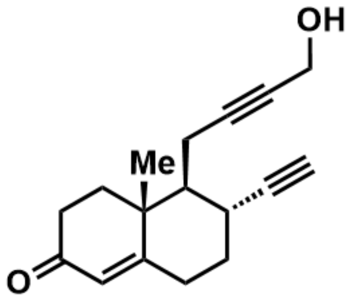

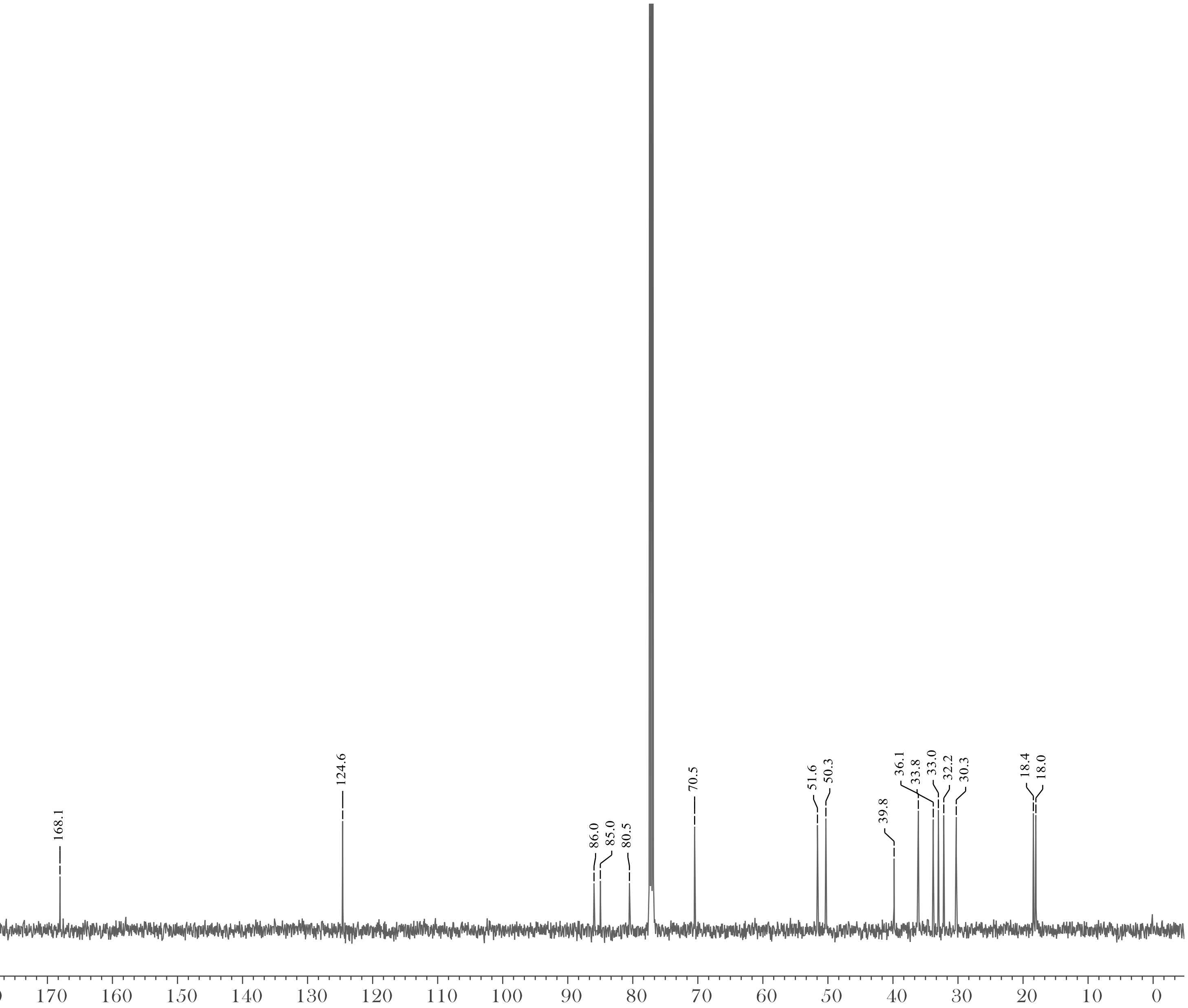

^{13}C NMR Spectrum of **13** (125 MHz, $CDCl_3$, 25 °C)

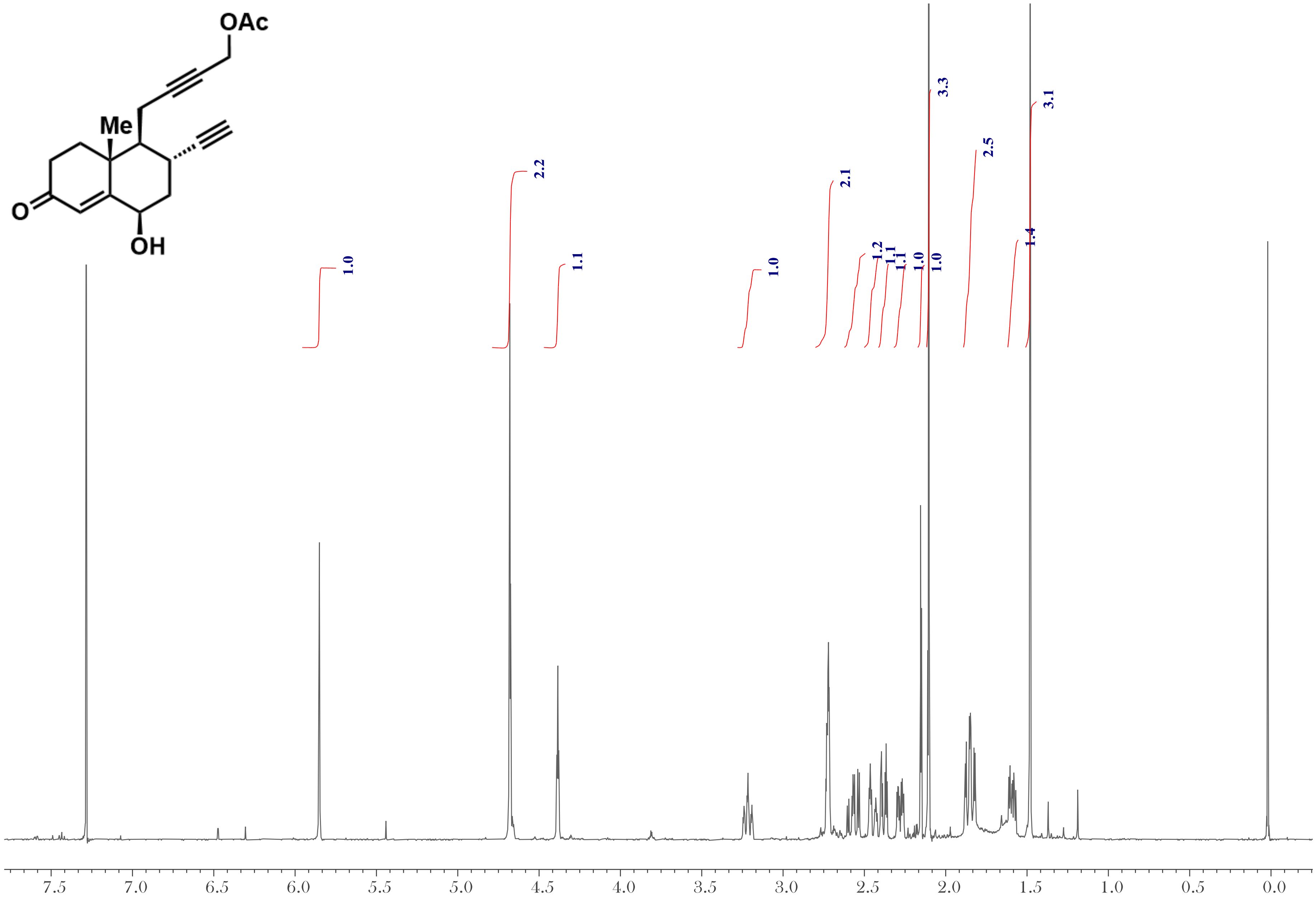

^{1}H NMR Spectrum of **14** (500 MHz, $CDCl_3$, 25 °C)

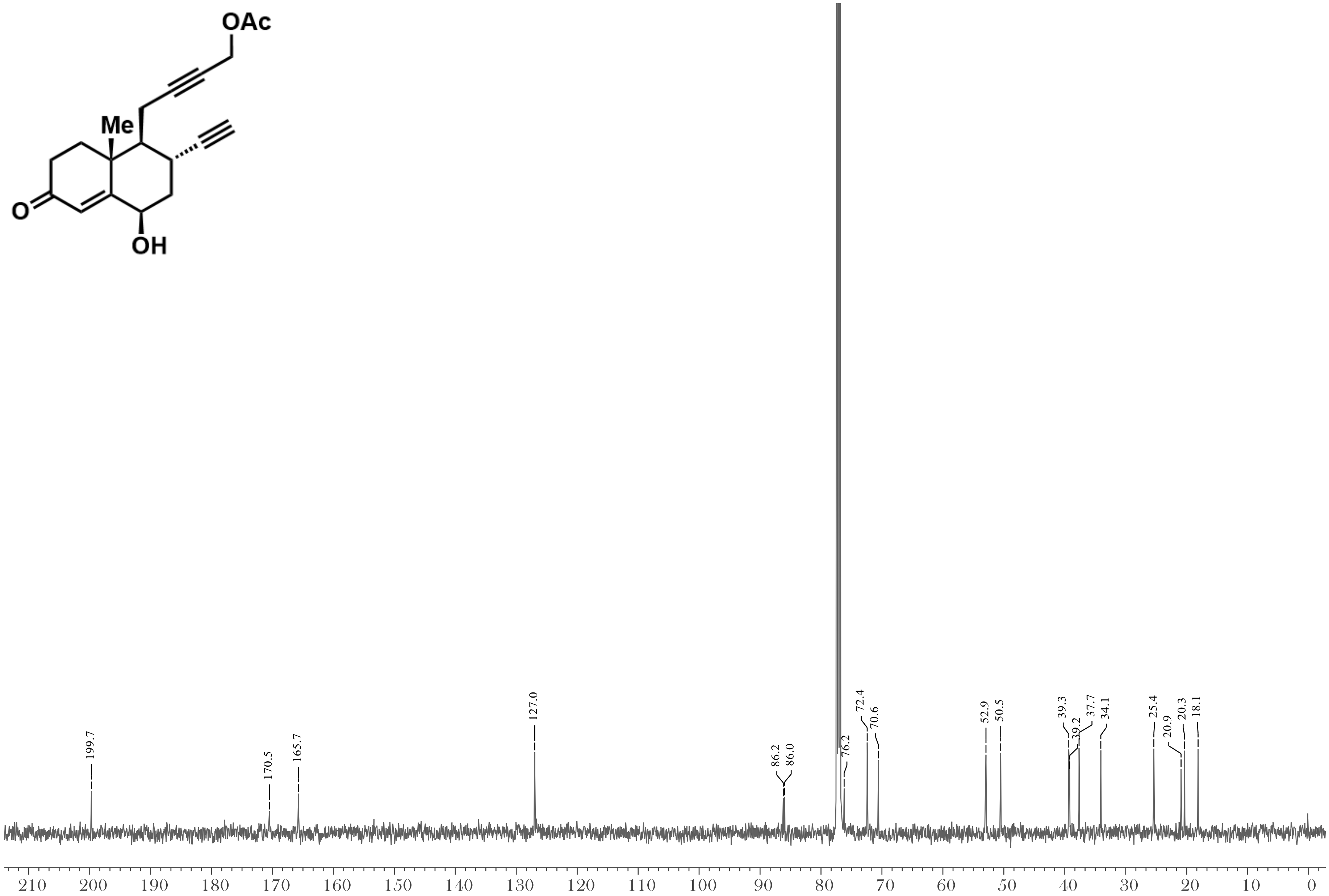

^{13}C NMR Spectrum of **14** (125 MHz, $CDCl_3$, 25 °C)

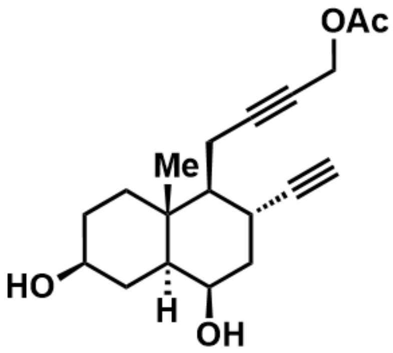

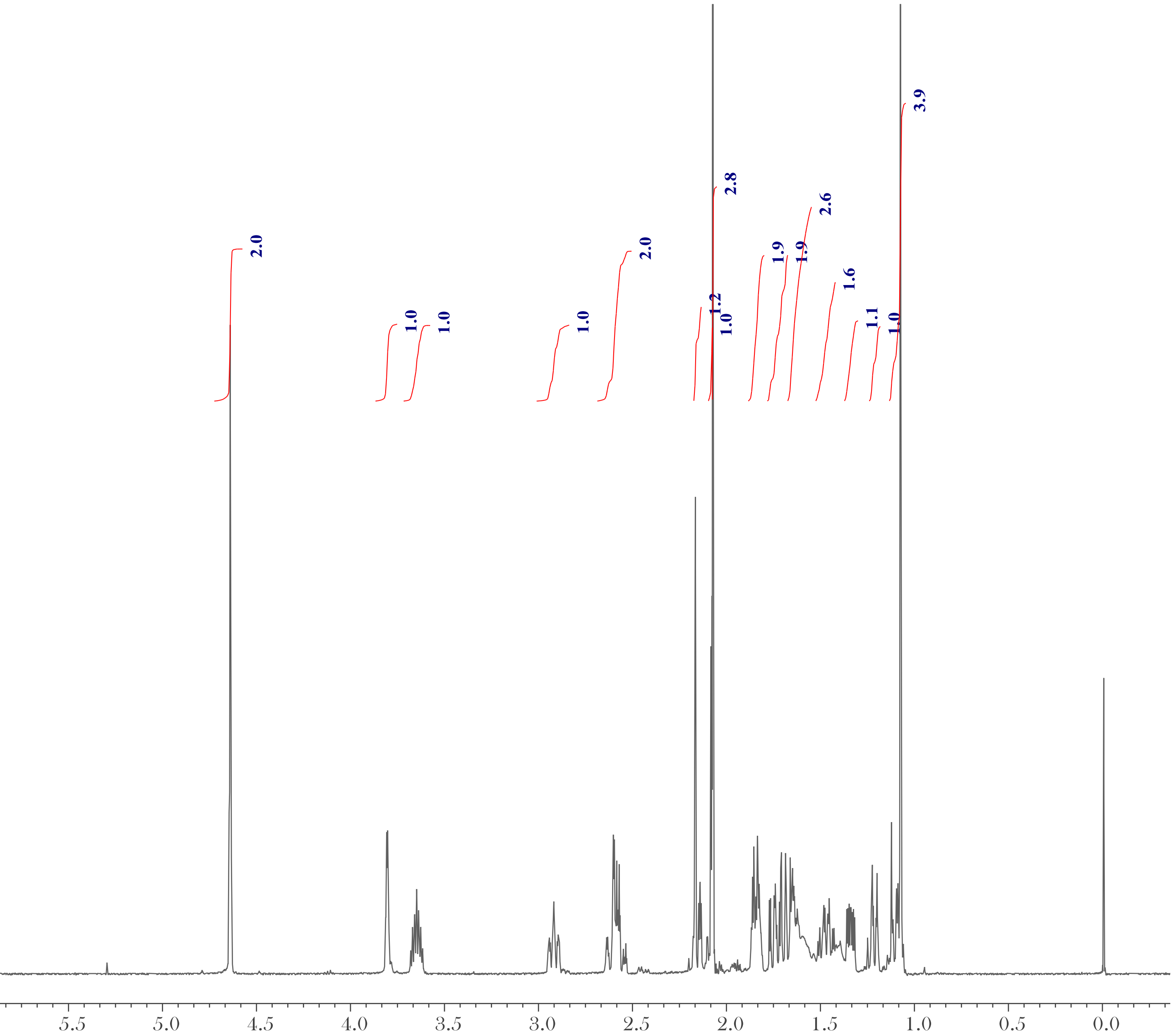

^{1}H NMR Spectrum of **15** (500 MHz, $CDCl_3$, 25 °C)

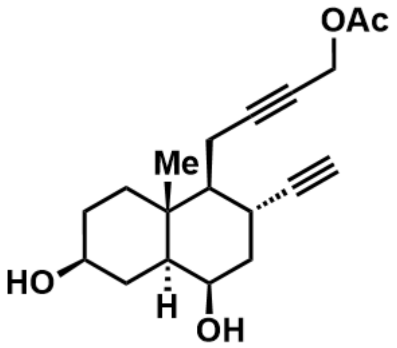

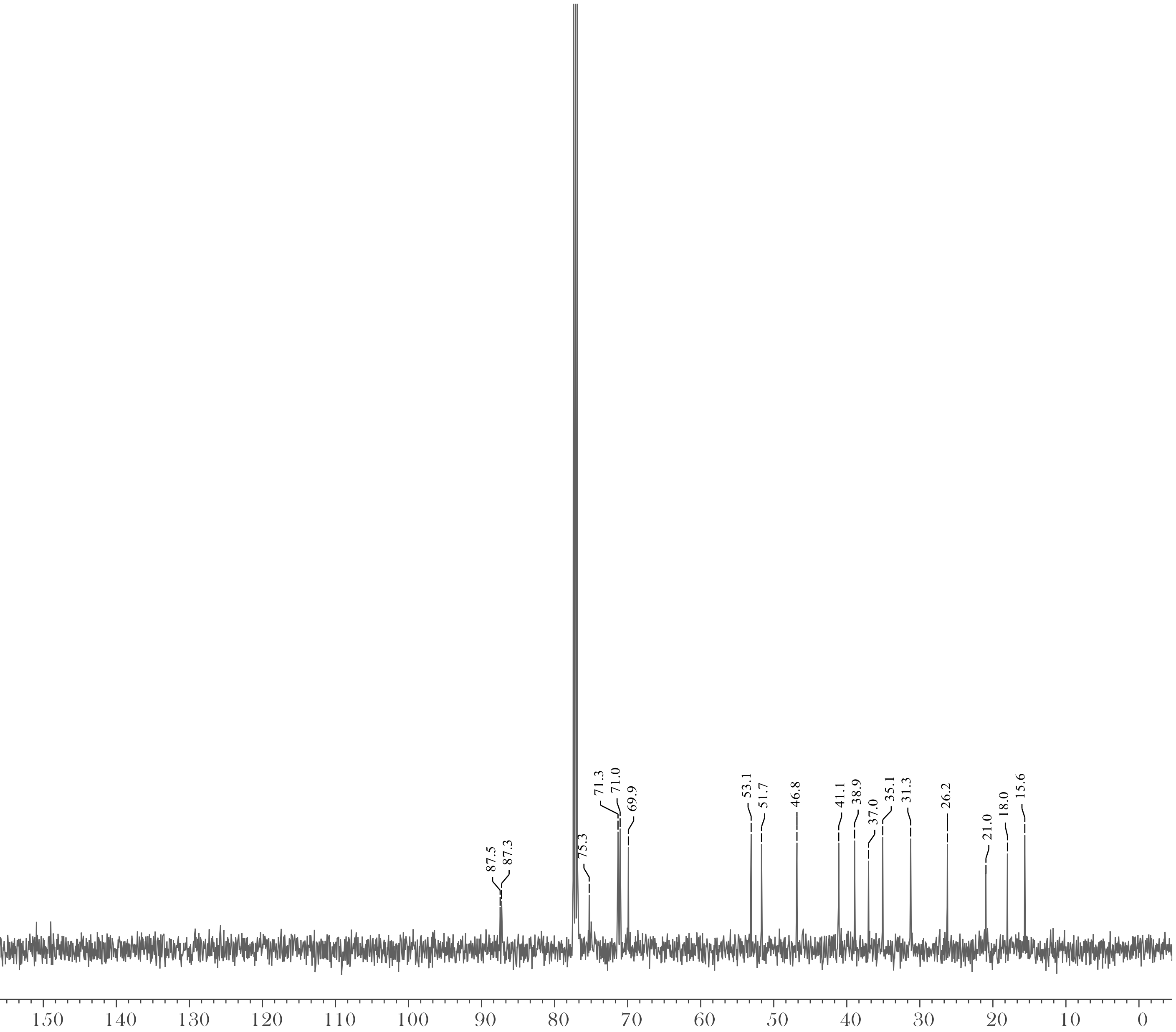

^{13}C NMR Spectrum of **15** (125 MHz, $CDCl_3$, 25 °C)

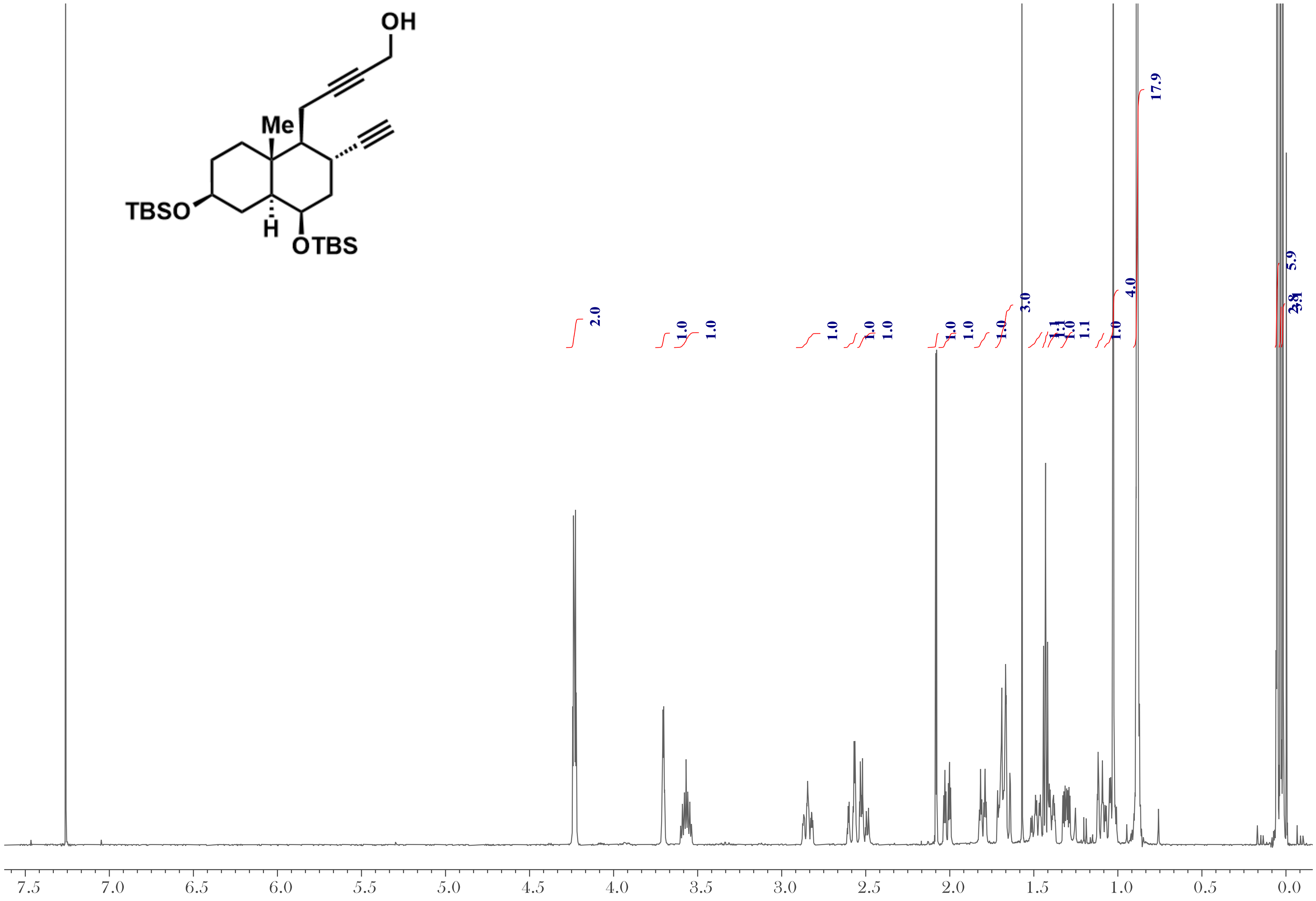

^{1}H NMR Spectrum of **16** (500 MHz, $CDCl_3$, 25 °C)

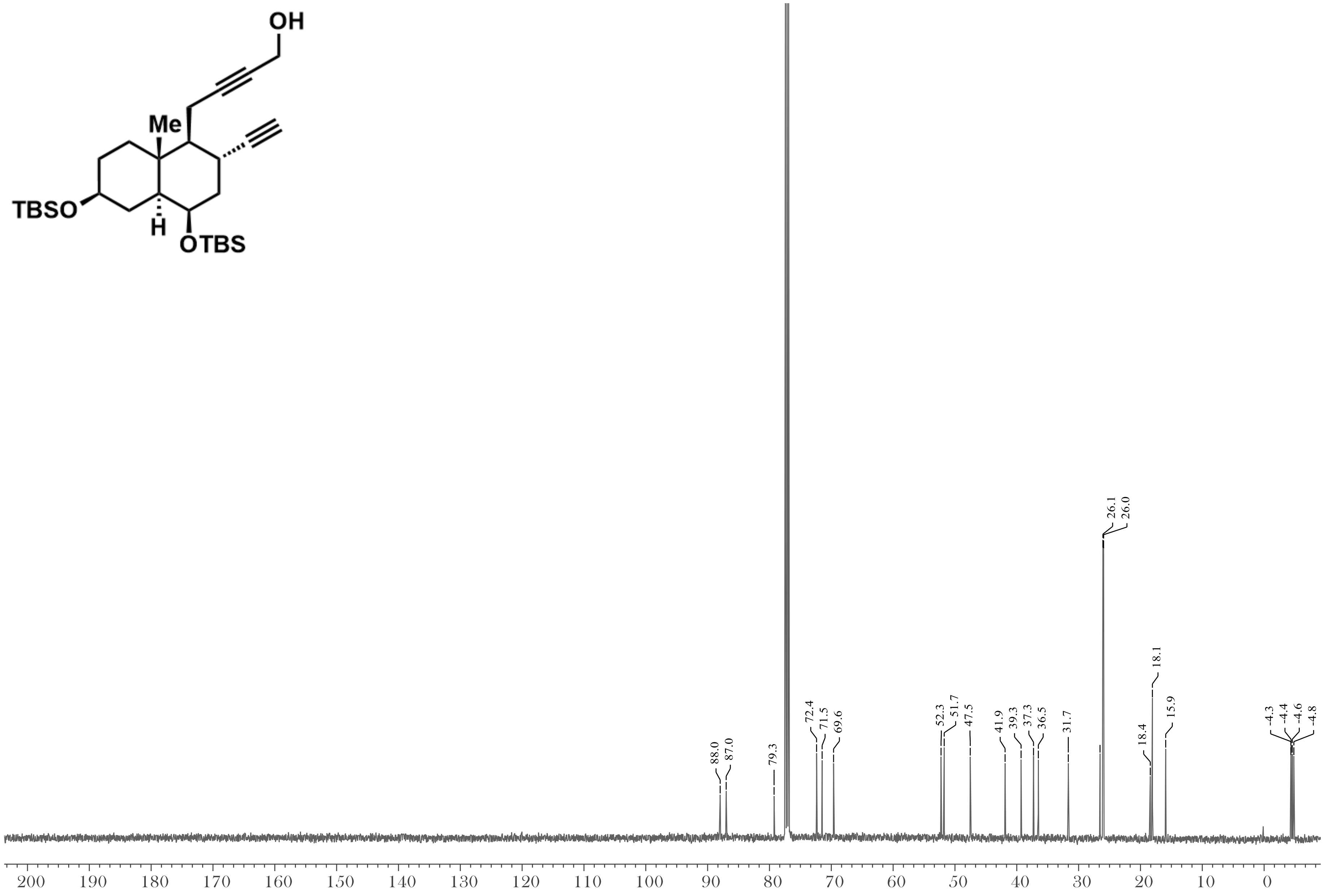

^{13}C NMR Spectrum of **16** (125 MHz, $CDCl_3$, 25 °C)

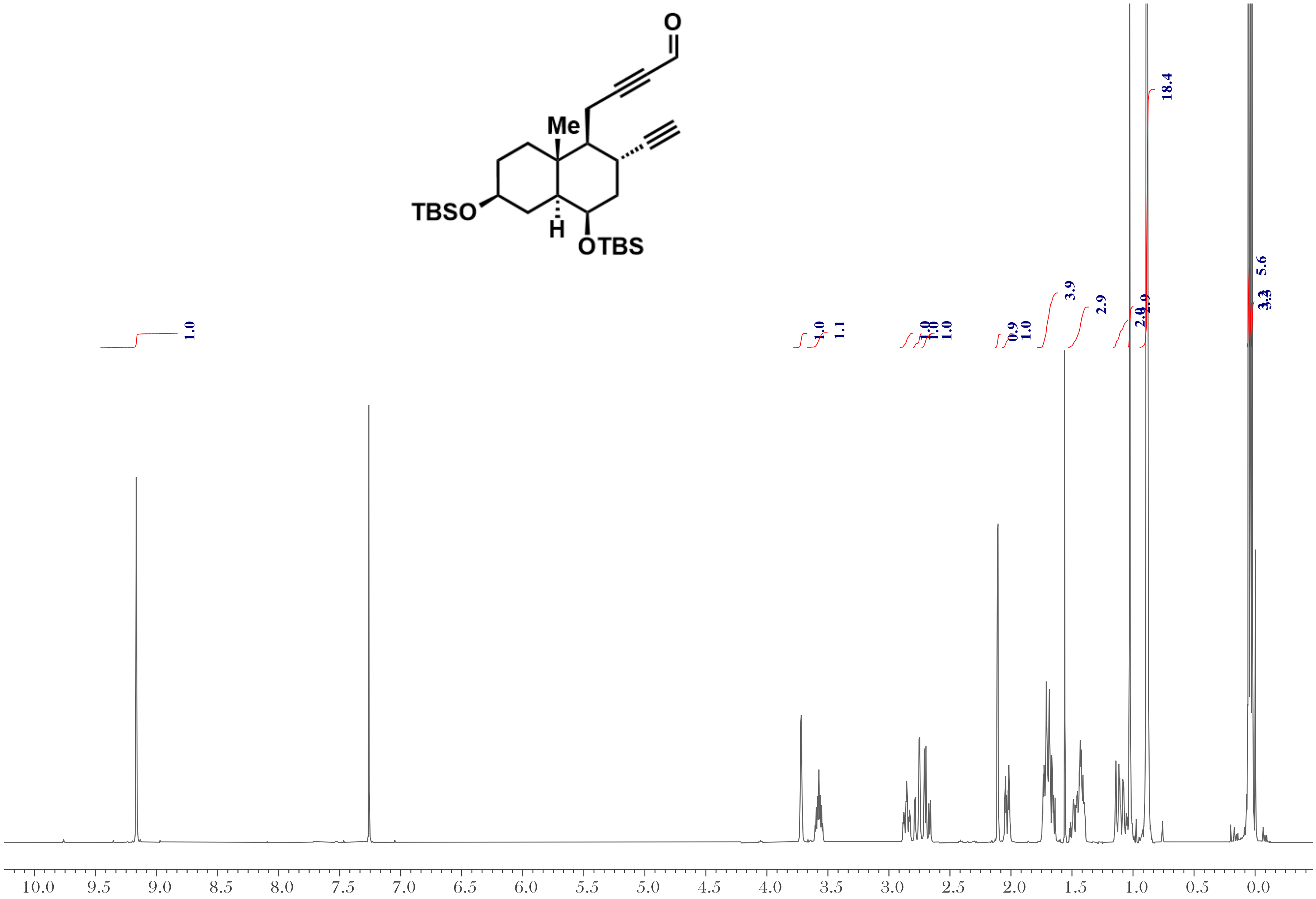

^{1}H NMR Spectrum of **17** (600 MHz, $CDCl_3$, 25 °C)

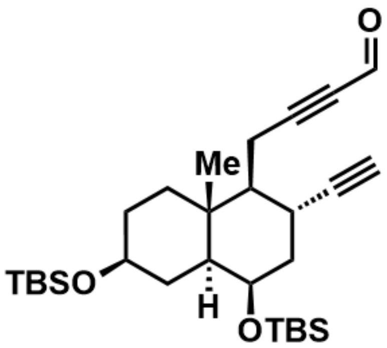

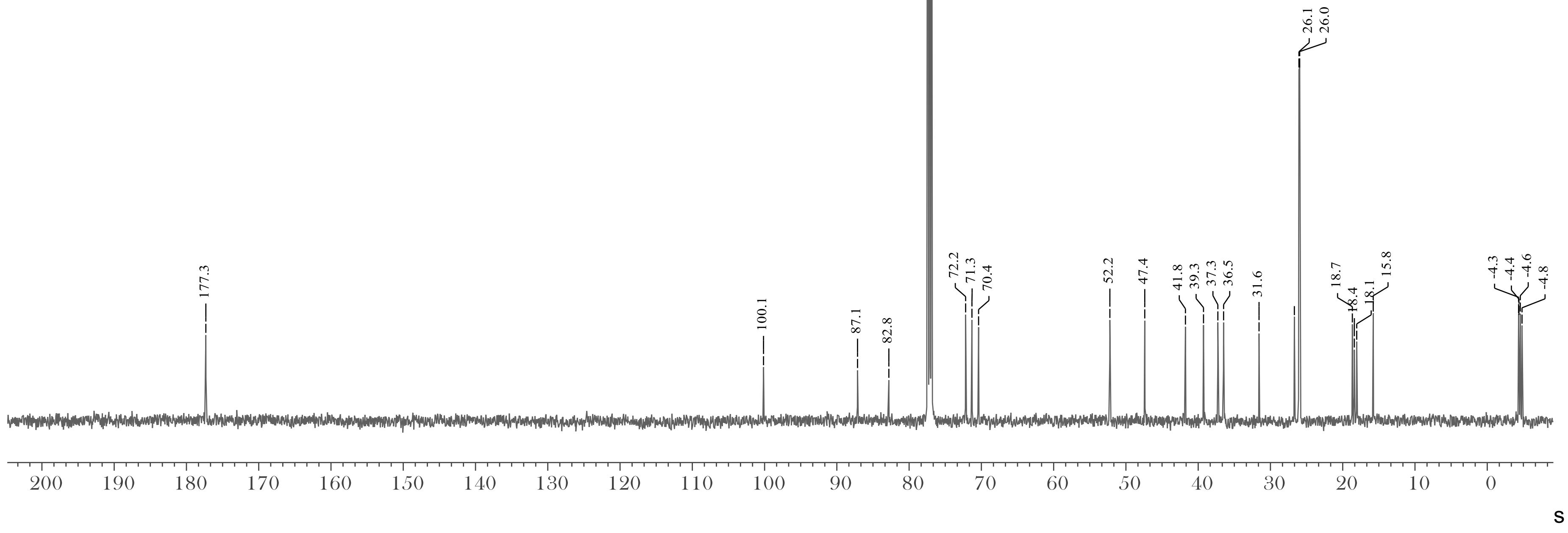

^{13}C NMR Spectrum of **17** (150 MHz, $CDCl_3$, 25 °C)

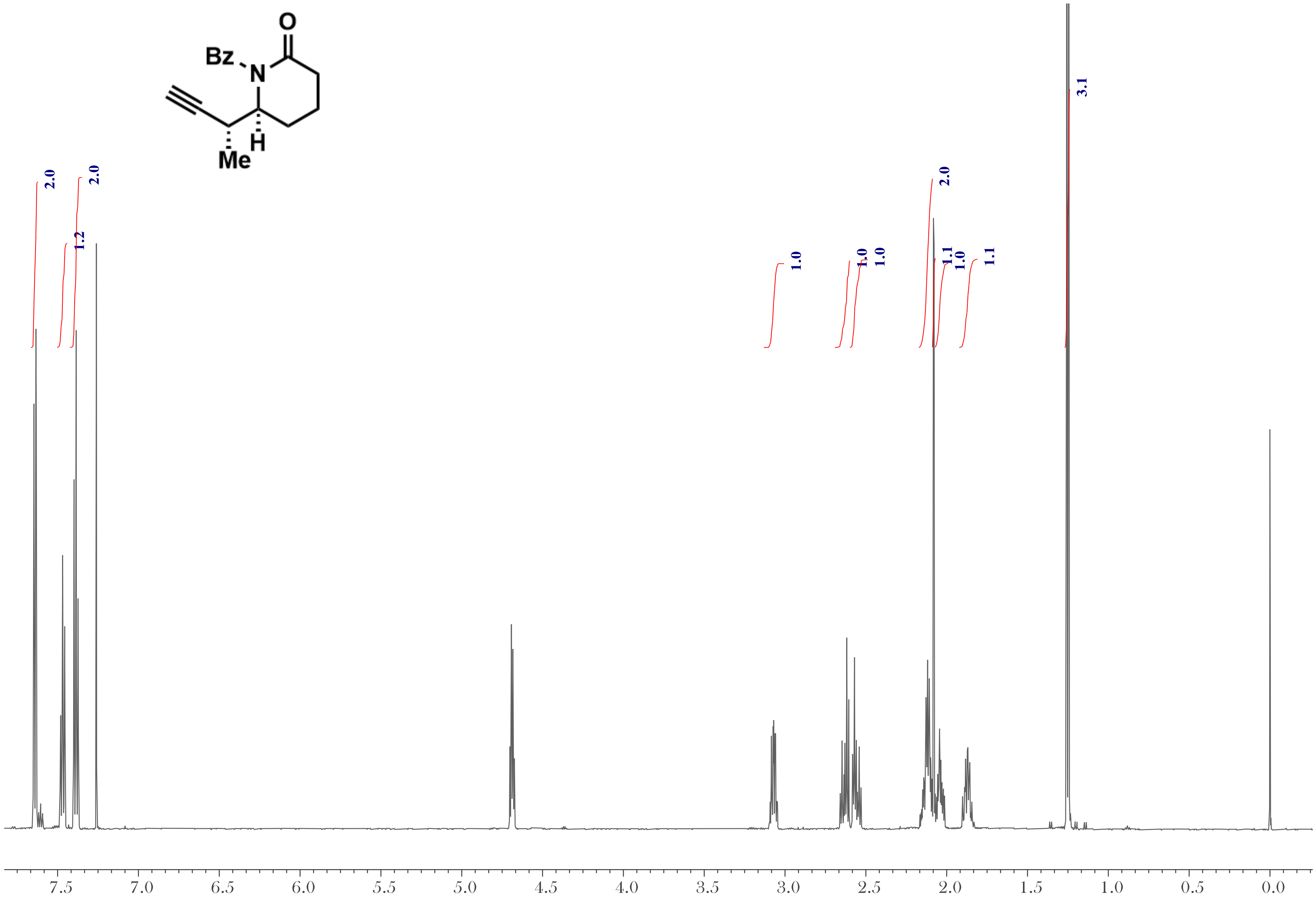

^{1}H NMR Spectrum of **19** (600 MHz, $CDCl_3$, 25 °C)

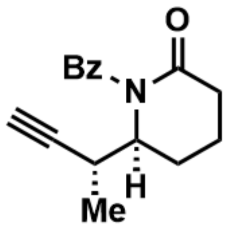

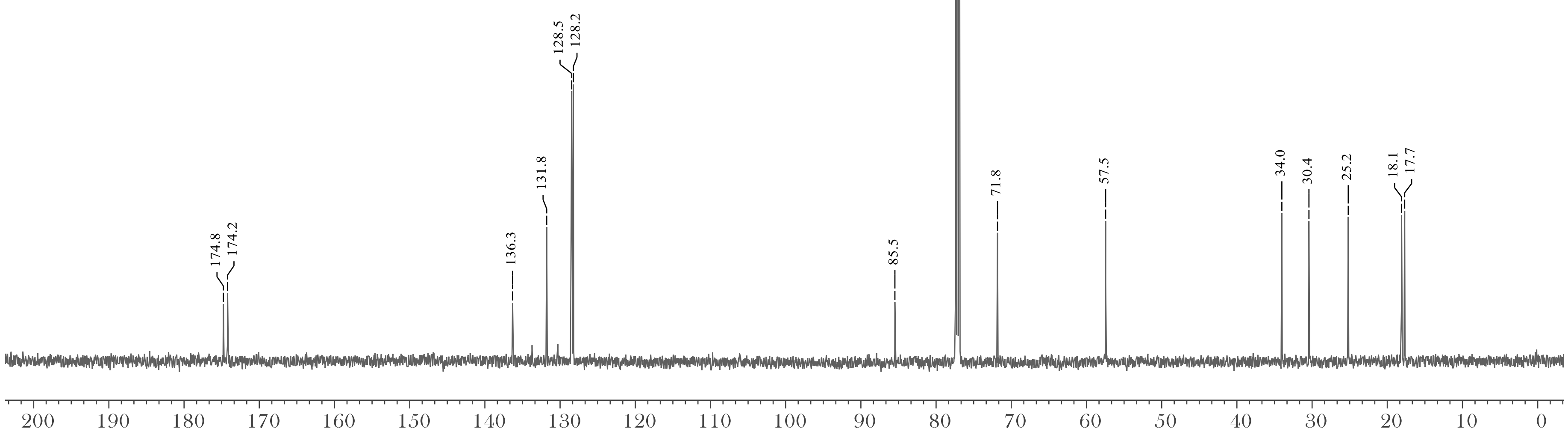

^{13}C NMR Spectrum of **19** (150 MHz, $CDCl_3$, 25 °C)

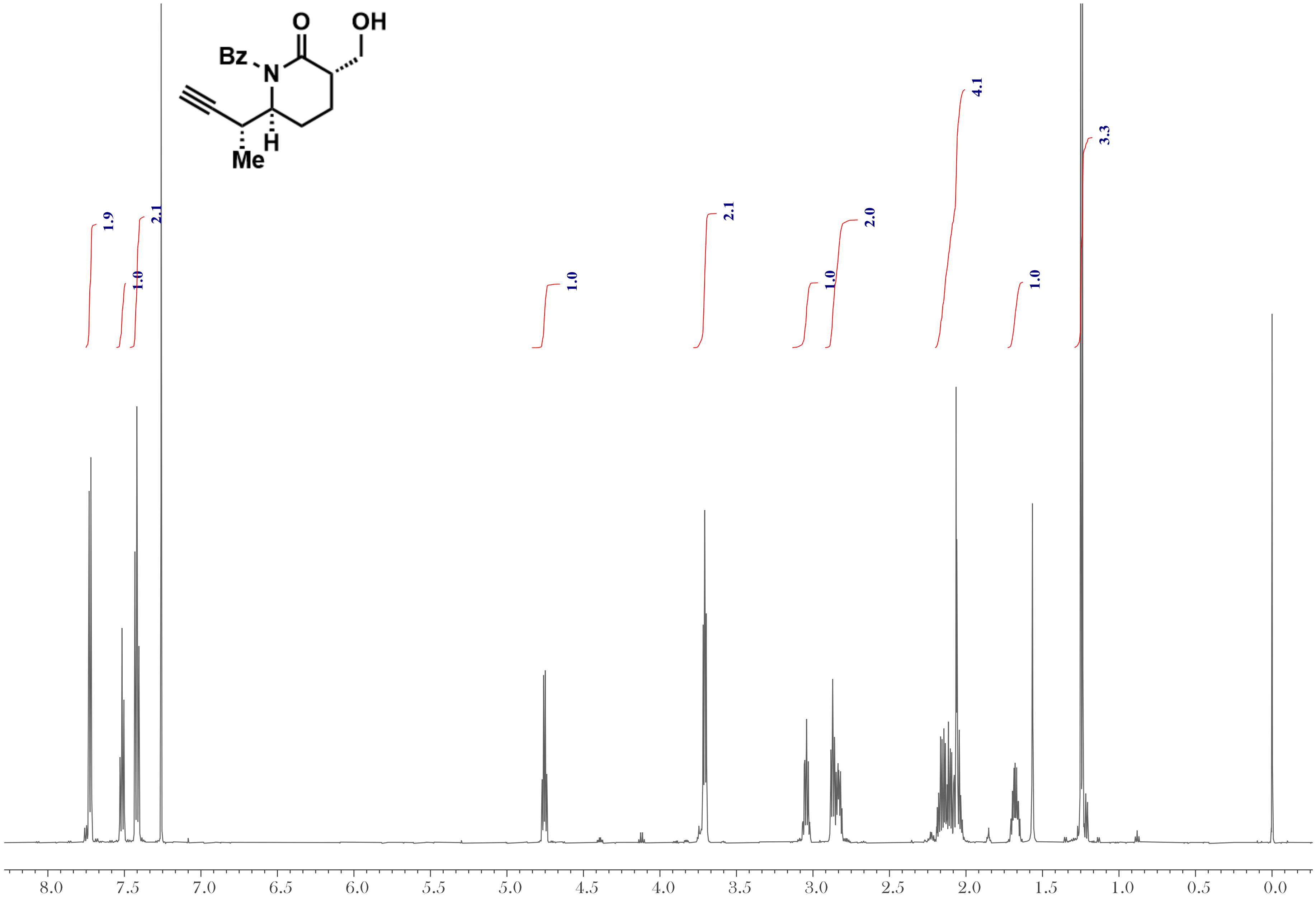

^{1}H NMR Spectrum of **21** (600 MHz, $CDCl_3$, 25 °C)

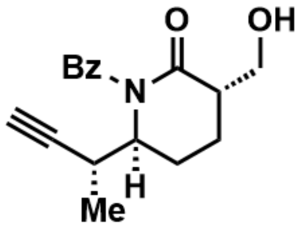

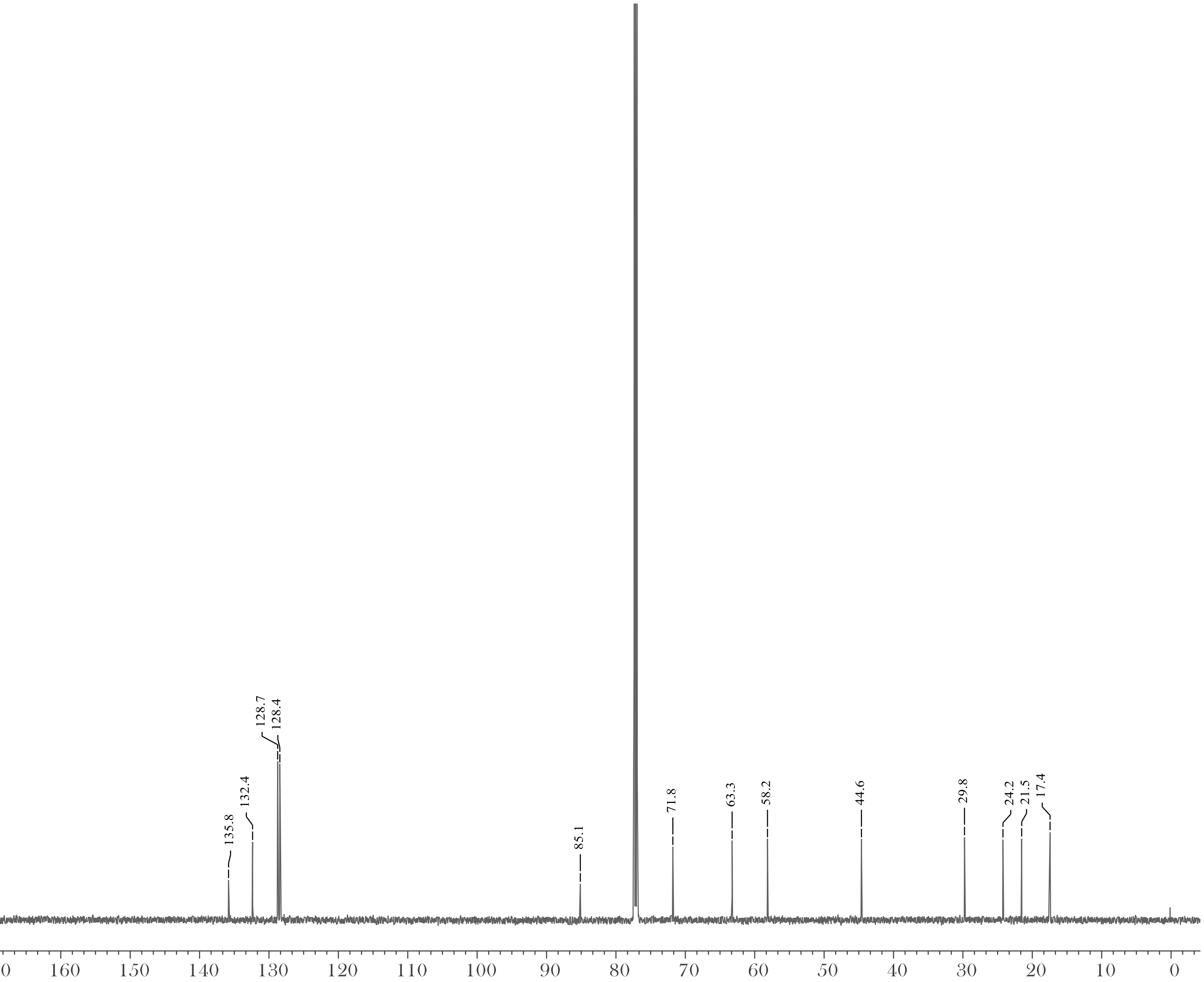

^{13}C NMR Spectrum of **21** (150 MHz, $CDCl_3$, 25 °C)

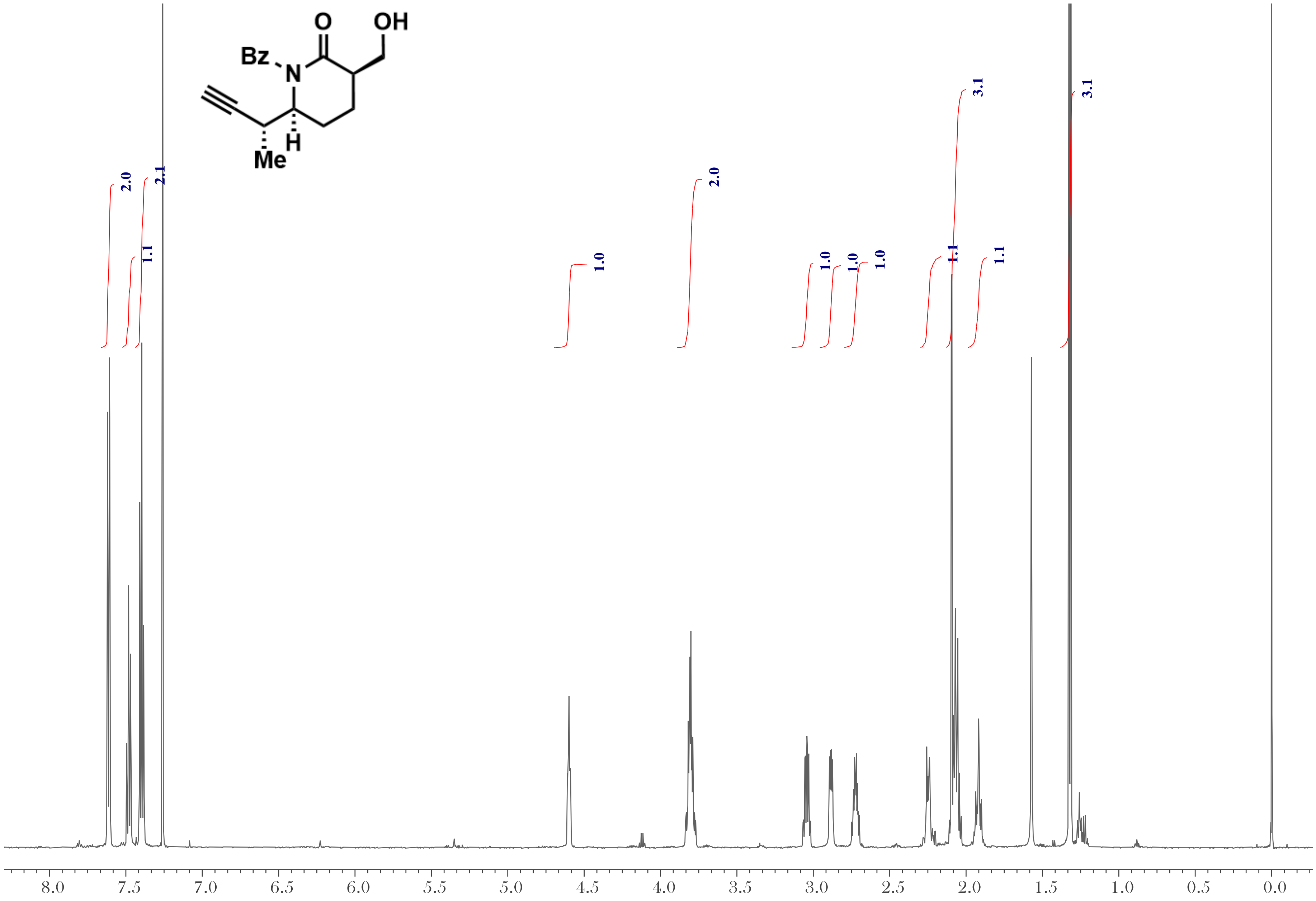

^{1}H NMR Spectrum of **22** (600 MHz, $CDCl_3$, 25 °C)

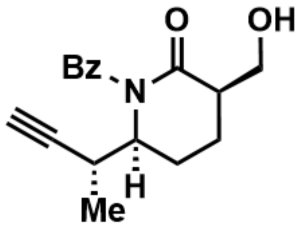

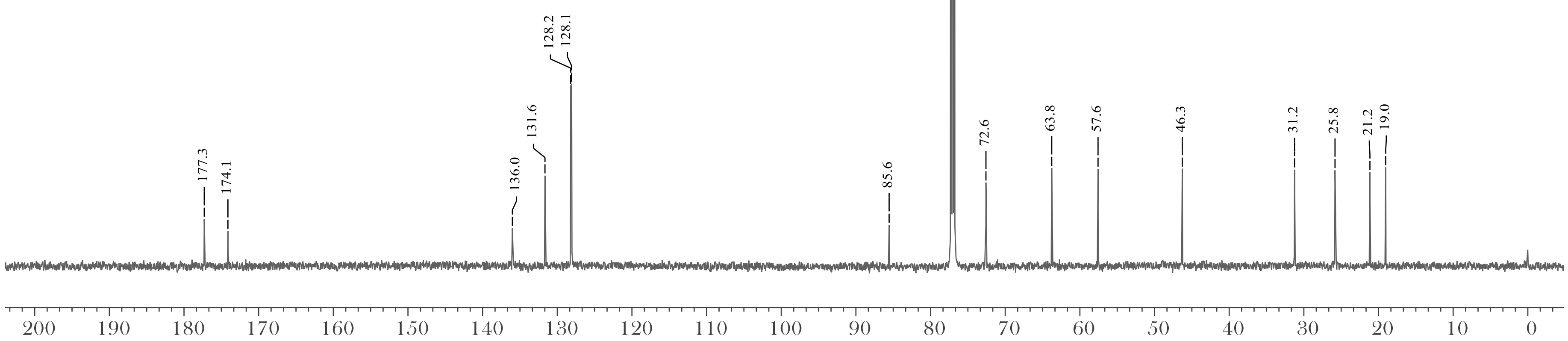

^{13}C NMR Spectrum of **22** (150 MHz, $CDCl_3$, 25 °C)

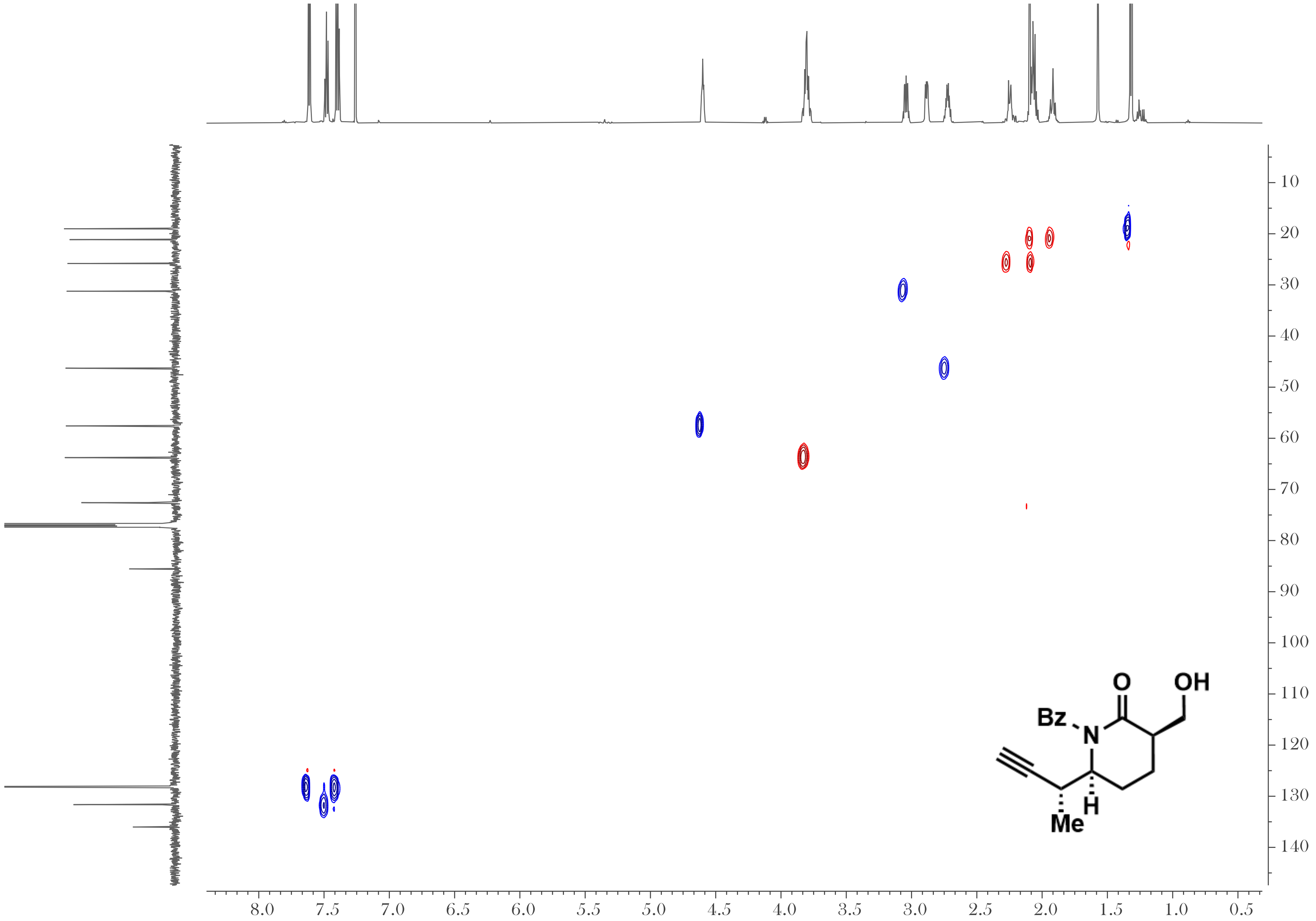

^{1}H–^{13}C HSQC Spectrum of **22** (150 MHz, $CDCl_3$, 25 °C)

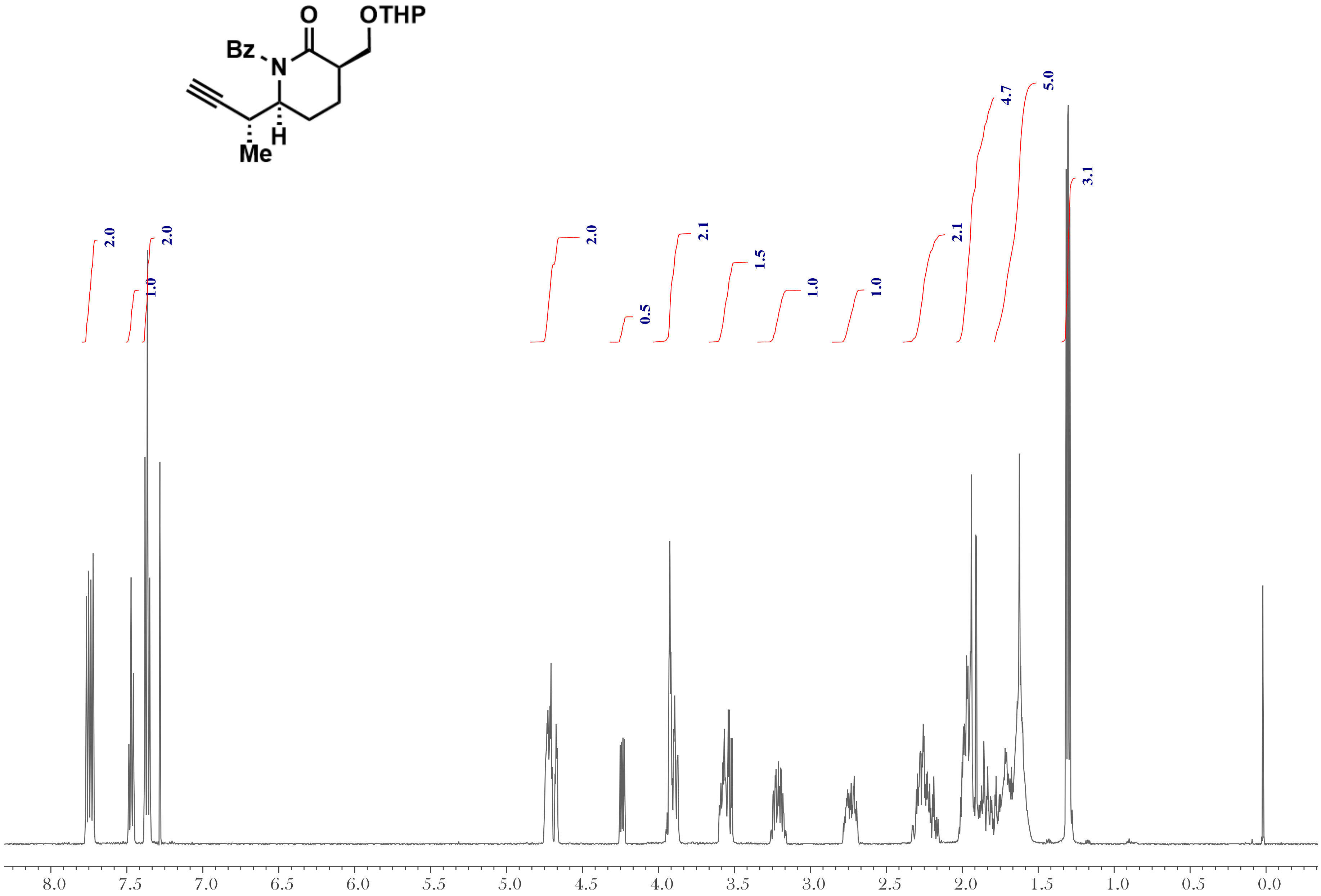

^{1}H NMR Spectrum of **23** (500 MHz, $CDCl_3$, 25 °C)

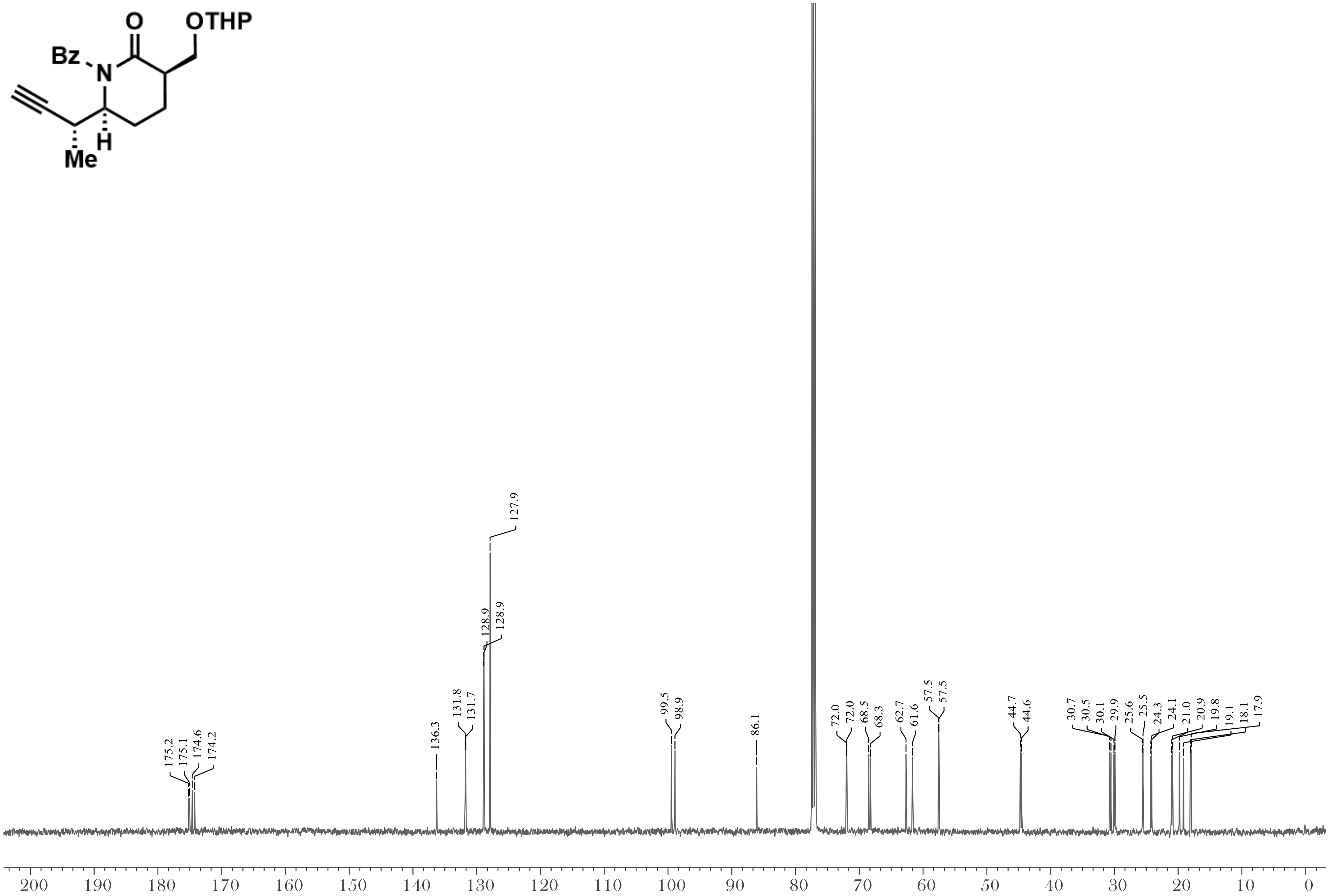

^{13}C NMR Spectrum of **23** (125 MHz, $CDCl_3$, 25 °C)

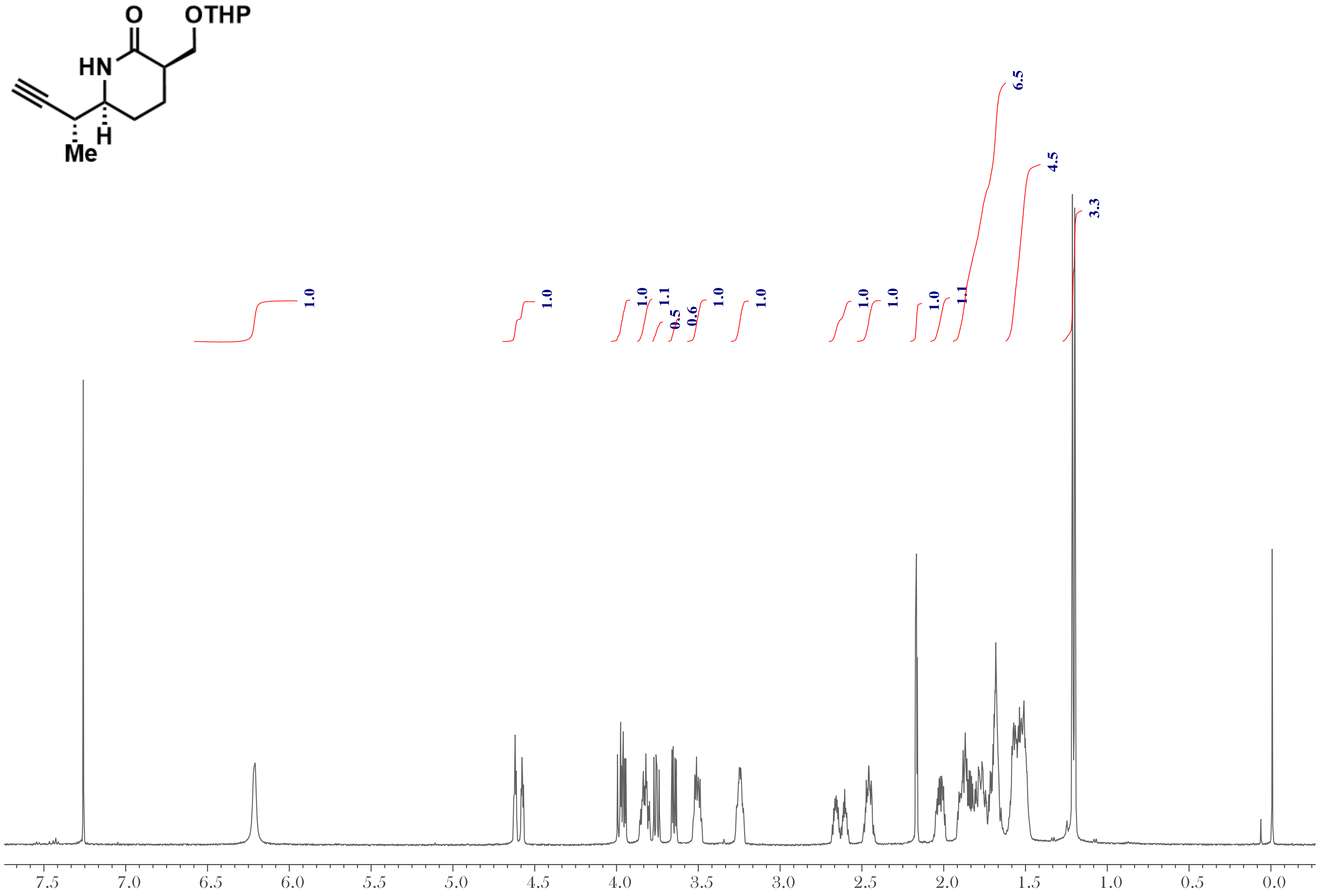

^{1}H NMR Spectrum of **24** (500 MHz, $CDCl_3$, 25 °C)

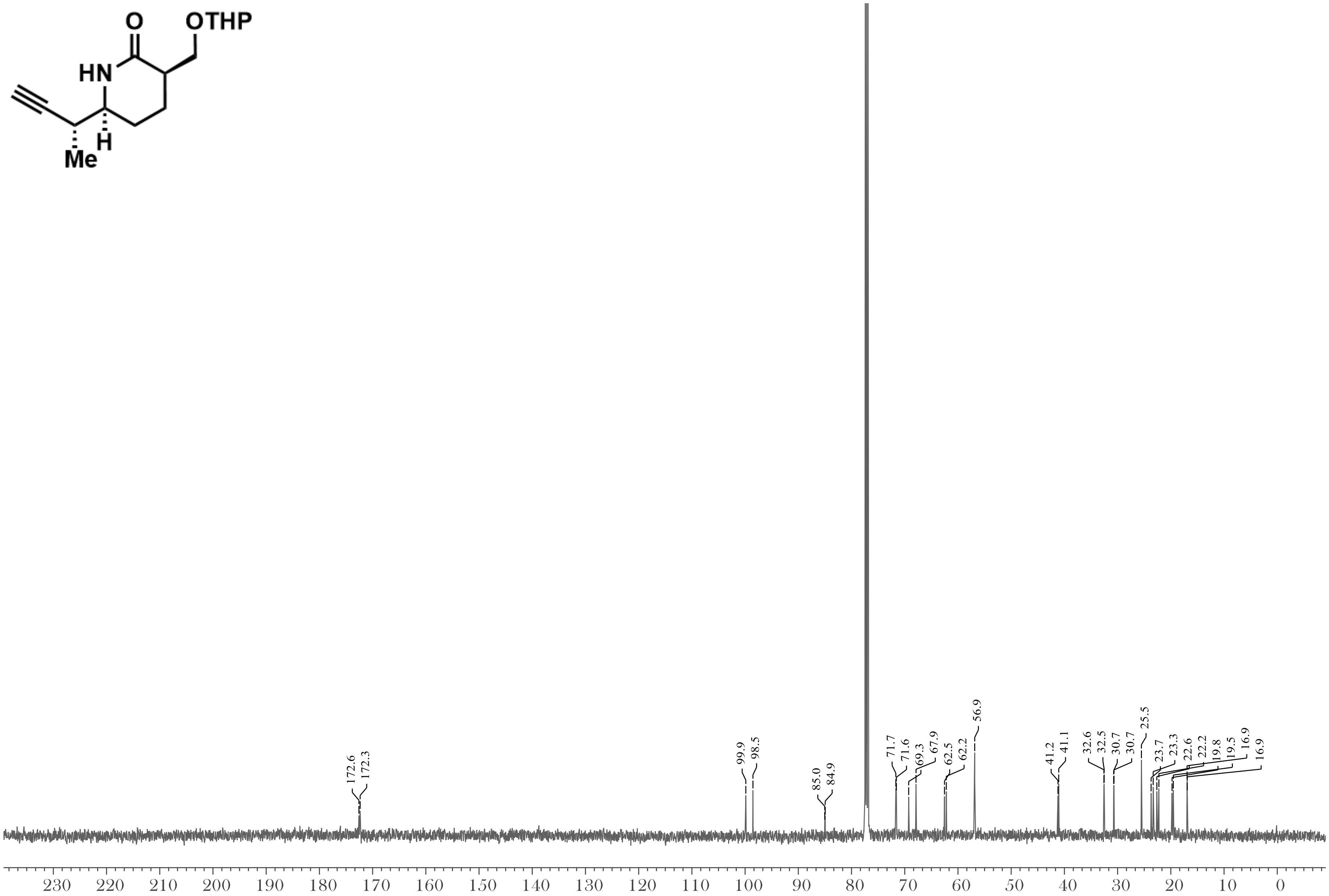

^{13}C NMR Spectrum of **24** (125 MHz, $CDCl_3$, 25 °C)

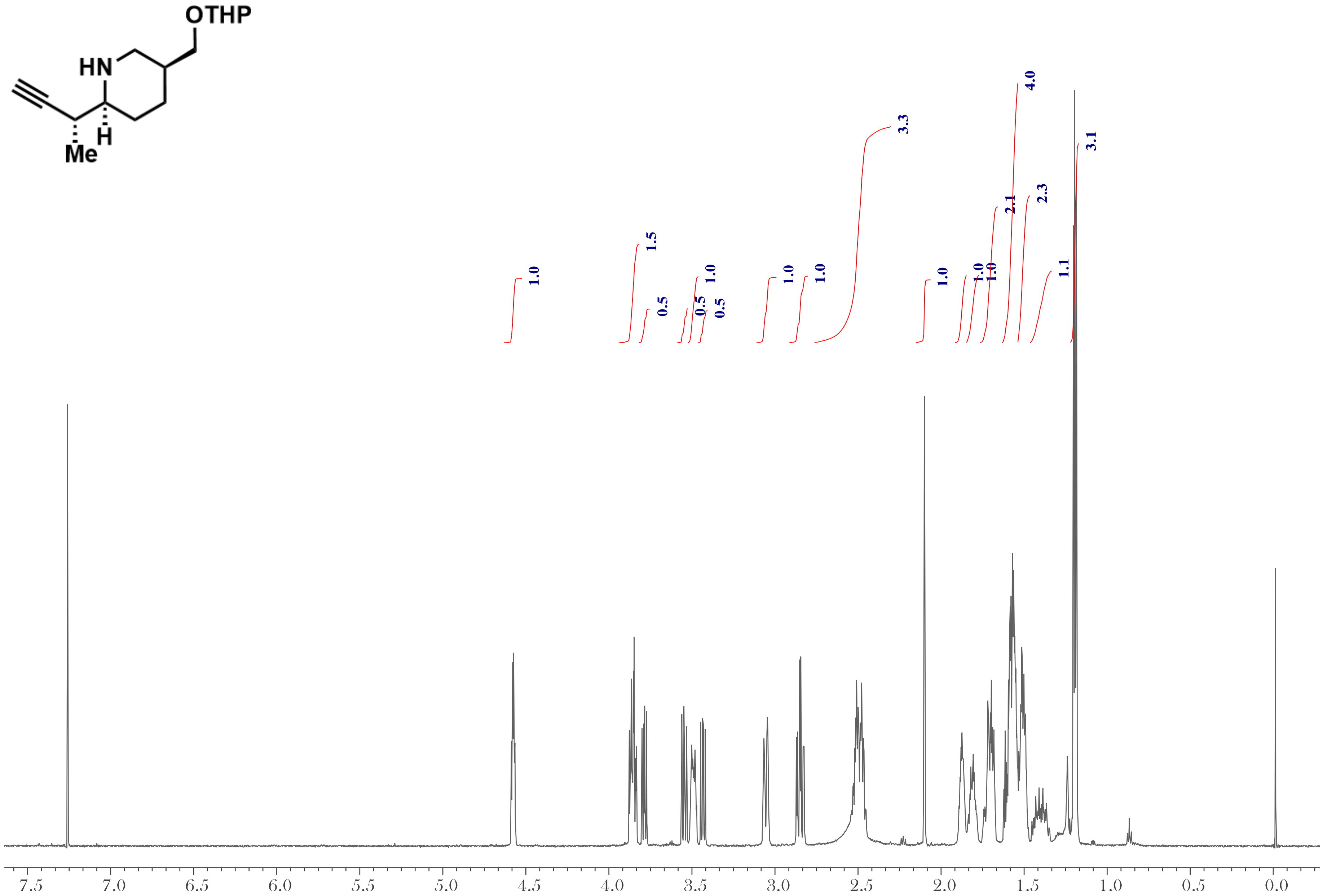

^{1}H NMR Spectrum of **25** (600 MHz, $CDCl_3$, 25 °C)

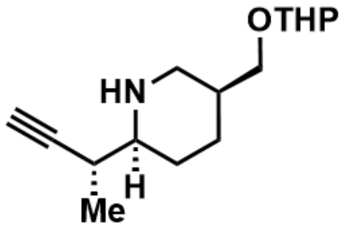

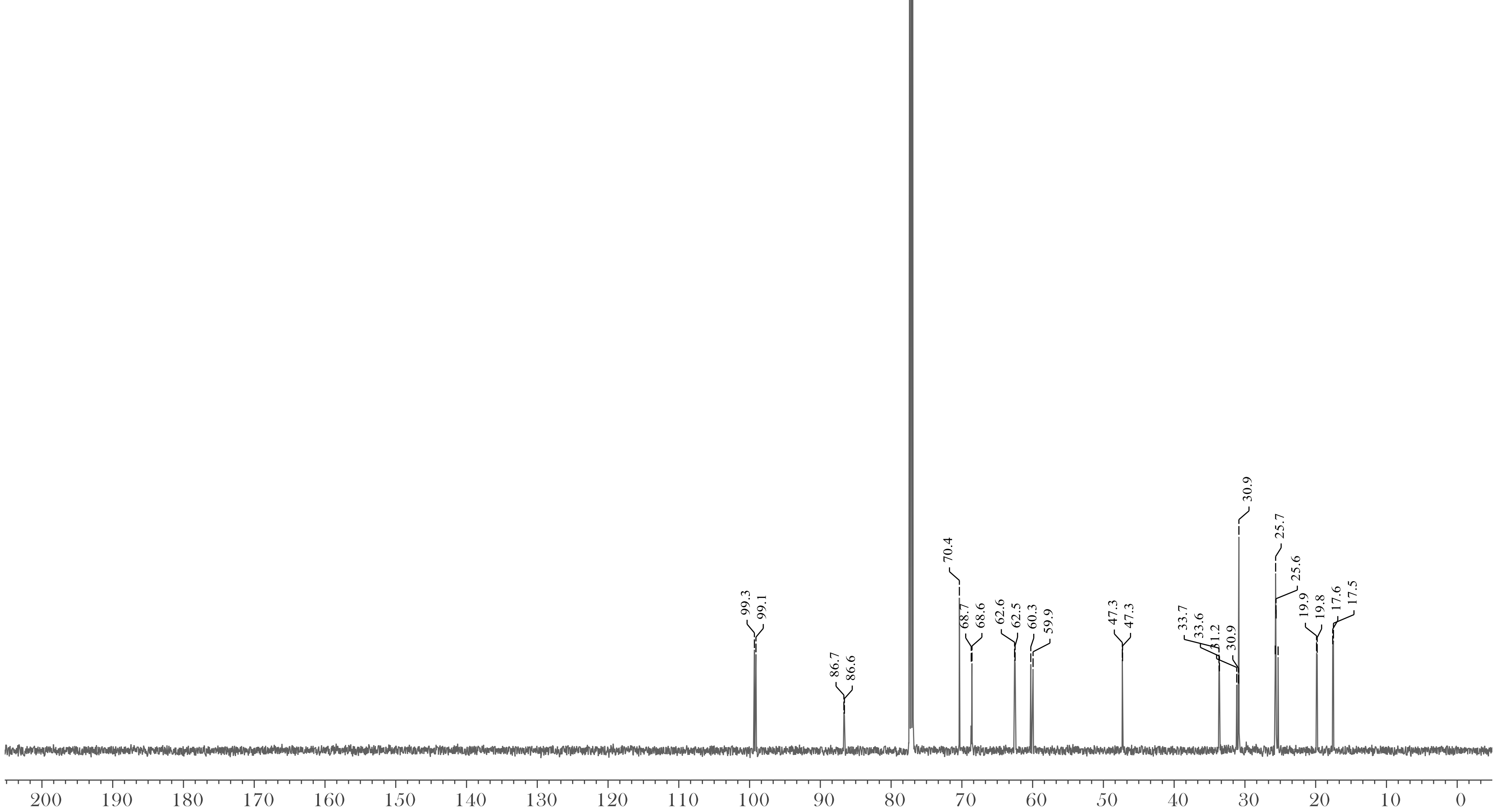

^{13}C NMR Spectrum of **25** (150 MHz, $CDCl_3$, 25 °C)

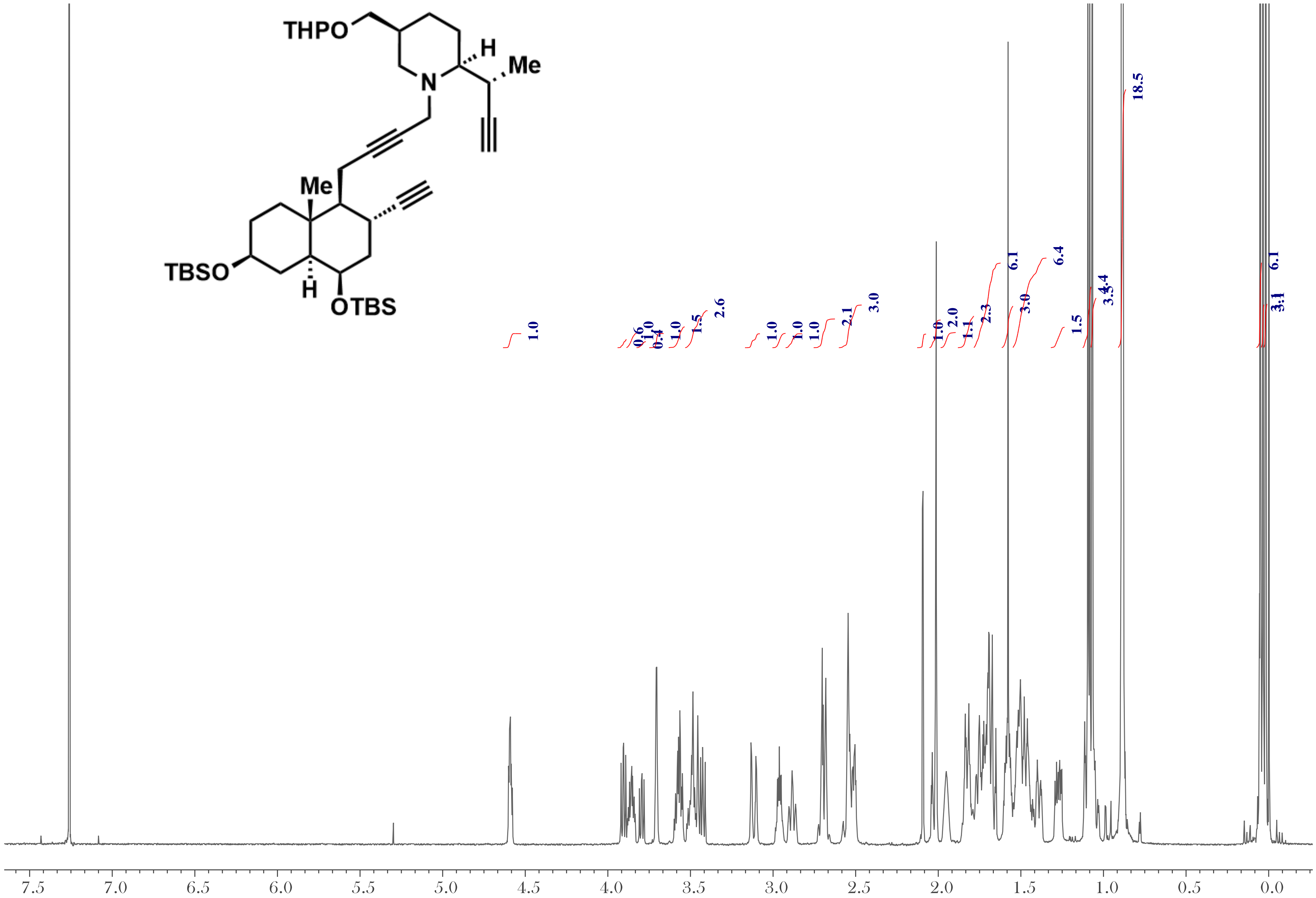

^{1}H NMR Spectrum of **26** (600 MHz, $CDCl_3$, 25 °C)

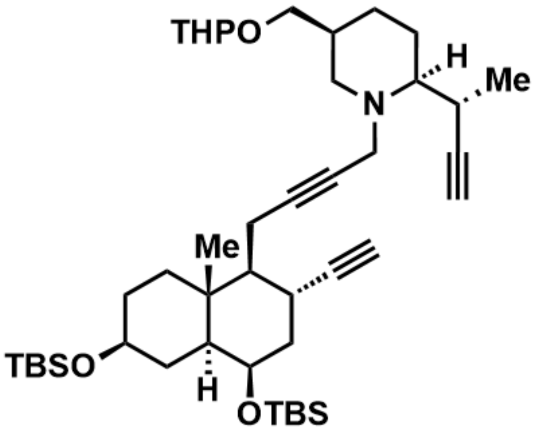

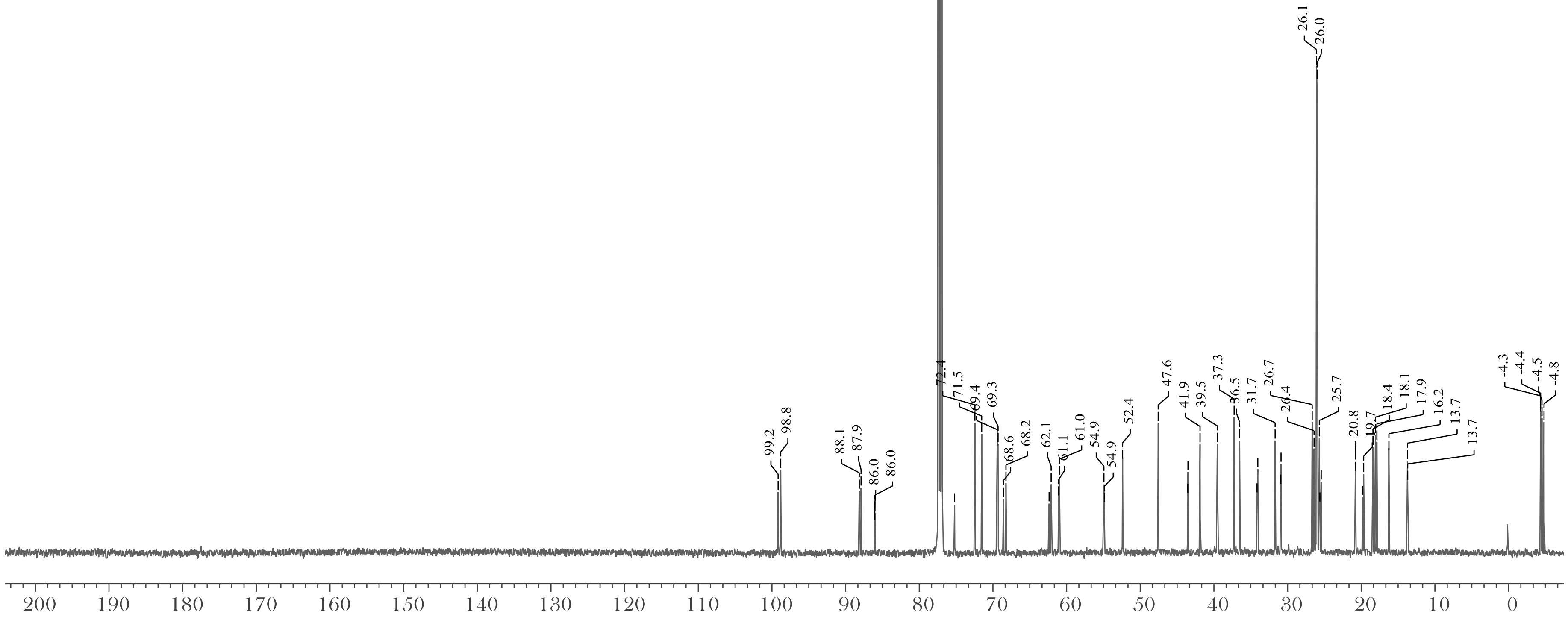

^{13}C NMR Spectrum of **26** (150 MHz, $CDCl_3$, 25 °C)

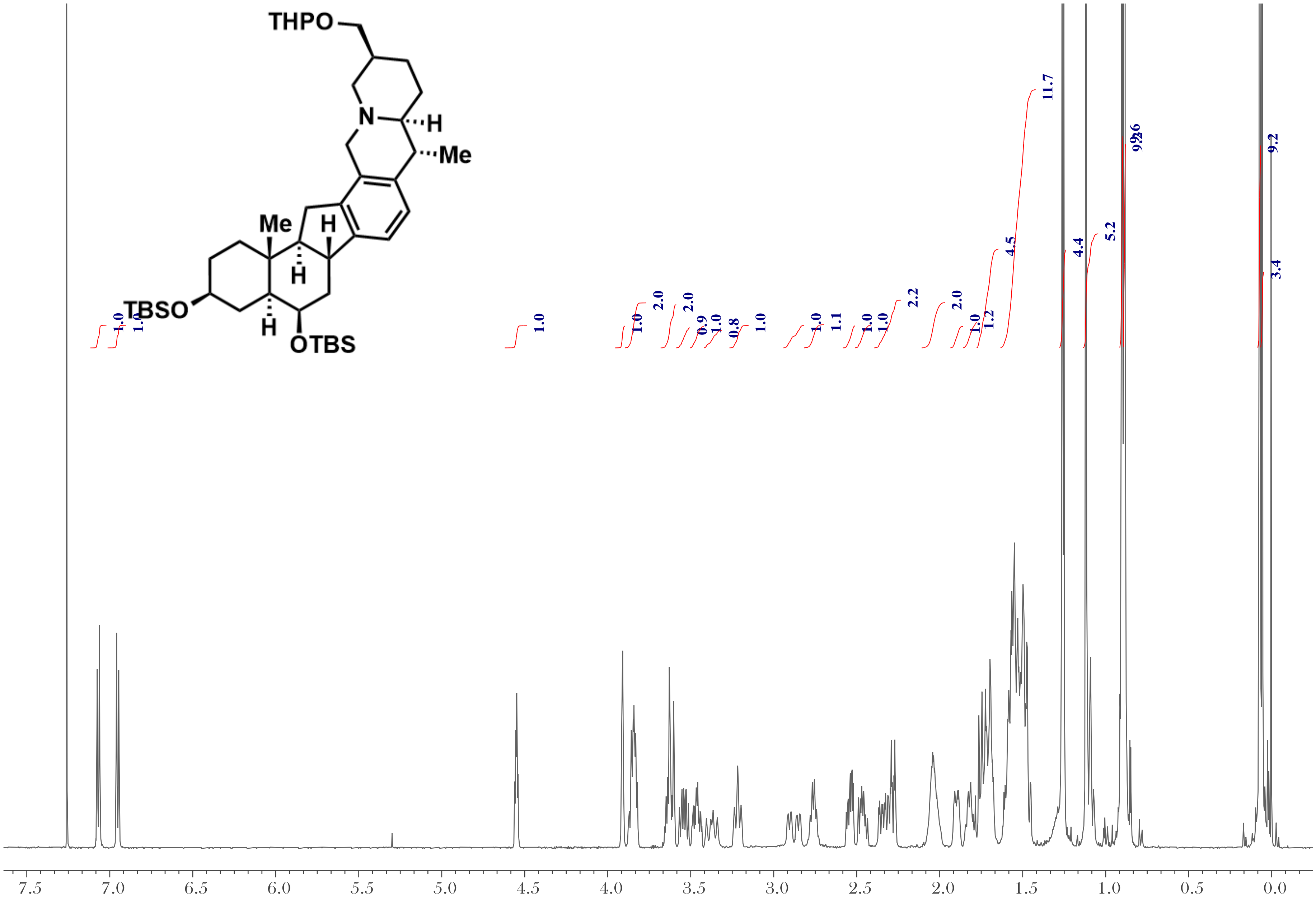

^{1}H NMR Spectrum of **27** (600 MHz, $CDCl_3$, 25 °C)

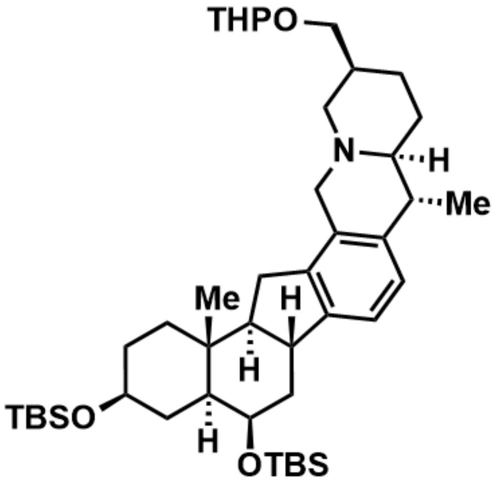

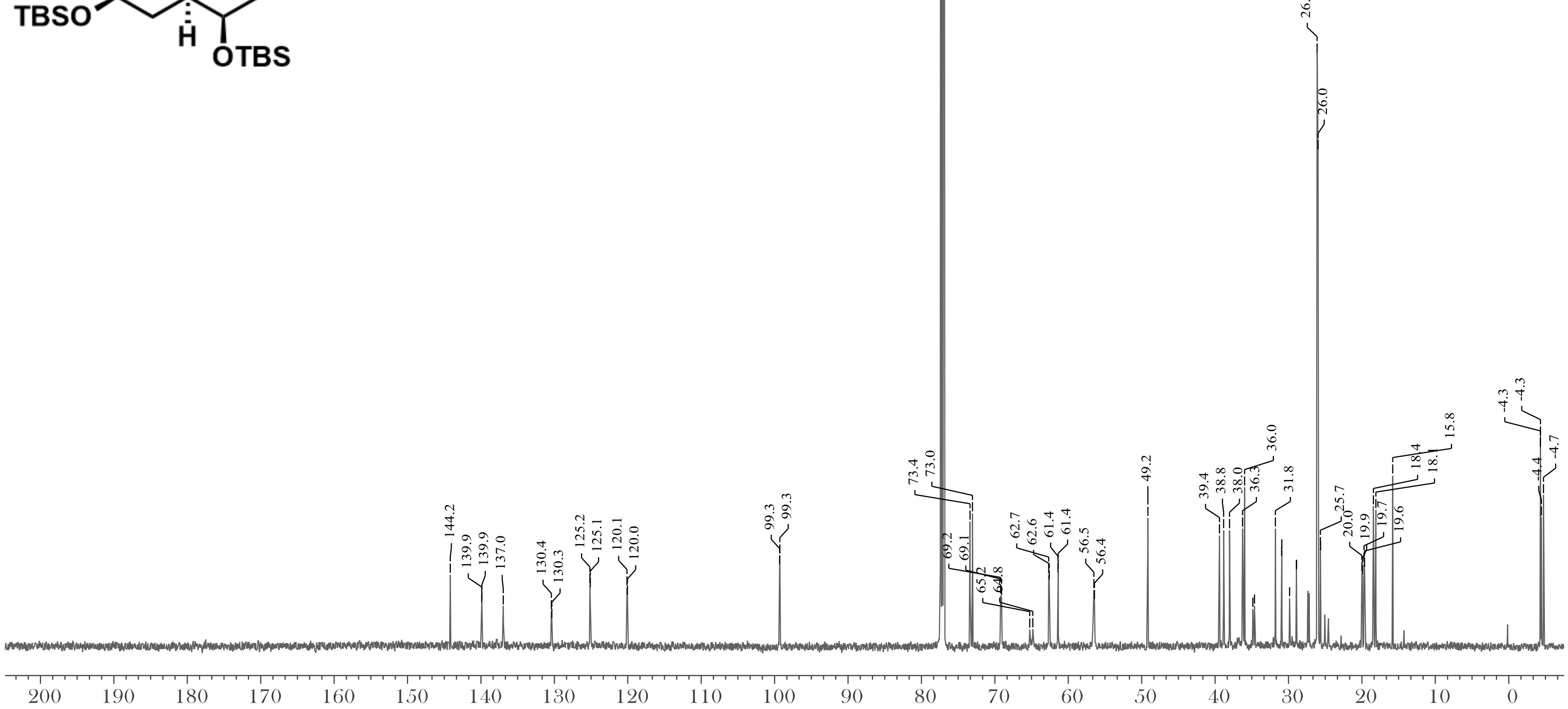

^{13}C NMR Spectrum of **27** (150 MHz, CDCl$_3$, 25 °C)

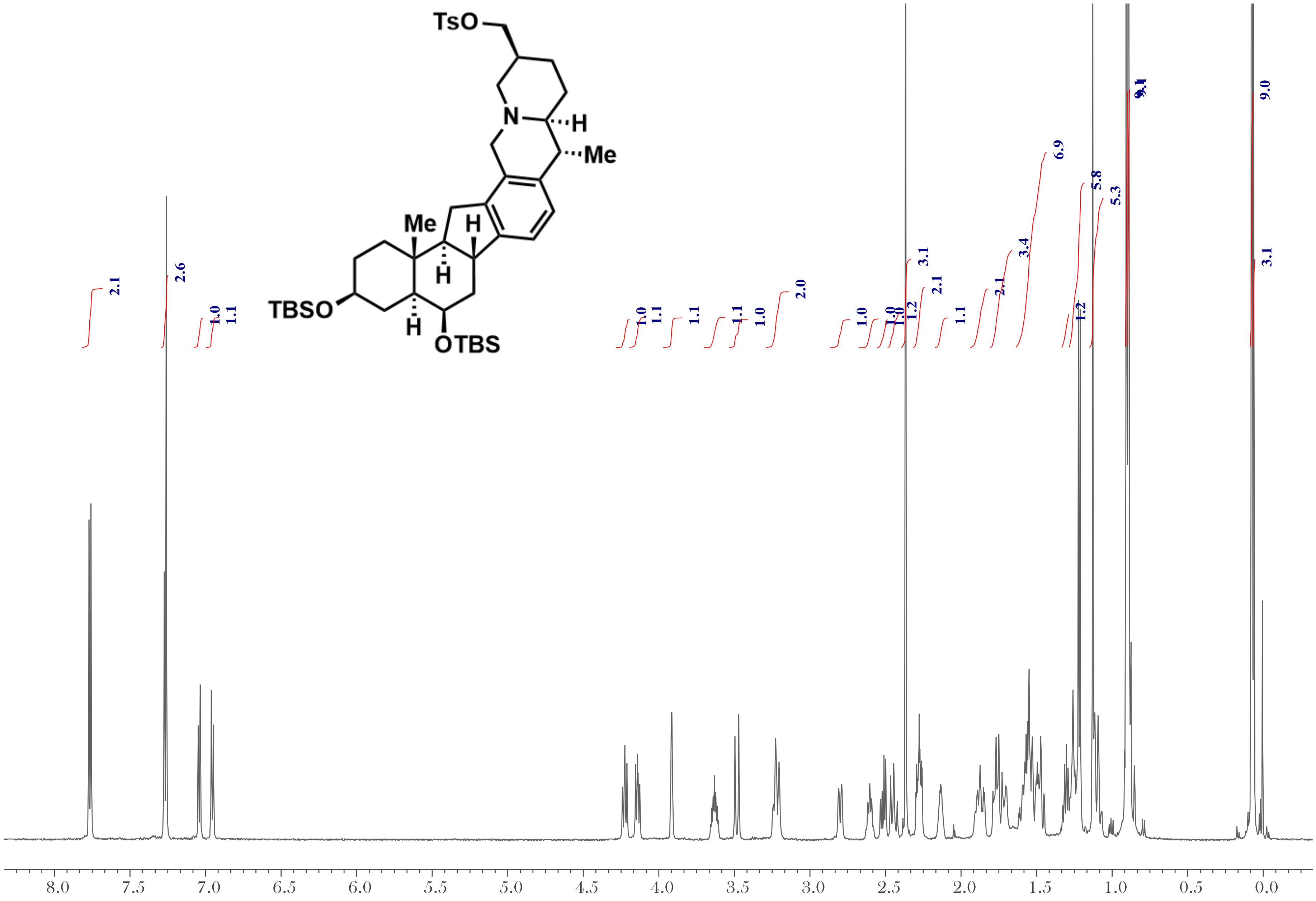

^{1}H NMR Spectrum of **28** (600 MHz, $CDCl_3$, 25 °C)

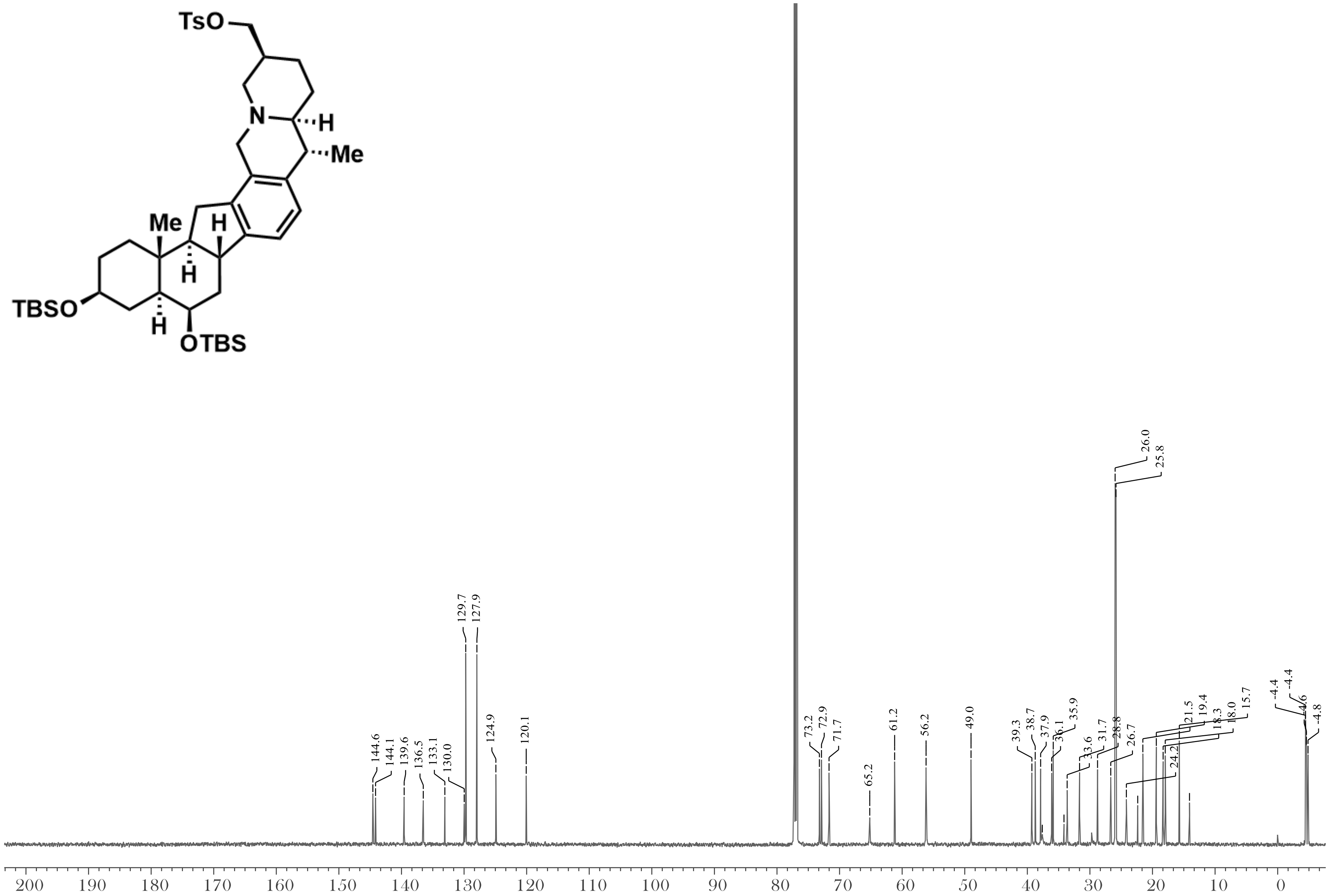

^{13}C NMR Spectrum of **28** (150 MHz, $CDCl_3$, 25 °C)

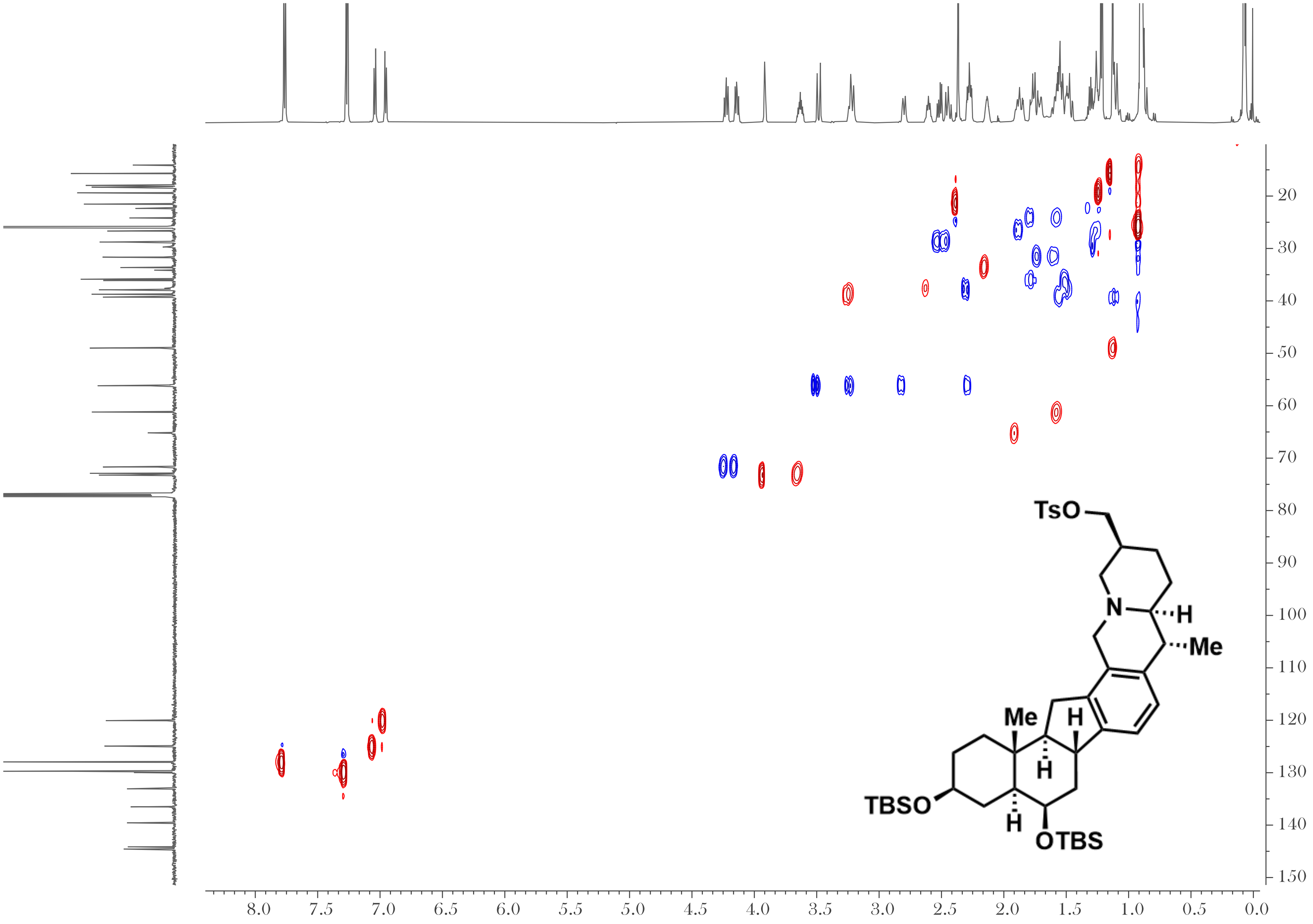

^{1}H–^{13}C HSQC Spectrum of **28** (150 MHz, $CDCl_3$, 25 °C)

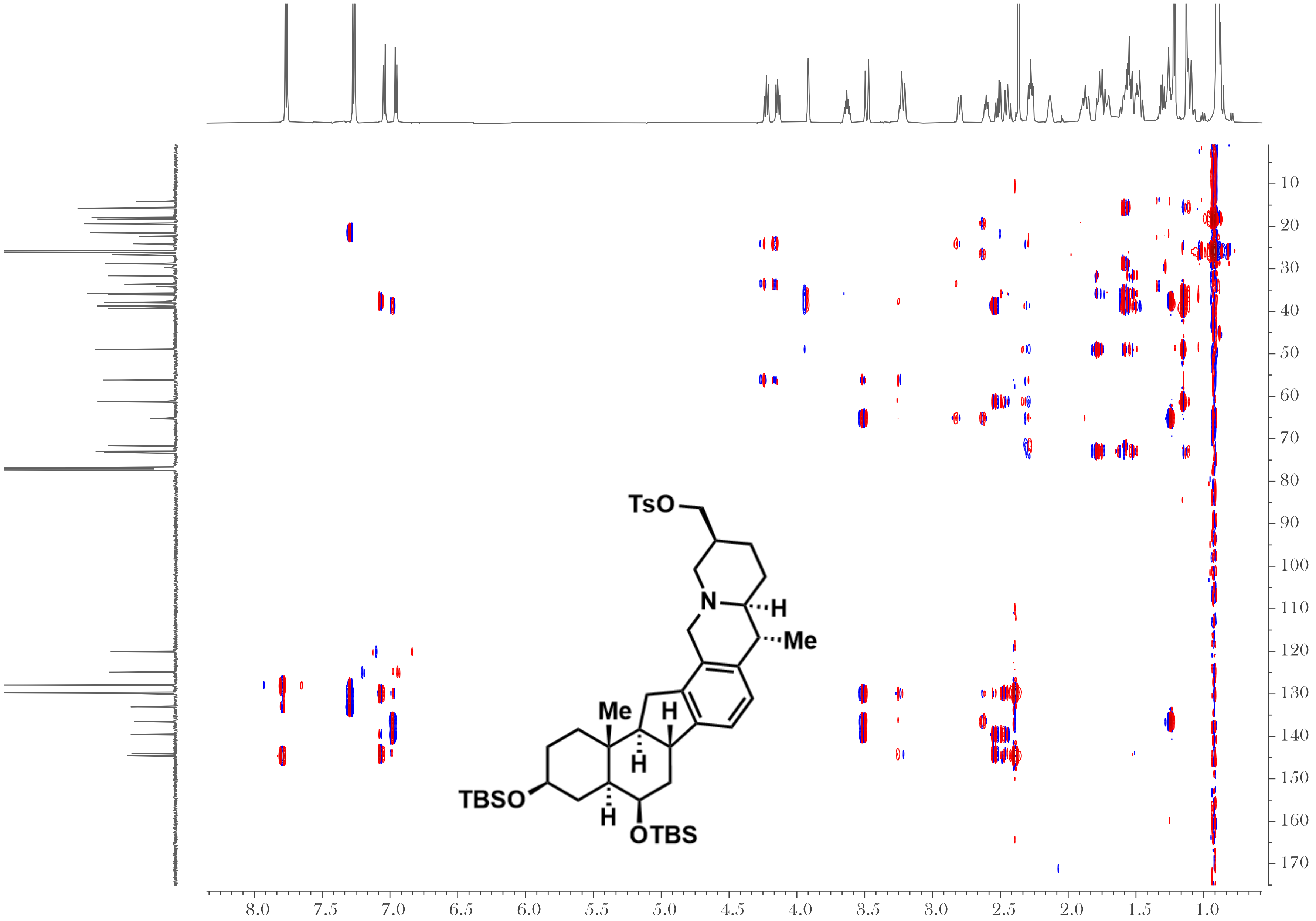

^1H–^{13}C HMBC Spectrum of **28** (150 MHz, $CDCl_3$, 25 °C)

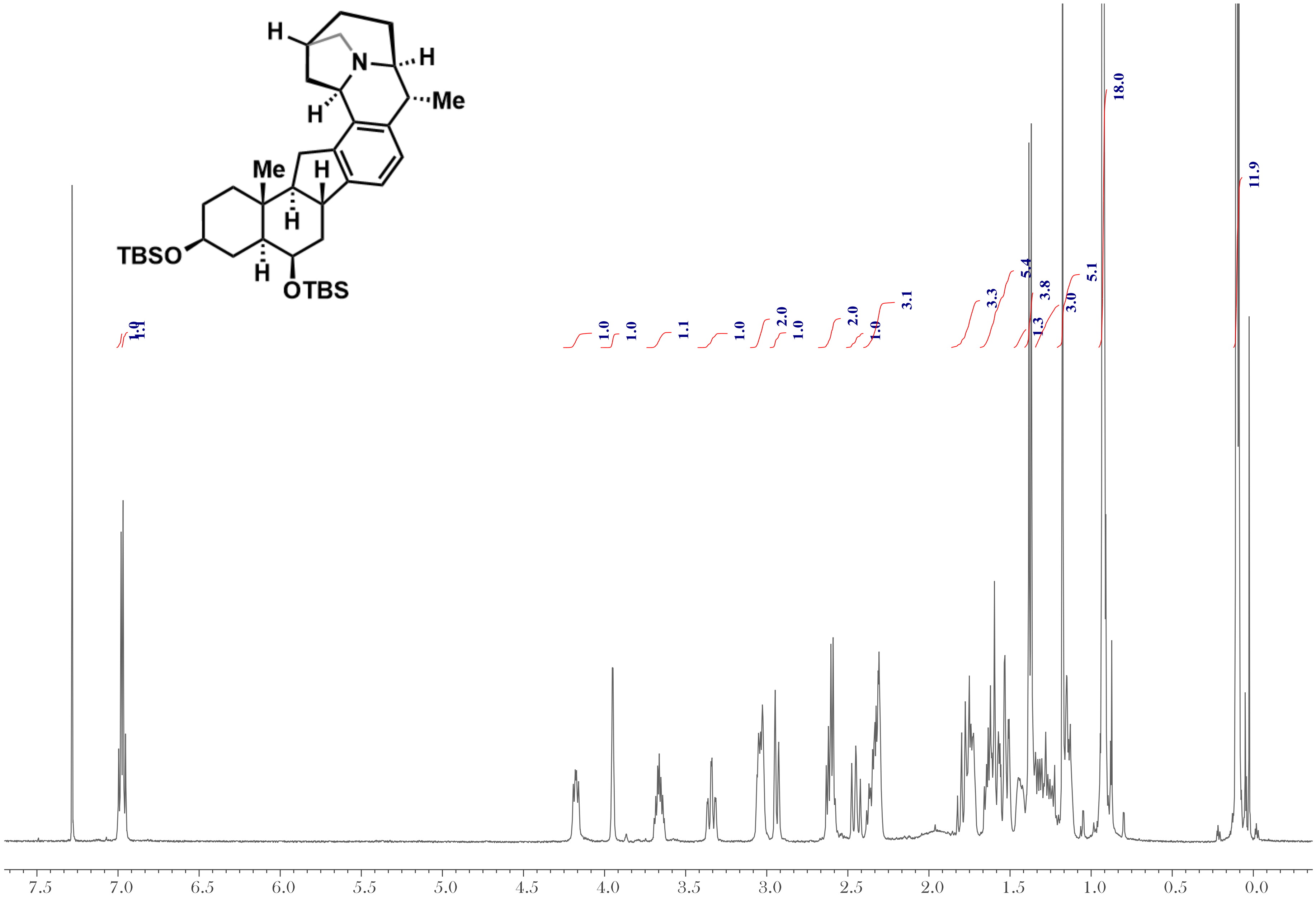

[1] H NMR Spectrum of **30** (500 MHz, $CDCl_3$, 25 °C)

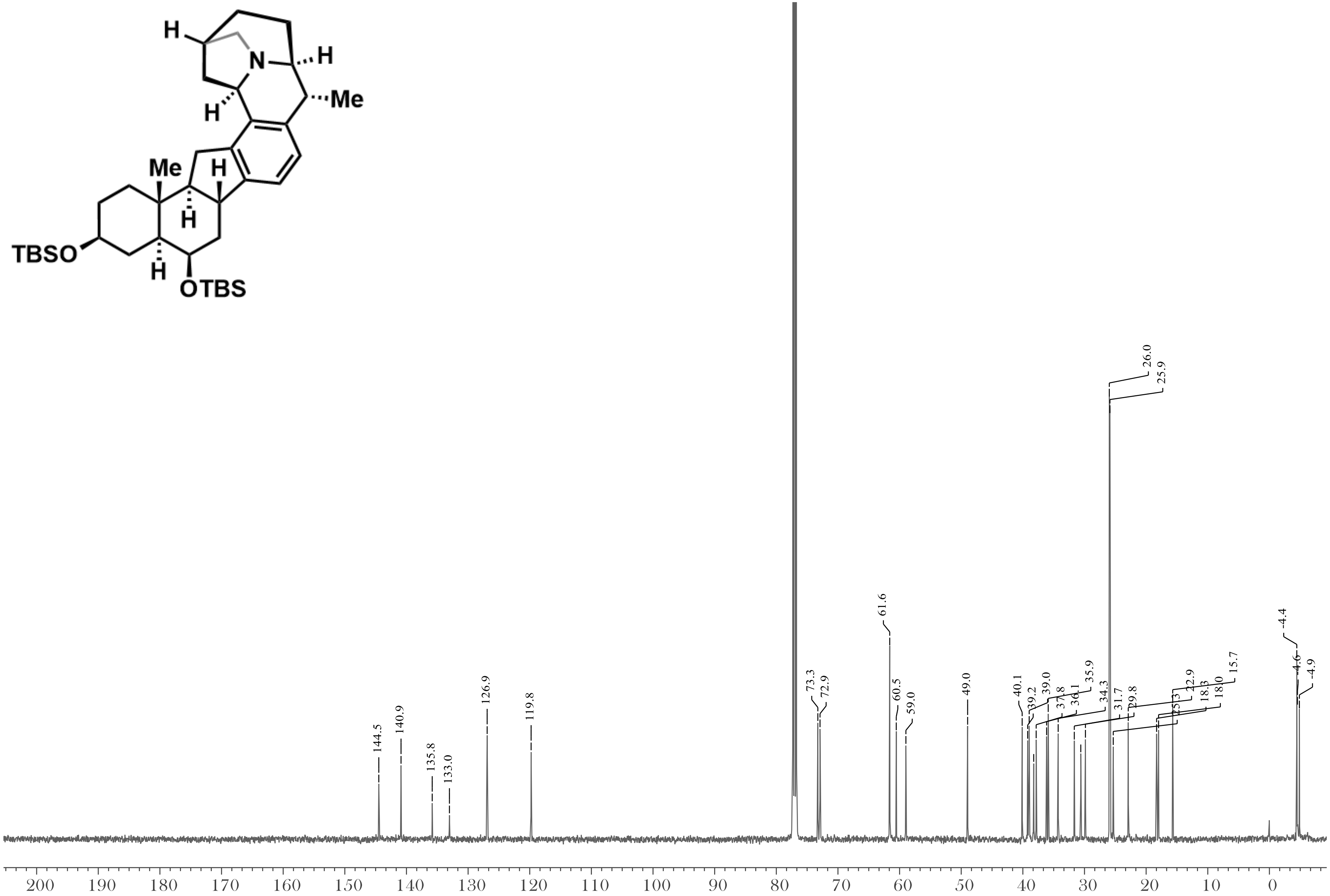

^{13}C NMR Spectrum of **30** (125 MHz, $CDCl_3$, 25 °C)

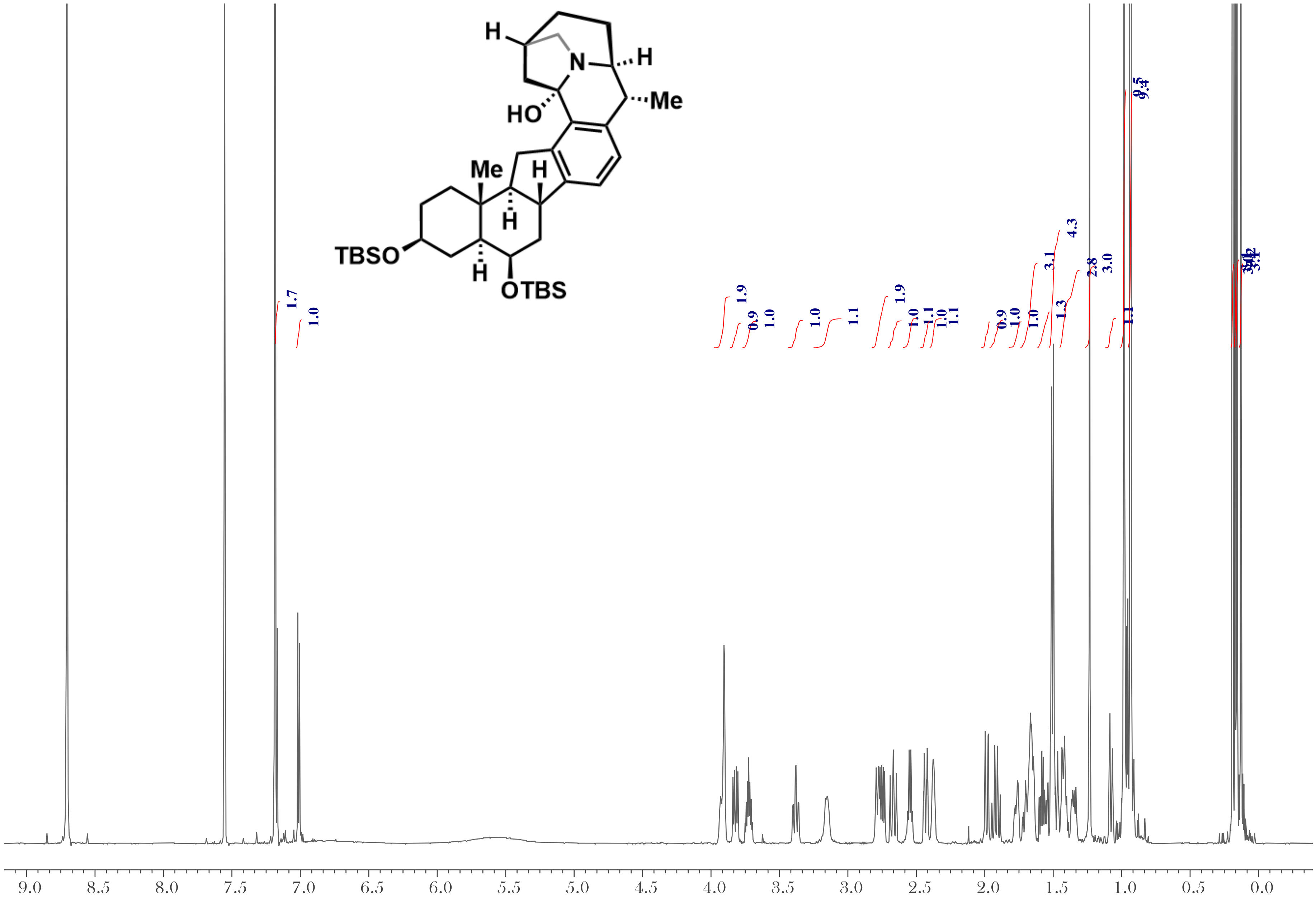

^{1}H NMR Spectrum of **32** (600 MHz, pyridine-d_5, 25 °C)

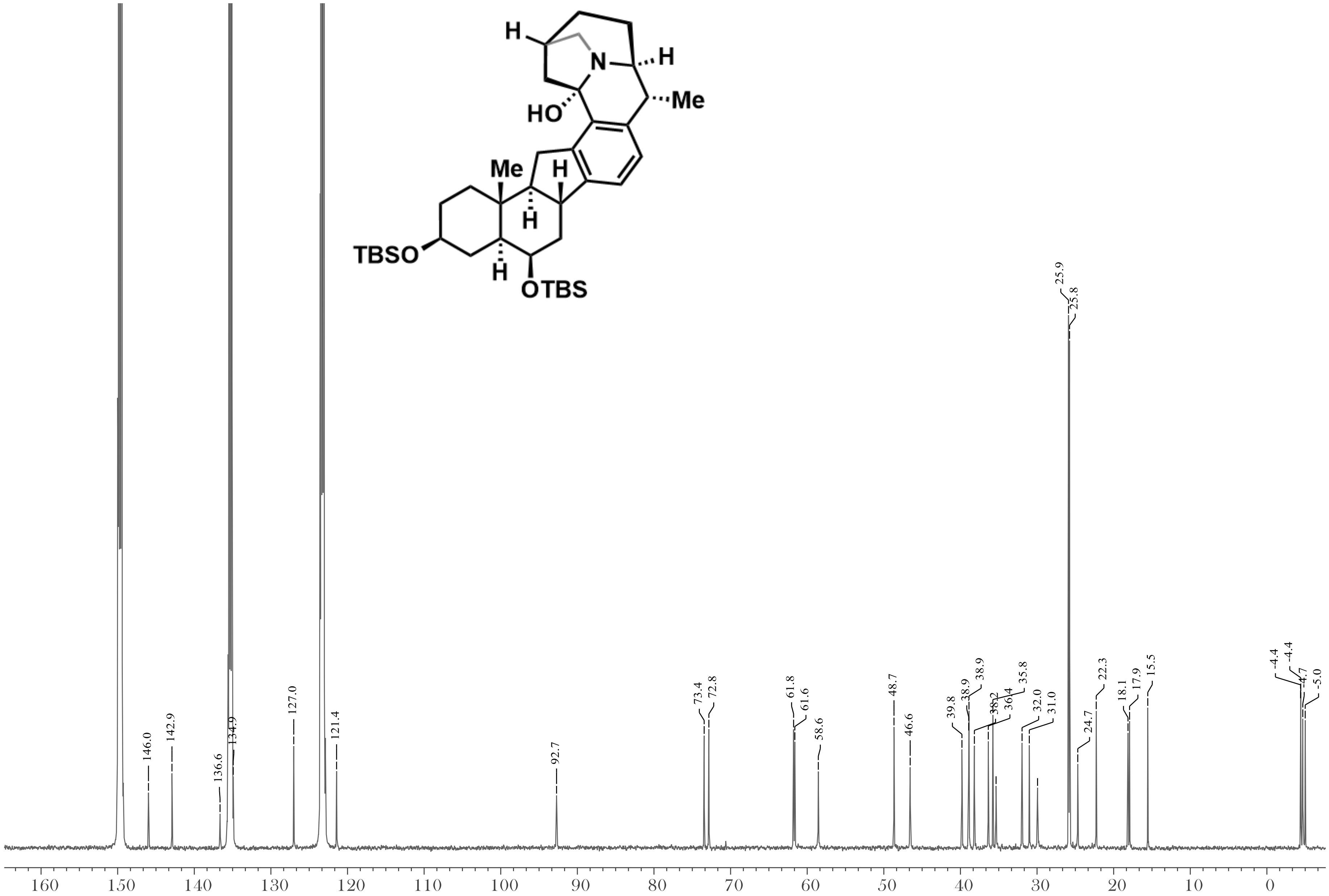

^{13}C NMR Spectrum of **32** (150 MHz, pyridine-d_5, 25 °C)

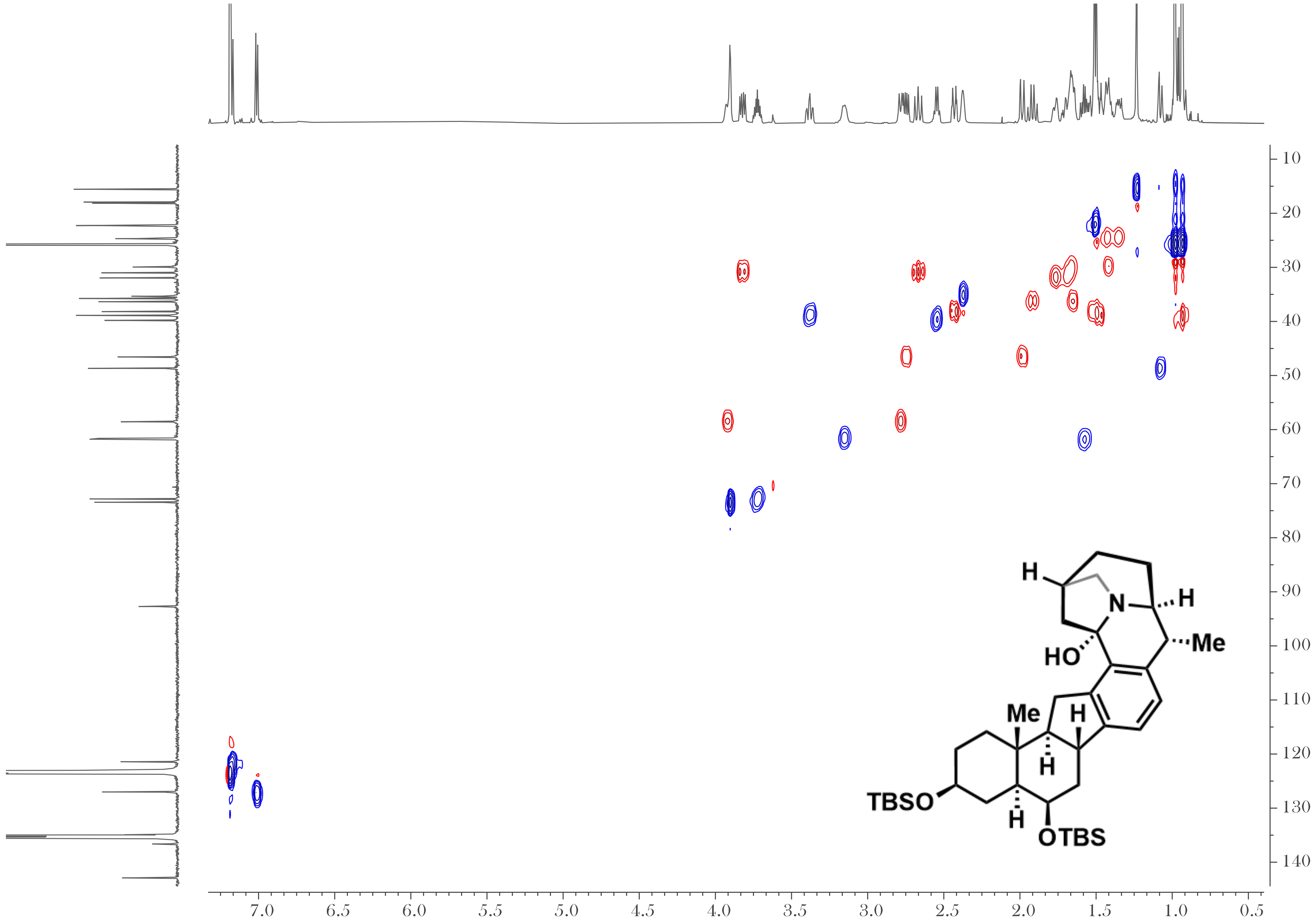

^1H–^{13}C HSQC Spectrum of **32** (150 MHz, pyridine-d_5, 25 °C)

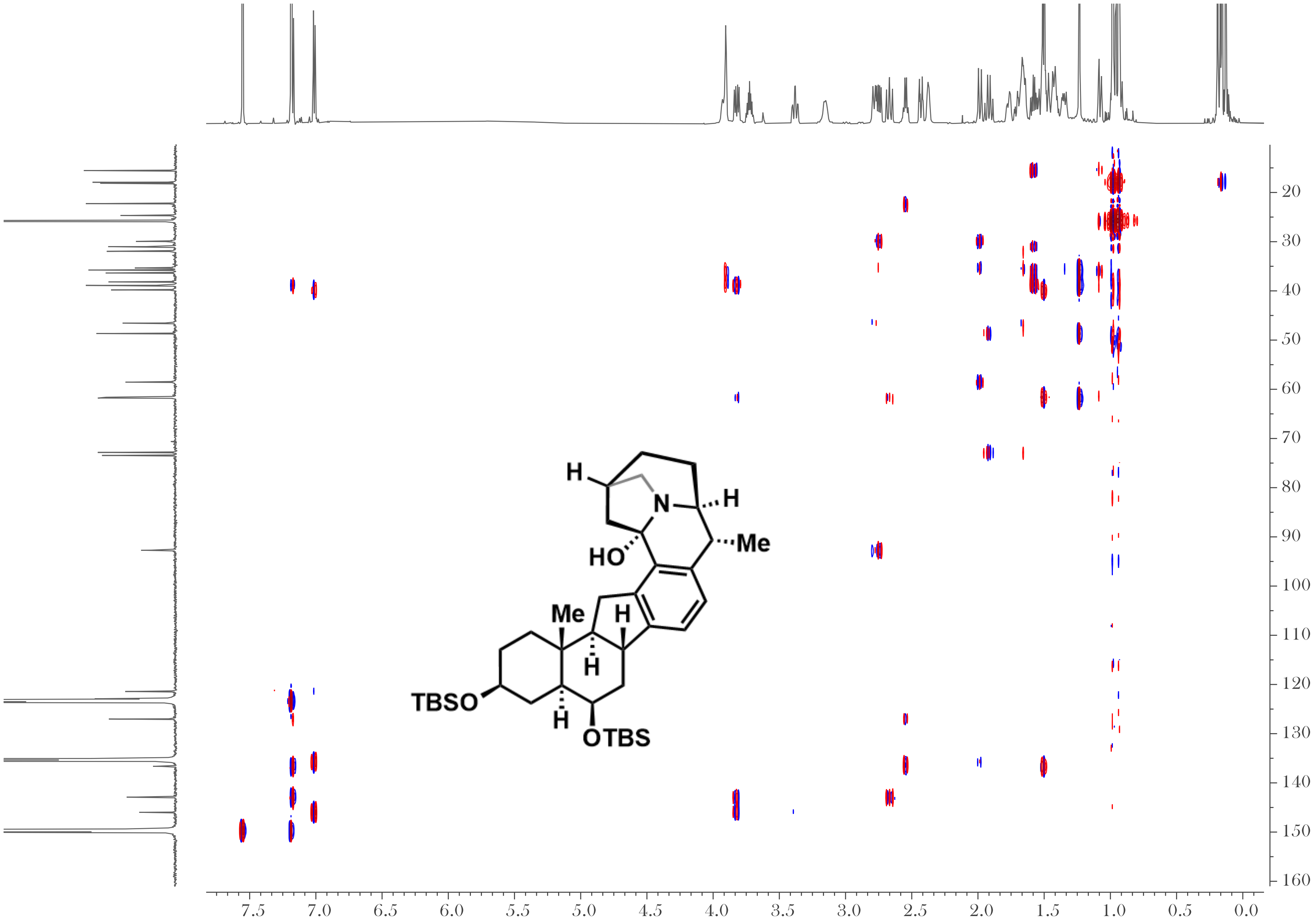

^{1}H–^{13}C HMBC Spectrum of **32** (150 MHz, pyridine-d_5, 25 °C)

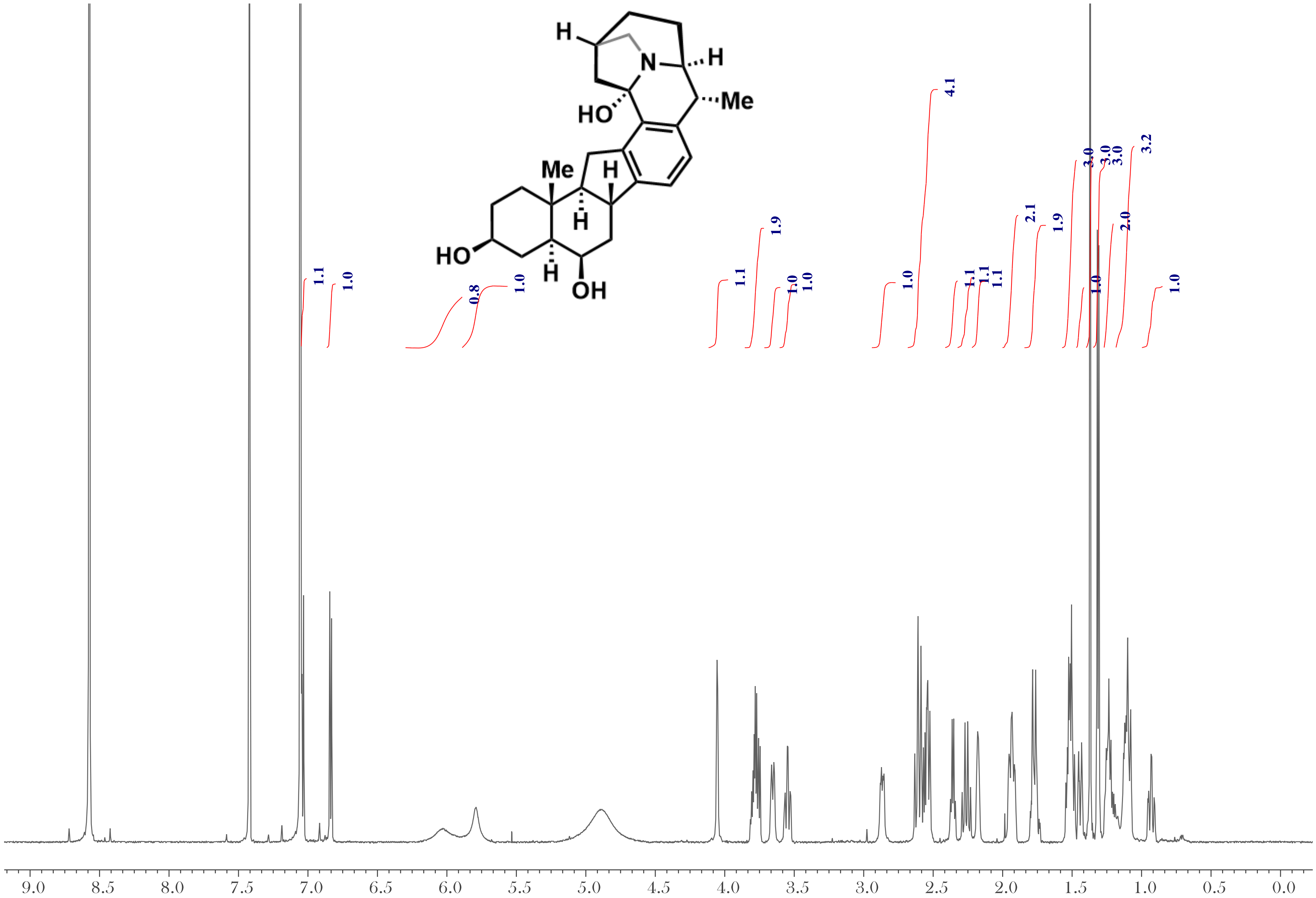

^{1}H NMR Spectrum of Ussuriedine (**1**, 600 MHz, pyridine-d_5, 25 °C)

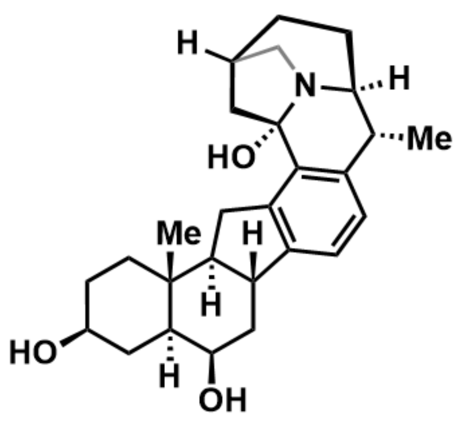

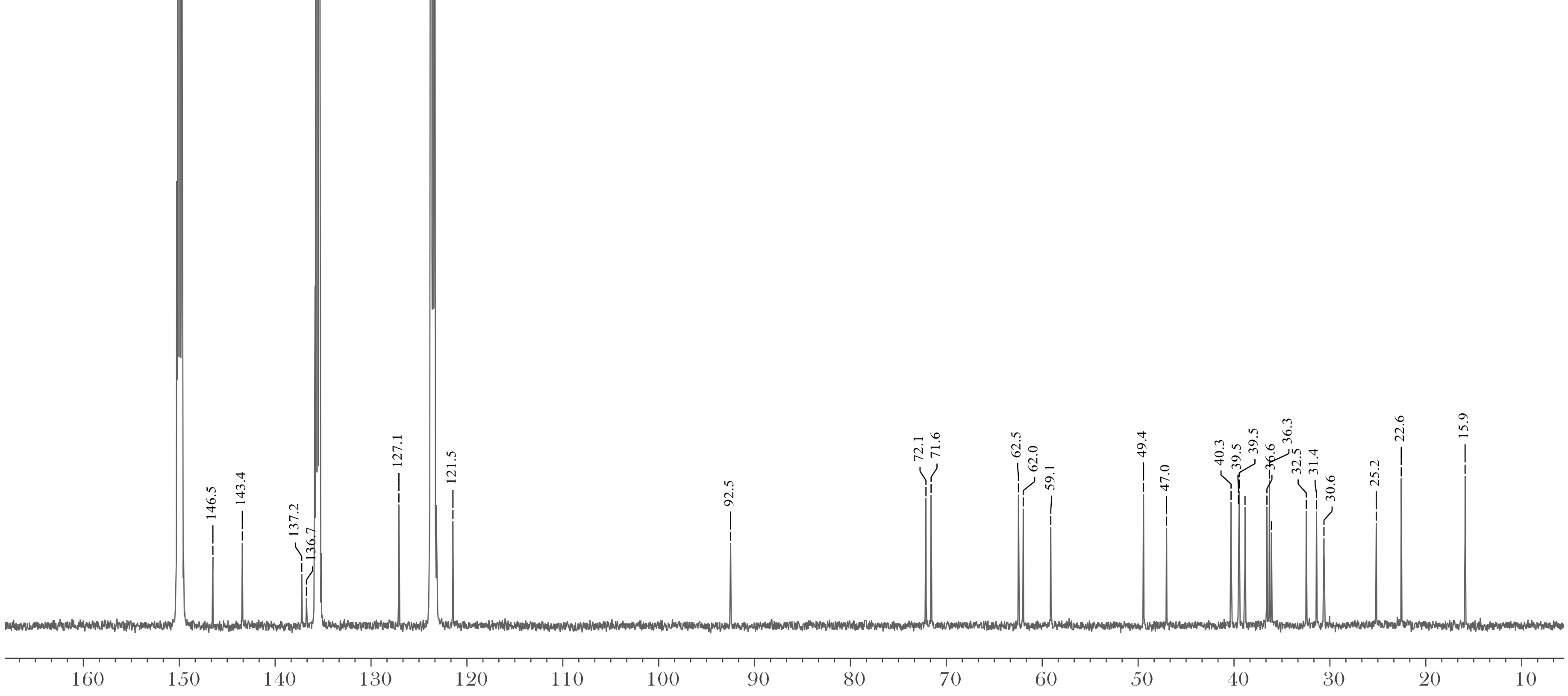

^{13}C NMR Spectrum of Ussuriedine (**1**, 150 MHz, pyridine-d_5, 25 °C)

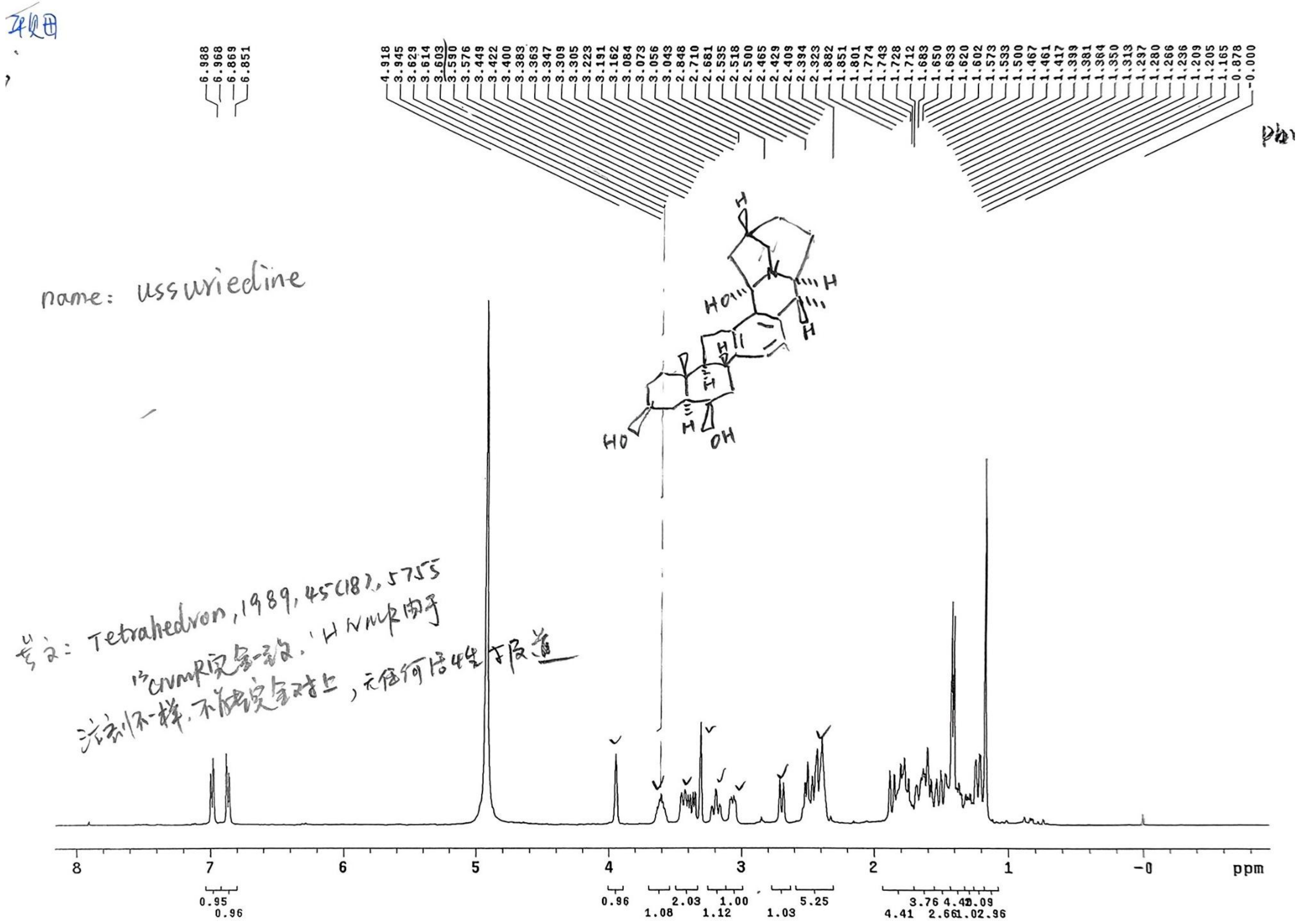

Yang's ^{1}H NMR Spectrum of Natural Ussuriedine (**1**, methanol-d_4, 25 °C)

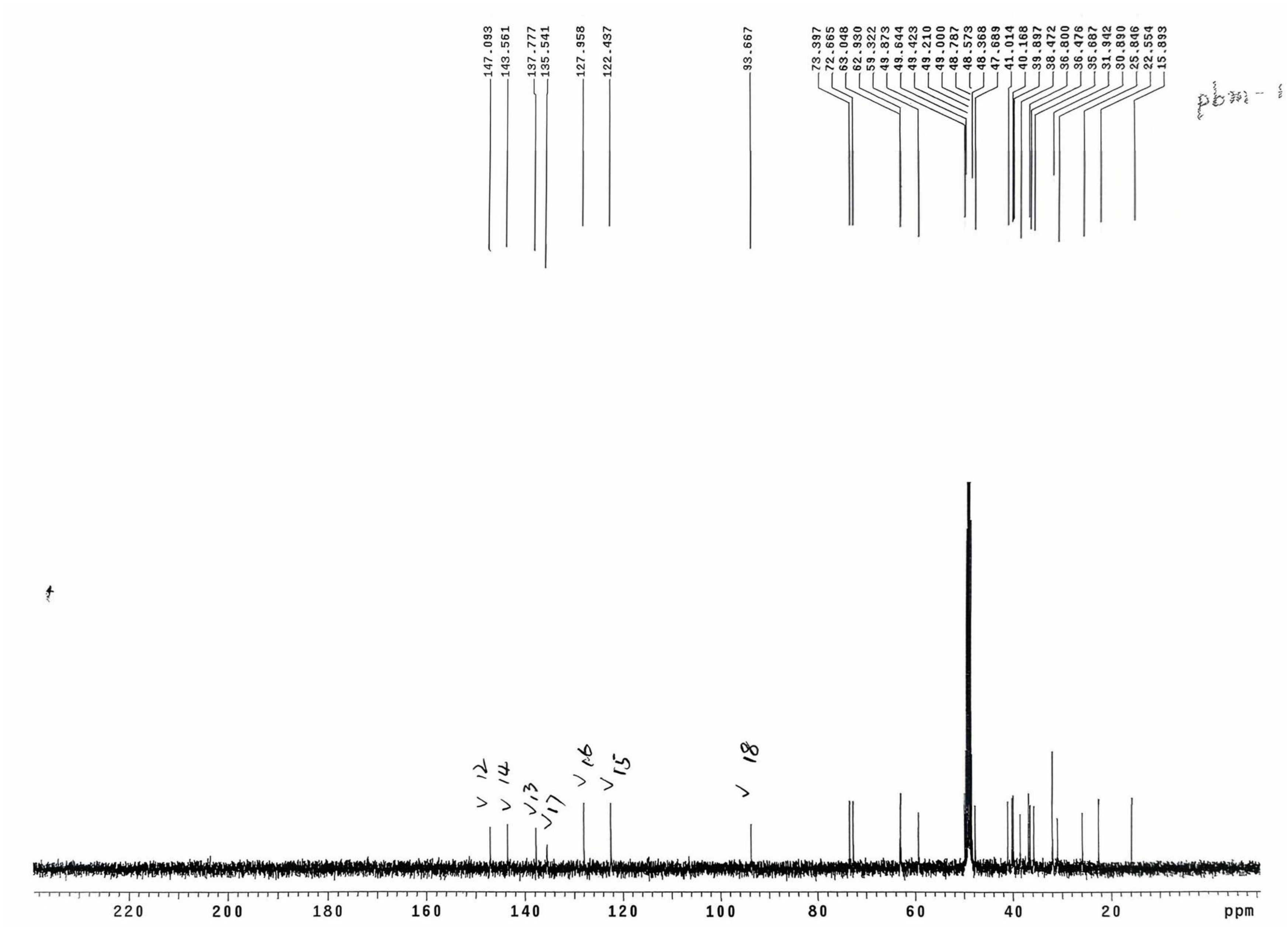

Yang's ^{13}C NMR Spectrum of Natural Ussuriedine (**1**, methanol-d_4, 25 °C)
